# Design, Synthesis, and Computational Evaluation of 3,4-Dihydroquinolin-2(*1H*)-One Analogues as Potential VEGFR2 Inhibitors in Glioblastoma Multiforme

**DOI:** 10.3390/ph18020233

**Published:** 2025-02-08

**Authors:** Shafeek Buhlak, Nadeem Abad, Jihane Akachar, Sana Saffour, Yunus Kesgun, Sevval Dik, Betul Yasin, Gizem Bati-Ayaz, Essam Hanashalshahaby, Hasan Türkez, Adil Mardinoglu

**Affiliations:** 1 Trustlife Labs, Drug Research & Development Center, Istanbul 34774, Turkey; shafeekbuhlak@outlook.com (S.B.); abadnadeem3@gmail.com (N.A.); jihane.akachar@trustlifelabs.com (J.A.); sasaffour@gmail.com (S.S.); yunus.kesgun@trustlifelabs.com (Y.K.); sevvaldik8@gmail.com (S.D.); btlyasin@hotmail.com (B.Y.); batigizem@gmail.com (G.B.-A.); essam.hanash@trustlifelabs.com (E.H.); 2 Department of Medical Biology, Faculty of Medicine, Atatürk University, Erzurum 25240, Turkey; hturkez@atauni.edu.tr; 3 Science for Life Laboratory, KTH-Royal Institute of Technology, SE-17165 Stockholm, Sweden; 4 Centre for Host-Microbiome Interactions, Faculty of Dentistry, Oral & Craniofacial Sciences, King’s College London, London SE1 9RT, UK

**Keywords:** glioblastoma multiforme, 3,4-dihydroquinolin-2(*1H*)-one, therapeutic efficacy, molecular docking, molecular dynamics, VEGFA–VEGFR2 pathway, anti-cancer

## Abstract

**Background/Objectives**: Glioblastoma multiforme (GBM), an aggressive and deadly brain tumour, presents significant challenges in achieving effective treatment due to its resistance to current therapies and poor prognosis. This study aimed to synthesise and evaluate 23 novel analogues of 3,4-dihydroquinolin-2(1*H*)-one, designed to enhance druggability and solubility, and to investigate their potential as VEGFR2 inhibitors for GBM treatment. **Methods**: The synthesised compounds were analysed using *in silico* methods, including molecular docking and dynamics studies, to assess their interactions with key residues within the VEGFR2 binding pocket. *In vitro* evaluations were performed on U87-MG and U138-MG GBM cell lines using MTT assays to determine the IC50 values of the compounds. **Results**: Among the tested compounds, **4u** (IC50 = 7.96 μM), **4t** (IC50 = 10.48 μM), **4m** (IC50 = 4.20 μM), and **4q** (IC50 = 8.00 μM) demonstrated significant antiproliferative effects against both the U87-MG and U138-MG cell lines. These compounds exhibited markedly higher efficacy compared to temozolomide (TMZ), which showed IC50 values of 92.90 μM and 93.09 μM for U87-MG and U138-MG, respectively. Molecular docking and dynamics studies confirmed strong interactions between the compounds and VEGFR2 kinase, supporting their substantial anti-cancer activity. **Conclusions**: This study highlights the promising potential of 3,4-dihydroquinolin-2(1*H*)-one analogues, particularly **4m**, **4q**, **4t**, and **4u**, as VEGFR2-targeting therapeutic agents for GBM treatment. Further detailed research is warranted to validate and expand upon these findings.

## 1. Introduction

**Figure 1:**
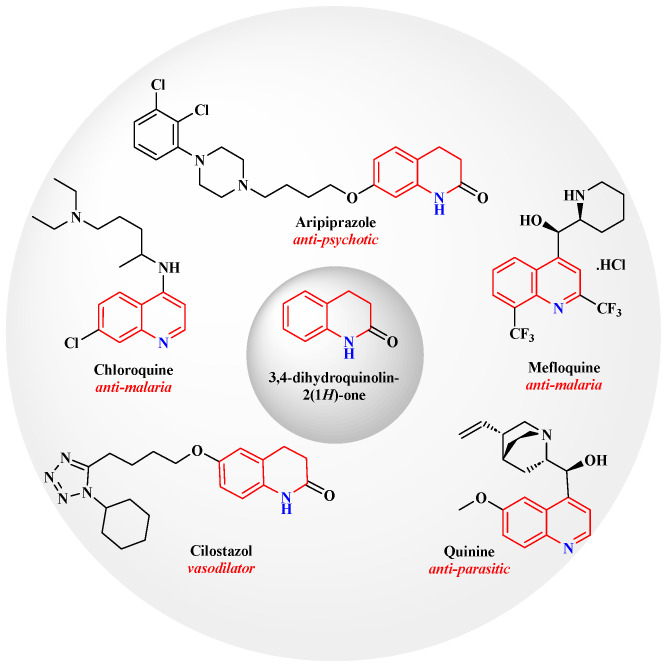
Some FDA-approved quinoline and quinolinone derivative drugs (tetrahydroquinoline core in red color).

Glioblastoma multiforme (GBM) is the most common and aggressive form of primary brain cancer, designated as a grade IV glioma. It encompasses a genetically and phenotypically diverse group of tumours characterised by rapid growth, widespread infiltration into surrounding brain tissue, and a notable resistance to existing therapies. Despite advancements in surgical techniques, radiation therapy, and chemotherapy, the prognosis for patients with GBM has not significantly improved, with median survival times lingering at 12–15 months over the last two decades [1,2]. The treatment of GBM faces several challenges, primarily due to the high heterogeneity of glioblastoma cells, which vary in cell type, density, size, mitotic activity, and the complex molecular mechanisms that drive their growth, vascularisation, necrosis, and survival. Another significant hurdle in treating GBM is the blood–brain barrier (BBB), a selective and sophisticated physiological barrier that regulates the passage of molecules between the brain and the bloodstream, effectively preventing drugs from reaching their target sites at therapeutic concentrations. Successful treatment strategies require therapeutic agents to penetrate the brain interstitial fluid, achievable either through receptor-mediated transport or lipid-mediated free diffusion across the BBB. Currently, almost all compounds in clinical trials for brain conditions are lipid-soluble small molecules with a molecular weight of 400–600 Da and low hydrogen bonding, typically equal to or less than seven hydrogen bonds with water as a solvent [3,4,5,6,7,8]. The therapeutic landscape for GBM has evolved significantly in recent years, marked by the emergence of novel treatments targeting specific molecular pathways within tumour cells. The conventional standard of care for GBM involves surgical removal of the tumour followed by concurrent chemotherapy and radiotherapy with temozolomide (TMZ) (Figure 1), an orally administered alkylating agent that is generally well-tolerated by most patients. However, the effectiveness of TMZ is limited by several factors. GBM cells frequently develop resistance to TMZ, largely due to the action of the repair enzyme O6-methylguanine-DNA-methyltransferase (MGMT). MGMT’s activity, which includes the induction of DNA methylation, impedes the replication of tumour cells, undermining the efficacy of TMZ. Additionally, the limited capacity of TMZ to cross the BBB and its associated toxicity at therapeutic doses underscore the need for alternative therapeutic approaches [9,10,11,12,13]. The ongoing challenges in GBM treatment, such as limited BBB penetration, resistance mechanisms, dosage constraints, and toxicity, necessitate the exploration of novel therapeutic strategies. This has led to increased research interest in small molecule drugs capable of crossing the BBB and offering more selective and potent therapeutic effects. The quinoline scaffold has received significant attention in pharmaceutical development for its diverse pharmacological properties [14,15,16,17]. Mefloquine (Figure 1), a compound initially known for its antimalarial effects, has shown promise in cancer treatment by inhibiting tumour cell growth [18]. Notably, studies investigating mefloquine’s ability to cross the BBB discovered that one of its enantiomers can do so in a stereoselective manner, highlighting its potential utility in treating brain tumours [19]. Previous studies have explored the relationship between antimalarial drugs and their effects on glioma cells, uncovering that quinine and chloroquine (Figure 1) exhibit notable anti-cancer properties. These compounds have been found to enhance sensitivity to TMZ and counteract resistance in newly diagnosed and recurrent TMZ-resistant gliomas through mechanisms like DNA replication interference and autophagy inhibition [20]. Additionally, aripiprazole (Figure 1), a drug primarily used for its antipsychotic effects in treating bipolar disorder and schizophrenia, has gained attention for its potential role in GBM therapy. Research conducted by Mi Seon et al. demonstrated that aripiprazole could reduce glioma U251 cell cytotoxicity by inhibiting the phosphorylation of Src and STAT3, impacting cell migration and apoptosis [21]. This suggests aripiprazole’s ability to modulate crucial signalling pathways involved in GBM progression, warranting further exploration of its therapeutic potential against this formidable brain cancer [22,23,24]. Concurrently, cilostazol (Figure 1), initially developed for cardiovascular conditions, has emerged as a compound of interest in GBM research. It has shown promise in delaying tumour growth in vivo by activating the calcium-activated potassium (BK) channel. The inactivation of this channel contributes to glioblastoma cells’ resistance to radiation, implying that reactivating the BK channel could enhance therapeutic outcomes, positioning cilostazol as a potential repurposed therapy for GBM [25]. One of the primary mechanisms leading GBM progression is angiogenesis, a process that encourages tumour vascularisation, mainly regulated by vascular endothelial growth factor receptor 2 (VEGFR2) and its ligand VEGF-A. Overexpression of VEGF-A in GBM leads to irregular blood vessel formation, boosting tumour growth, which subsequently accelerates the tumour’s invasive capacity. For these purposes, VEGFR2 was selected as a target gene based on prior research involving the designed peptide VGB4, known for its ability to bind to VEGFR2 and inhibit VEGF-A, thereby reducing cancer cell proliferation in the U87-MG glioblastoma cell line [26]. Targeting VEGFR2 has accordingly stood out as a critical therapeutic approach in the treatment of GBM [27,28]. There are many inhibitors for VEGFR2, such as avastin (bevacizumab), an anti-VEGF-A monoclonal antibody, which have been employed to slow down angiogenesis in GBM. However, the clinical efficacy of avastin has been limited due to the resistance development of GBM cells [29,30,31]. Sorafenib and sunitinib, small molecule VEGFR2 tyrosine kinase inhibitors (TKIs), block VEGFR2-mediated signalling, which is crucial for endothelial cell proliferation and migration [32,33]. However, their application in treating GBM has been restricted because of difficulties in penetrating the blood–brain barrier (BBB). These limitations emphasise the need for novel VEGFR2 inhibitors with improved BBB penetration and efficacy in targeting the growth of GBM cells. Leveraging insights from biological studies on existing derivatives, we aim to contribute to the ongoing search for innovative and effective treatments for GBM. Here, we focus on designing and synthesising novel 6-hydroxy-3,4-dihydroquinolin-2(1H)-one (Figure 1) derivatives. This scaffold has been shown to possess diverse pharmacological properties, including the possibility to inhibit VEGFR2. Hydrazone derivatives, in particular, have illustrated potency as VEGFR2 inhibitors, leading to improvements in their ability to induce cell death in GBM cell lines [34,35,36,37]. Moreover, research on the crystal structure of the VEGFR2 kinase domain in complex with PF-00337210 (N,2-dimethyl-6-(7-(2-morpholinoethoxy)quinolin-4-yloxy) benzofuran-3-carboxamide) has promoted our understanding of these interactions [38]. The discoveries presented in this research not only build upon existing knowledge but also pave the way for exploring new therapeutic strategies. By joining the collective effort to address the challenges posed by GBM, we hope to make a significant impact. A molecular docking study targeting VEGFR2 was conducted [39]. Additionally, the in silico ADME (Absorption, Distribution, Metabolism, and Excretion) properties of the derivatives indicated their potential to efficiently cross the BBB and exhibit antitumour activity within the central nervous system (CNS). This research highlights the significance of 6-hydroxy-3,4-dihydroquinolin-2(1H)-one derivatives as promising candidates for GBM therapy, contributing to the development of more selective and potent treatments for this devastating disease. This endeavour seeks to contribute to the ongoing search for more effective treatments for GBM, highlighting the potential of these novel compounds in the broader context of cancer research and therapy development.

## 2. Results and Discussion

### 2.1. Chemistry

**Scheme 1:**
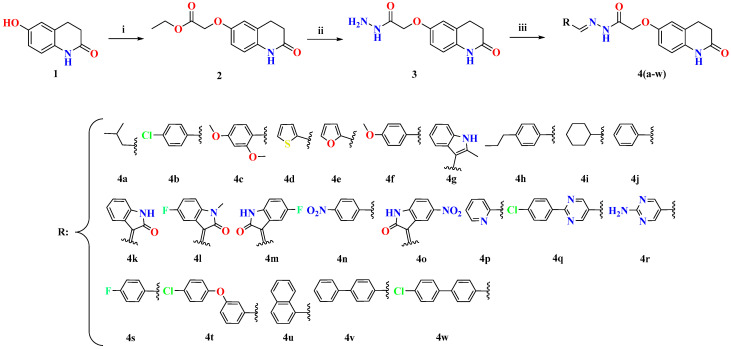
Synthesis of 2-((2-oxo-1,2,3,4-tetrahydroquinolin-6-yl)oxy)acetohydrazide-hydrazone analogues **4**(**a**–**w**). Reagents and conditions: (**i**) K_2_CO_3_, THF, DMF, and ethyl 2-bromoacetate; (**ii**) hydrazine hydrate and EtOH, at reflux; (**iii**) corresponding aldehydes or ketones in EtOH and acetic acid.

The synthesis of the designed compounds was accomplished using a carefully adapted chemical procedure. This procedure not only yielded the desired compounds but also provided opportunities for introducing diverse functional groups, each with potential implications for the efficacy of the compounds against GBM. We outline the synthesis process and examine the structural differences among these compounds, emphasising the impact of electron-withdrawing (EWG) and electron-donating groups (EDG) on their potential activity against cancer cells. The journey toward these new compounds represents a significant step in exploring novel therapeutic options. Scheme 1 started with the conversion of 6-hydroxy-3,4-dihydroquinolin-2(*1H*)-one in THF, with drops of DMF. The hydroxyl group was activated by stirring it with base-like potassium carbonate (K_2_CO_3_) or triethylamine (TEA), for one hour. Subsequently, ethyl 2-bromoacetate was added drop-wisely into the reaction mixture. At the end of this step, ethyl 2-((2-oxo-1,2,3,4-tetrahydroquinolin-6-yl)oxy)acetate was obtained. The subsequent transformation involved heating ethyl 2-((2-oxo-1,2,3,4-tetrahydroquinolin-6-yl)oxy)acetate with hydrazine hydrate (99%) in ethanol at 70 °C (reflux) to yield 2-((2-oxo-1,2,3,4-tetrahydroquinolin-6-yl)oxy)acetohydrazide. Finally, the 23 hydrazone compounds were synthesised by reacting various hydrazides with aldehydes/ketones in ethanol at 70 °C (reflux), with the addition of drops of acetic acid, to obtain 2-((2-oxo-1,2,3,4-tetrahydroquinolin-6-yl)oxy)acetohydrazide. The reactions were carried out at reflux temperature for 4–12 h.

The chemical structure of all the synthesised compounds was confirmed by LC-MS, ^1^H NMR, and ^13^C NMR spectral analysis (see Supplementary Materials). For compound **2**, the ^1^H NMR spectral exhibit quartet at δ 4.15 ppm related to methylene protons (O-C**H_2_**-CH_3_), and there was a triplet at δ 1.20 ppm corresponding to methyl protons (O-CH_2_-C**H_3_**). That means a new ethylene group was formed from compound **1** to compound **2**. These findings could be approved by ^13^C NMR spectral analysis, where there were new signals at δ 14.24 ppm and at δ 65.97 ppm, related to the methyl and methylene carbon, respectively. In addition, for compound **3**, the ethoxy group signals disappeared in ^1^H NMR and ^13^C NMR spectral analyses. Instead, there were new signals exhibited at δ 9.92 ppm for (C-N**H**-NH_2_) and at δ 4.31 ppm for (-N**H_2_**). The same finding was discovered for ^13^C NMR spectral analysis, where there were no signals for the ethoxy group that was in compound **2**. Related to the desire compounds **4**(**a**–**w**), there were new signals appearing in ^1^H NMR related to the methine group (N=C**H**-); for instance, compound **4a** exhibited a multiplet between δ (6.67–6.64) ppm and singlets at δ (8.33, 8.58, 8.18, 8.24, and 8.28) ppm, related to the compounds **4b**, **4c**, **4d**, **4e**, and **4f**, respectively. For ^13^C NMR spectral analysis, the signal of methine carbon appeared at δ 144.2 ppm and at δ 169.76 ppm for the compounds **4j** and **4v**, respectively, as examples of the new methine carbon signal. Observing the molecular ion peaks of the mass spectra of all compounds, they conformed to the M + 1 or M − 1 peak of the appropriate molecular formula. For example, the [M + 1]^+^ at *m*/*z* 249.96, 357.91, 366.20, and 397.12 were in accordance with the molecular formulae of **2**, **4b**, **4h**, and **4l** (C_13_H_15_NO_4_, C_18_H_16_ClN_3_O_3_, C_21_H_23_N_3_O_3_, and C_20_H_17_FN_4_O_4_), respectively. Moreover, for [M − 1]^−^ at *m*/*z* 363.34, 381.10, 367.21, 408.26, they were in accordance with the molecular formulae of **4k**, **4m**, **4n**, and **4o** (C_19_H_16_N_4_O_4_, C_19_H_15_FN_4_O_4_, C_18_H_16_N_4_O_5_, and C_19_H_15_N_5_O_6_), respectively.

### 2.2. In Vitro Studies

#### 2.2.1. Efficacy of Novel Analogues on Cell Proliferation

**Figure 2:**
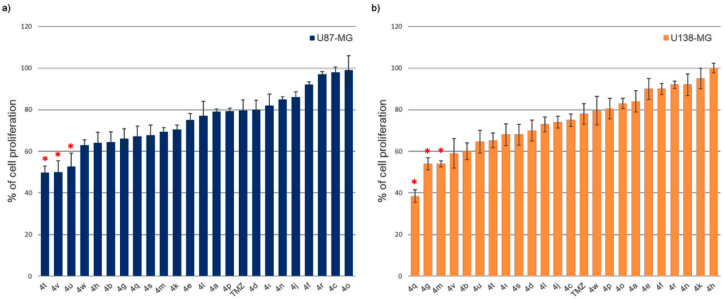
Cell proliferation of glioblastoma cell lines for 48 h of compound treatment: (**a**) U87-MG and (**b**) U138-MG. (*) indicates to most active compounds.

**Figure 3:**
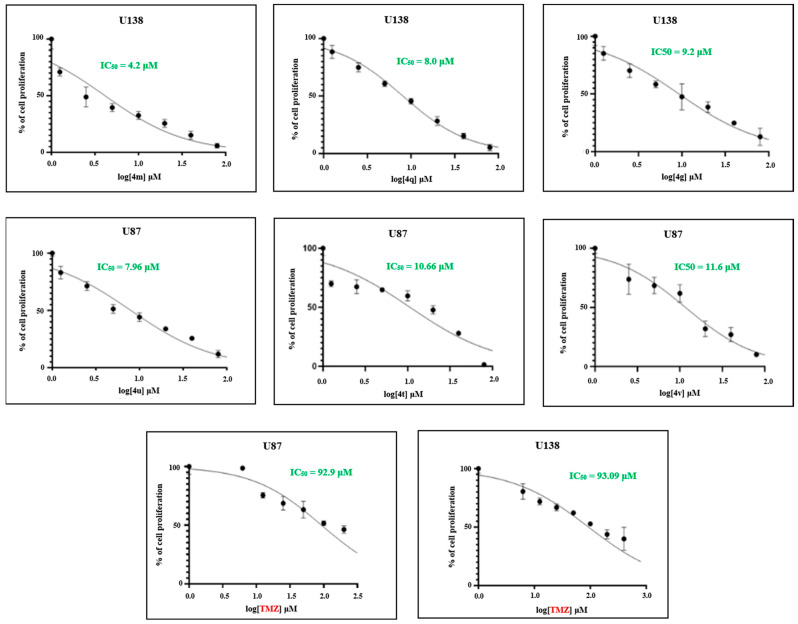
IC_50_ values of the best active molecules and TMZ for both glioblastoma cell lines.

U87-MG and U138-MG glioblastoma cell lines were used to evaluate the in vitro effects of the **4**(**a**–**w**) compounds on glioblastoma treatment. The effect of the compounds on cell proliferation was measured. Among them, the compounds **4t**, **4u**, and **4v** exhibited the highest antiproliferative activity on the U87-MG cell line, while the compounds **4q**, **4m**, and **4g** showed excellent results on the U138-MG cell line (Table 1, Figure 2 and Figure 3).

IC_50_ values were calculated depending on the increasing compound concentration (0–160 μM) for both glioblastoma cell lines. The most effective compounds for decreasing cell proliferation were **4u** (IC_50_ = 7.96 μM), **4t** (IC_50_ = 10.66 μM), and **4v** (IC_50_ = 11.66 μM) for U87-MG, and **4m** (IC_50_ = 4.20 μM), **4q** (IC_50_ = 8.00 μM), and **4g** (IC_50_ = 9.20 μM) for the U138-MG cell line (Figure 3). The decrease in cell proliferation was detected with the increase of the compound concentration.

#### 2.2.2. Permeability of Novel Analogues Through Biological Membrane Mimics of the Blood–Brain Barrier (BBB) and Gastrointestinal (GI) Membrane

The permeability of the synthesised compounds was evaluated using the parallel artificial membrane permeability assay (PAMPA) to estimate their ability to cross biological membrane mimics of both the gastrointestinal (GI) membrane and the blood–brain barrier (BBB).

The results showed that the compounds **4j**, **4t**, and **4h** exhibited high permeability across the BBB-mimicking membrane, proposing their potential to effectively penetrate the CNS. In contrast, the remaining compounds showed lower permeability across the BBB, suggesting that further structural optimisation may be necessary to enhance the penetration of the compounds in the CNS. However, for those demonstrating low PAMPA permeability, this could be attributed to the PAMPA model complexity. PAMPA uses a simple phospholipid-based artificial membrane that may not fully mimic the dynamic nature of the BBB in vivo, especially regarding active efflux mechanisms, tight junctions, and protein binding interactions.

**Figure 4:**
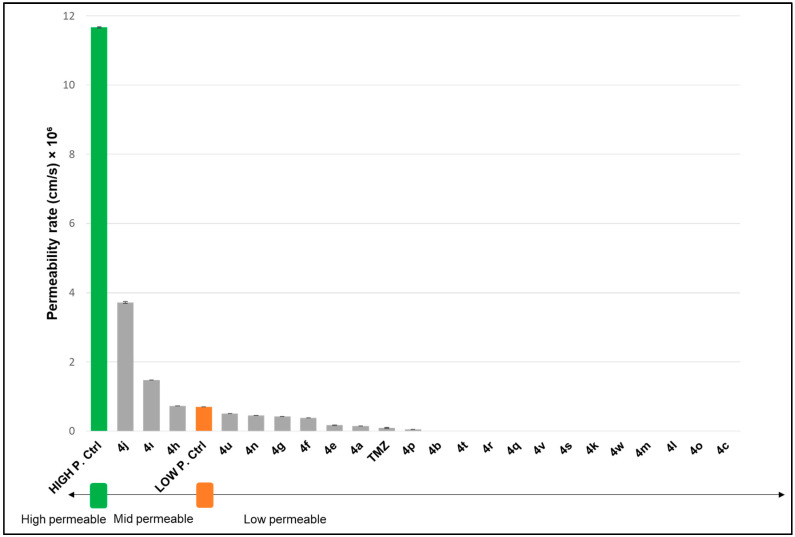
Permeability rates of compounds through the blood brain barrier (BBB)-mimicking membrane (HIGH P. Ctrl: high permeability control, LOW P. Ctrl: low permeability control, TMZ: temozolomide).

**Figure 5:**
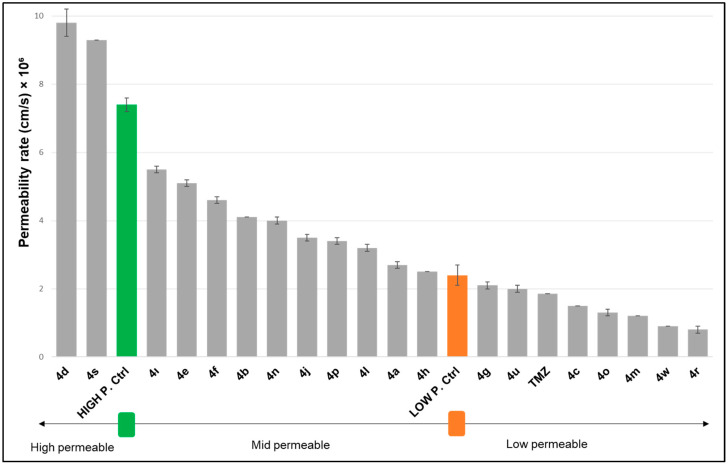
Permeability rates of compounds through the gastrointestinal (GI)-mimicking membrane (HIGH P. Ctrl: high permeability control, LOW P. Ctrl: low permeability control, TMZ: temozolomide).

Comparably, the GI permeability assay revealed that the compounds **4d** and **4s** showed high permeability across the gastrointestinal membrane, indicating their potential for efficient oral bioavailability. The remaining compounds demonstrated lower permeability, proposing possible limitations in their oral absorption profiles. These findings highlighted the importance of balancing hydrogen bonding, hydrophobicity, and molecular size to improve GI permeability. Remarkably, TMZ, used as a reference control, exhibited lower permeability in both the BBB and GI assays, consistent with its known pharmacokinetic profile (Figure 4 and Figure 5).

### 2.3. In Silico Studies

#### 2.3.1. Molecular Docking Studies

**Figure 6:**
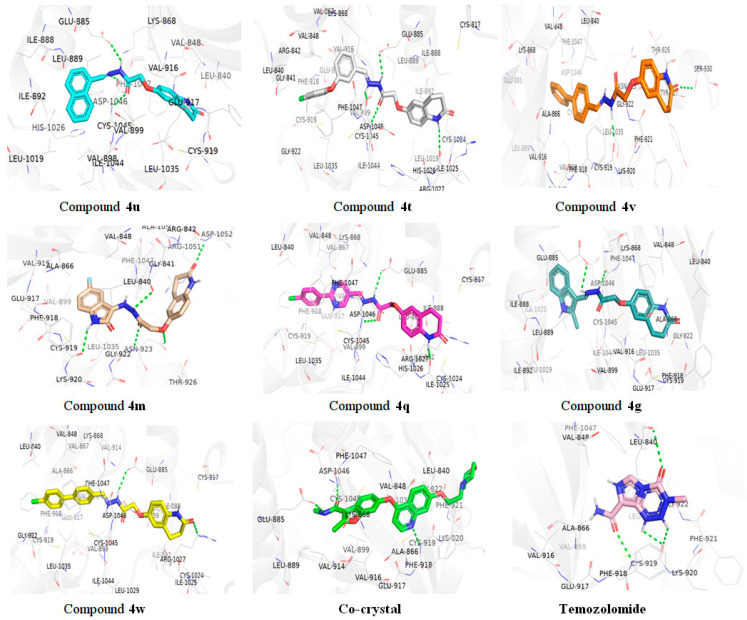
Molecular docking analysis of predicted drugs targeting VEGFR2. Putative binding modes of the best compounds **4u**, **4t**, **4v**, **4m**, **4q**, **4g**, **4w**, and temozolomide against VEGFR2 with the co-crystal PF-00337210 (*N*,2-dimethyl-6-(7-(2-morpholinoethoxy)quinolin-4-yloxy)benzofuran-3-carboxamide) as control. VEGFR2 is represented in a gray lines representation and hydrogen bonding residues highlighted by green dotted lines.

**Figure 7:**
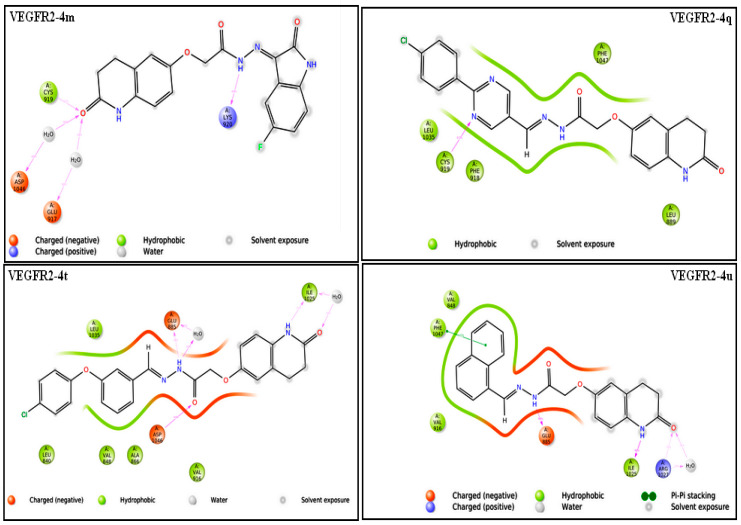
Diagram illustrating interactions between ligand atoms and protein residues of VEGFR2 complexed with the compounds **4m**, **4q**, **4t**, and **4u**.

We conducted out molecular docking studies to have a thorough understanding of the binding method of the 3,4-dihydroquinolin-2(*1H*)-one derivatives, and TMZ as a reference, with the target protein VEGFR2, which plays a key role in angiogenesis—the formation of new blood vessels from existing ones. Targeting VEGFR2 with inhibitors can reduce angiogenesis and help restore blood–brain barrier (BBB) function. However, it may also have side effects by potentially increasing the permeability of the BBB to harmful substances [35,40,41]. The predicted docking scores for the binding interactions of the 3,4-dihydroquinolin-2(1H)-one derivatives and TMZ with VEGFR2 are presented in Table 2. The docking scores for the top compounds (**4w**, **4t**, **4u**, **4q**, and TMZ) were (−12.2, −11.4, −11.4, −11.1, and −6.2) kcal/mol, respectively, indicating moderate to strong interactions (Table 2). Additionally, it was observed that the top derivatives exhibited predominantly hydrophilic interactions, similar to the co-crystal ligand PF-00337210 (N,2-dimethyl-6-(7-(2-morpholinoethoxy)quinolin-4-yloxy)benzofuran-3-carboxamide), with the active site residues in the VEGFR2 kinase domain. These interactions involved key amino acids, such as Asp1046, Glu885, and Ile1025, alongside several important hydrophobic interactions (Figure 6 and Figure 7). Meanwhile, compound **4m** displayed five hydrogen bonds with Asp1052, Leu840, Asn923, and Cys919, with a docking score of −9.9 kcal/mol, sharing common interacting residues with TMZ. Most of the synthesised 3,4-dihydroquinolin-2(1H)-one analogues demonstrated good binding affinity toward VEGFR2. Notably, the IC50 values observed for these compounds (**4u**: 7.96 μM, **4t**: 10.48 μM, **4m**: 4.20 μM, and **4q**: 8.00 μM) highlight their significant potency in reducing glioblastoma (GBM) cell viability, with compound **4m** exhibiting the lowest IC50, correlating with its strong docking score. In comparison, the standard treatment TMZ exhibited much higher IC50 values (92.90 μM for U87-MG and 93.09 μM for U138-MG), consistent with its weaker binding interactions in the docking studies.

Overall, the in silico findings and in vitro results underscore the promising anti-cancer potential of these 3,4-dihydroquinolin-2(1H)-one analogues, particularly the compounds **4m**, **4q**, **4t**, and **4u**.

#### 2.3.2. Molecular Dynamics Simulation

**Figure 8:**
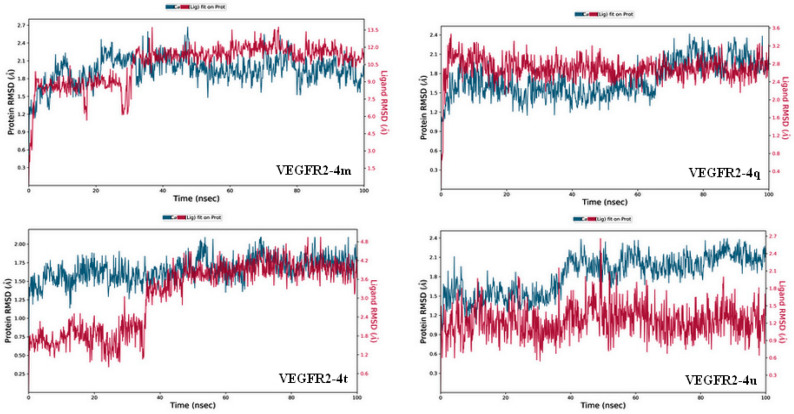
RMSD analysis of the protein and ligands over 100 ns of molecular dynamics simulation.

To investigate the dynamic behaviour and stability of VEGFR2–3,4-dihydroquinolin-2(1H)-one derivative complexes, molecular dynamics (MD) simulations were conducted over a 100 ns period. This study focused on evaluating the structural changes induced by the best compounds with a good docking score and number of H-bond interactions, with insight into **4m**, **4q**, **4t**, and **4u**. The stability of each protein–ligand complex was monitored through RMSD analysis throughout the simulation. Figure 8 illustrates the RMSD values: the left *Y*-axis represents the RMSD of the protein, while the right *Y*-axis shows the ligand RMSD profile aligned with the protein backbone. Frames from the 100 ns trajectory were aligned with a reference frame to assess stability. The RMSD plot reveals that the VEGFR2–**4m** complex achieved stability after 20 ns, the VEGFR2–**4q** complex stabilised after 5 ns, although a minor increase in ligand RMSD was noted at 65 ns, the VEGFR2–**4t** complex became stable after 15 ns, and the VEGFR2–**4u** complex reached stability after 2 ns, with a slight rise in ligand RMSD observed at 40 ns. Such deviations could be linked to the high number ofthe ligand’s rotatable bonds.

The root mean square fluctuation (RMSF) measures the average deviation of each atom’s position from its mean position across a given simulation or set of structures. It provides valuable insights into the flexibility or mobility of residues, with higher RMSF values indicating greater flexibility or movement. In our study, some residues exhibited elevated RMSF values, indicating increased flexibility and notable impact. For instance, the residues at positions **860**, **873**, and **1064** showed the highest RMSF values in the dataset—**2.2**, **3.4**, and **4.0**, respectively—suggesting significant flexibility in the VEGFR2–**4q** complex compared to the other derivatives.

Conversely, residues with lower RMSF values, such as those between positions **840**–**848**, **868**, and **1028**, which correspond to the binding and active sites of VEGFR2, ranged from **0.5** to **1.0**, reflecting relatively low flexibility and greater rigidity.

Notably, key residues of the protein consistently maintained interactions with all 3,4-dihydroquinolin-2(*1H*)-one derivatives (**4m**, **4q**, **4t**, and **4u**). Residues involved in ligand binding were associated with lower RMSF values, indicating stable interactions, while residues not involved in binding exhibited higher RMSF values, highlighting their increased mobility.

Figure 8 illustrates the interactions between the compounds **4m**, **4q**, **4t**, and **4u** with the VEGFR2 active site pocket, observed for over 30% of the simulation duration. In the VEGFR2–4m complex, four hydrogen bonds were identified: one between **Cys919**, a water molecule, and the hydroxyl group, and another between **Lys920** and the amino group. Additionally, negative charge interactions occurred between **Glu917**, **Asp1046**, and the water molecule and hydroxyl group of **4m**. For the **VEGFR2–4q** complex, one hydrogen bond was formed between **Cys919** and the amino group, with hydrophobic interactions observed involving **Leu889**, **Cys919**, **Phe918**, **Leu1035**, and **Phe1047**. In the VEGFR2–4t complex, five hydrogen bonds were established: between **Glu885**, **Ile1025**, and the amino group, as well as between **Asp1046**, a water molecule, and the hydroxyl group. Hydrophobic interactions were observed with **Ile1025**, **Val916**, **Ala866**, **Val848**, **Leu840**, and **Leu1035**. For the VEGFR2–4u complex, four hydrogen bonds were identified: between **Glu885**, **Ile1025**, and the amino group, as well as between **Arg1027**, a water molecule, and the hydroxyl group. Additionally, a π–π interaction was noted between **Phe1047** and the aromatic group of **4u**, along with hydrophobic interactions involving **Leu840**, **Phe1047**, **Val916**, and **Ile1025**. The underlined amino acids represent residues involved in the ATP interaction site of the VEGFR2 receptor (ID: **P35968 VGFR2_HUMAN**).

**Figure 9:**
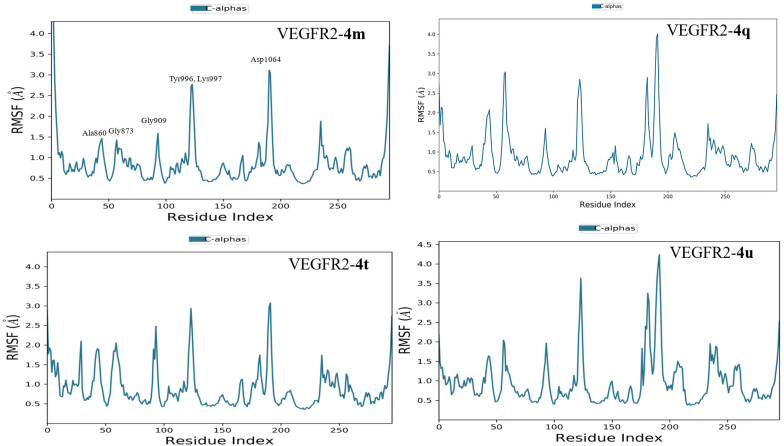
Residue-level fluctuations of VEGFR2 complexed with the compounds **4m**, **4q**, **4t**, and **4u**.

**Figure 10:**
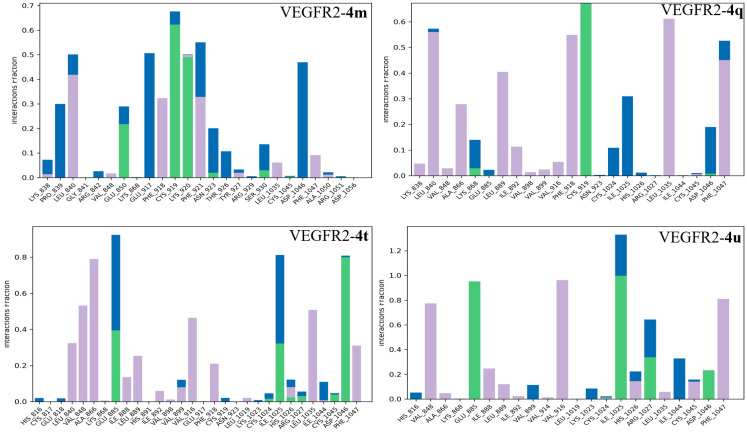
Protein–ligand interaction dynamics throughout the simulation period of VEGFR2 complexed with the compounds **4m**, **4q**, **4t**, and **4u**. Hydrogen bonds, hydrophobic contacts, ionic interactions, and water bridges are colored green, purple, pink, and blue, respectively.

Figure 9 illustrates the energy components of the non-covalent interactions observed during the simulation. The *X*-axis represents the residues at the active site that interact with the ligand, while the *Y*-axis indicates the proportion of the simulation time during which these interactions occur. The stacked bar charts are normalised across the entire simulation trajectory. As shown in Figure 9 and Figure 10, the ATP binding site residue Leu840 participated in hydrophobic interactions with the compounds **4m**, **4q**, and **4t** for approximately 42%, 58%, and 33% of the simulation time, respectively. Similarly, Leu848 engaged in hydrophobic interactions with **4m**, **4q**, **4t**, and **4u** for around 2%, 2%, 53%, and 78% of the simulation duration, respectively. The ATP binding site residue Leu868 exhibited hydrophobic interactions with **4m**, **4t**, and **4u** for about 1% of the simulation time each, and formed hydrogen bonds with **4q** for approximately 3%. Furthermore, Cys919 formed hydrogen bonds with **4m** and **4q** for over 65% of the simulation period, while Asp1046 showed hydrogen bonding with 4t and **4u** for 80% and 23% of the time, respectively. Ile1025 interacted with **4t** and **4u** for 33% and 100% of the simulation time, respectively. Other interacting residues displayed various types of interactions, including ionic and water-bridged interactions with the ligand. As a result, these residues were involved in multiple interactions throughout the simulation. Molecular dynamics simulations further confirmed that the compounds **4u**, **4t**, **4m**, and **4q** maintained stable interactions with VEGFR2 over time, supporting their potential to inhibit VEGFR2 activity and reduce GBM cell viability. Notably, compound 4m exhibited the lowest IC50, which correlated with its stable binding observed in the simulations.

#### 2.3.3. In Silico ADME Studies

A computational study of all 23 molecules **4**(**a**–**w**) was conducted to predict their ADME properties using the QikProp3.2 tool that is available in Schrödinger and SwissADME, and the PreADME online server. Key parameters were selected, relevant to BBB permeability, using the QikProp3.2 tool available in Schrödinger (Table 2). logBB values for all molecules were found to be within a favourable range, indicating their potential for BBB penetration. MDCK cell permeability (QPPMDCK) is considered a good in vitro mimic for BBB permeability, with values < 25 indicating poor permeability and values > 500 indicating excellent permeability. Most of the tested molecules showed MDCK values within the optimal range, with the compounds **4b**, **4p**, **4t**, and **4w** exhibiting values > 300, demonstrating good BBB penetration. In contrast, only three compounds (**4n**, **4o**, and **4r**) displayed poor permeability, suggesting limited BBB transport potential.

Additionally, the drug-likeness prediction results for all derivatives were evaluated based on Lipinski’s “Rule of Five”, confirming that the majority of the compounds met the established criteria for oral bioavailability and drug-likeness [42].

All four principal parameters for the derivatives—H-bond donors (<5), H-bond acceptors (<10), LogP (<5), and molecular weight (<500 Da)—were within the acceptable ranges for drug-like compounds [43]. According to Jorgensen’s “Rule of Three”, the compounds should also meet the following criteria: QPlogS > −5.7, QPPCaco > 22 nm/s, and primary metabolites < 7. The blood–brain barrier (BBB) permeability of the compounds was calculated using SwissADME and PreADME, with the obtained data summarised in Table 3 and Table 4. All parameter values were found to be within the specified limits, indicating that the 3,4-dihydroquinolin-2(1H)-one derivatives have the potential to effectively cross the BBB.

### 2.4. Structure–Activity Relationship (SAR) Exploration of Compounds (**4a**–**4w**) Against U138-MG Glioblastoma Cells

Bioactivity analysis focused on explaining the description of relationships between molecular structures and the remarked biological activities against the U138-MG glioblastoma cell line, compared against **TMZ**. With understanding gathered from the molecular docking studies against the VEGF receptor, notable trends emerge to describe the flexibility in compound potency modulation. The compounds **4m** (IC_50_ = 4.20 μM), **4q** (IC_50_ = 8.00 μM), and **4g** (IC_50_ = 9.20 μM) exhibit significant cell inhibition percentages, underscored by their respective docking scores (−9.9 kcal/mol, −11.1 kcal/mol, and −10.3 kcal/mol). Molecular docking interprets key interactions, in which hydrogen bonding with critical residues such as Asp1046 and Glu885 confers favourable binding affinities. Noteworthy trends appear, highlighting the complexity that controls the compound potency. The compounds **4e** (10%) and **4d** (30%), which have a furan and thiophen moiety, respectively, display heightened bioactivity, underscoring the significance of specific structural motifs as heterocyclic structures. The *N′*-(3-methylbutylidene) group in **4a** contributes to moderate activity (16%). The presence of the 3-methylbutylidene moiety may lead to the observed bioactivity. The *N′*-((2-methyl-1*H*-indol-3-yl)methylene) group in **4g** (46%) positively influences activity, suggesting the significance of aromatic functionalities. The *N′*-([1,1′-biphenyl]-4-ylmethylene) moiety in **4v** (41%) enhances activity, suggesting a role for extended aromatic systems. However, the presence of a chloro-substituent in **4w** that has a *N′*-((4′-chloro-[1,1′-biphenyl]-4-yl)methylene) group leads to a notable decrease in bioactivity (20%) compared to **4v**. The 4-chlorobenzylidene group in **4b** (40%) provides high activity, meaning that chlorine substitution might enhance interactions with the target, leading to increased bioactivity. Replacing chlorine by fluorine in the *N′*-(4-fluorobenzylidene) group in **4s** (32%) may enhance electronic effects and thus lead to the drop in its cytotoxic activity of 8%, and its docking score can relate to the same aspect, with docking scores of −10.3 and −9.6 for **4b** and **4s**, respectively. However, the replacement of halide with methoxy in the compound **4c** (25%), which has a *N′*-(2,4-dimethoxybenzylidene) group, provides moderate activity by emphasising the relevance of an electron-donating group (EDG). On the other hand, **4f** (10%) has the presence of one with the lowest docking score, −8.8, and comparing with **4b** and **4c**, its methoxy group on the para position in the *N′*-(4-methoxybenzylidene) group leads to reduced activity. Interestingly, compound **4h** (0%), lacking noticeable activity, suggests that 4-propylbenzylidene moiety may be unfavourable for bioactivity. The *N′*-benzylidene group in **4j** exhibits moderate activity (26%). The presence of the benzylidene group may contribute to favourable interactions. Replacing the phenyl group with cyclohexyl in the *N′*-(cyclohexylmethylene) group in **4i** shows moderate activity (32%). The cyclohexylmethylene substituent may contribute to favourable bioactivity. The presence of the 3-(4-chlorophenoxy) benzylidene group in **4t** exhibits moderate activity (35%), and may contribute to favourable interactions with the target. Replacing the benzylidene group by the naphthalen-1-ylmethylene substituent in **4u** exhibits moderate activity (35%). The *N′*-(4-nitrobenzylidene) group in **4n** shows limited activity (8%), suggesting that the nitro substituent as EWG may not boost bioactivity. Moreover, this group could sometimes be less favourable for cellular absorption. Seemingly, in 4r (8%), the 2-aminopyrimidin-5-yl group shows very low activity, meaning that this group may not be optimal for bioactivity. The presentation of the nitrobenzylidene group in **4o** resulted in moderate activity, proposing a potential role for electron-withdrawing substituents. On the contrary, the N*′*-(5-fluoro-2-oxoindolin-3-ylidene) group in **4m** contributes to high activity (46%). The presence of the fluoro and 2-oxoindolin-3-ylidene groups may enhance bioactivity through favourable interactions with five H-bonds with the important residues Asp1052, Leu840, Asn923, and Cys919. Contrarily, the combination of a pyridinylmethylene group in **4p** yielded analogous effects, indicating the relevance of deferent substituent effects. Compound **4q** (61%), containing a chlorophenylpyrimidinylmethylene group, exhibited a remarkable boost in activity, surpassing **TMZ**.

A computational and in vitro study was conducted on the most promising molecules—**4g**, **4m**, and **4q**—in comparison with temozolomide (TMZ) (Table 5). The key parameters, including molecular docking scores, IC50 values, and ADME properties, were found to be within optimal ranges, supporting the potential therapeutic value of these 3,4-dihydroquinolin-2(1H)-one derivatives and confirming their promise as effective candidates for further investigation. This highlights the importance of heterocyclic moieties with varied electronic properties. The findings from this bioactivity analysis provide a roadmap for targeted structural modifications, offering guiding principles for further optimisation and rational drug design efforts. These discoveries offer valuable insights into the development of novel therapeutics for glioblastoma, contributing to advancements in precision medicine.

## 3. Materials and Methods

### 3.1. Materials

Reagents and solvents were obtained from Sigma-Aldrich and Ambeed and were used without further purification. Reactions were monitored via LC-MS (Thermo Fisher TSQ Series, Athena C18-WP column, Waltham, MA USA, water with 0.01% formic acid) or TLC (silica gel, Kieselgel 60, E. Merck, Germany). Proton NMR (^1^H NMR) spectra were recorded on an Advance Bruker 500 MHz spectrometer (Bruker, Billerica, MA, USA) using CDCl_3_ and DMSO-*d*_6_, with data reported in δ/ppm relative to TMS, including multiplicity, hydrogen count, and coupling constants (J in Hz). Carbon-13 NMR (^13^C NMR) data were also given in δ/ppm. Chemical shifts use the residual solvent peak as a reference, with standard abbreviations for signal patterns. Flash column chromatography was conducted on a PuriFlash XS 520 Plus system, Montluçon, France, using (Interchim) silica gel columns, with UV and ELSD detection and fraction collection.

### 3.2. General Procedures and Spectral Data

Acylation reactions were initiated as the primary step (**i**), wherein compounds containing hydroxy/amino groups (**1**) (1 eq.) and the base K_2_CO_3_ (1.5 eq.) or triethylamine TEA (1.5 eq.) were treated with acyl chloride derivatives in THF, acetone, or DMF. This process yielded acylated products [44]. Upon completion of the reaction, solvents were evaporated, residues were washed with water, dried, and subjected to purification to obtain the corresponding acyl compounds (**2**).

The second step (**ii**) involved the synthesis of hydrazide products (**3**). This was achieved by refluxing acetate-containing compounds (1 eq.) and hydrazines (2 eq.) in ethanol. After the reaction reached completion, the product was cooled to room temperature and filtered out of ethanol. The final products (**4**) were obtained in the third and last step (**ii**) through Schiff base reactions. Hydrazones were formed by reacting hydrazides and aldehydes/ketones in ethanol with drops of acetic acid under reflux conditions for 4–12 h. After cooling, the product was filtered and purified using column chromatography.

#### 3.2.1. Synthesis of Ethyl 2-((2-Oxo-1,2,3,4-Tetrahydroquinolin-6-Yl) Oxy)Acetate (**2**)

A total of 250 mg of 6-hydroxy-3,4-dihydroquinolin-2*(1H)*-one (1.23 mmol) was stirred with 317.61 mg of the base K_2_CO_3_ (2.3 mmol) for 1 h in THF and drops of DMF. Then, 0.204 mL (1.51 g/cm^3^) of ethyl 2-bromoacetate (1.5 mmol) was added after workup, and column chromatography (Hexane:EtOAc) (50:50) then afforded 313 mg (81%) as white crystals. m.p: 139.4, LC-MS: *m*/*z* 249.96 [M + H]^+^. **^1^H-NMR** (500 MHz, DMSO-*d*_6_) δ 9.93 (s, 1H. N-H), 6.79–6.70 (m, 3H_arom_), 4.68 (s, 2H. O=C-C**H_2_**-O), 4.15 (q, *J* = 7.1 Hz, 2H. O-C**H_2_**-CH_3_), 2.82 (t, *J* = 7.5 Hz, 2H. O=C-CH_2_-C**H_2_**-), 2.39 (m, 2H. 2H. O=C-C**H_2_**-), 1.20 (t, *J* = 7.1 Hz, 3H. C**H_3_**). **^13^C-NMR** (126 MHz, DMSO) δ 172.07, 169.05, 153.81, 131.93, 125.13, 116.47, 114.93, 113.41, 65.97, 61.44, 30.54, 25.66, 14.24.

#### 3.2.2. Synthesis of 2-((2-Oxo-1,2,3,4-Tetrahydroquinolin-6-Yl)Oxy)Acetohydrazide (**3**)

A total of 300 mg of compound **2** (1.2 mmol) was heated with hydrazine hydrate 99% (3.6 mmol) in ethanol at 70 °C. After workup, the product was obtained as white powder with a yield of 90%. m.p: 210–211 °C, **^1^H-NMR** (500 MHz, DMSO-*d*_6_) δ 9.92 (s, 1H. N-**H**), 9.27 (s, 1H. O=C-N-**H**), 6.81–6.73 (m, 3H_arom_), 4.40 (s, 1H. O-C**H_2_**-C=O), 4.31 (s, 2H. N**H_2_**), 2.83–2.80 (m, 2H. O=C-C**H_2_**-), 2.41–2.38 (m, 2H. O=C-CH_2_-C**H_2_**). **^13^C-NMR** (126 MHz, DMSO) δ 170.22, 167.19, 153.44, 132.75, 125.22, 116.13, 114.76, 113.72, 67.16, 30.77, 25.56.

#### 3.2.3. Synthesis of (E)-N′-(3-Methylbutylidene)-2-((2-Oxo-1,2,3,4-Tetrahydroquinolin-6-Yl)Oxy)Acetohydrazide (**4a**)

According to the mentioned general procedure, starting from 6-hydroxy-3,4-dihydroquinolin-2(*1H*)-one, 300 mg of 6-hydroxy-3,4-dihydroquinolin-2(1H)-one (1.84 mmol) was stirred with 381 mg of the base K_2_CO_3_ (2.76 mmol) for 15 min in THF and drops of DMF, then 244 μL of ethyl 2-bromoacetate (2.21 mmol) was added slowly to the reaction mixture. After the completion of the reaction, the solvent was evaporated and the residue was washed with water, filtered, and air-dried to obtain ethyl 2-((2-oxo-1,2,3,4-tetrahydroquinolin-6-yl)oxy)acetate (**2**), which was further refluxed with 241 μL hydrazine hydrate 99% (4.81 mmol) for 5 h. It was then cooled to room temperature, filtered out of ethanol, and dried to obtain 2-((2-oxo-1,2,3,4-tetrahydroquinolin-6-yl)oxy)acetohydrazide, compound **3**. Later, 300 mg of compound **3** (1.28 mmol) was refluxed with 168 μL of isovaleraldehyde (1.53 mmol) in ethanol and drops of acetic acid for 5 h. After that, the precipitate was filtered out of ethanol to obtain the final product as white powder with a yield of 85%. m.p: 197–199 °C, LC-MS: *m*/*z* 304.11 [M + H]^+^. **^1^H-NMR** (500 MHz, DMSO-d6) δ 11.08 (s, 1H. C-NH-N=), 9.93 (* 9.95, s, 1H. C-NH-C), 7.63–6.72 m, 3H**_arom_**), 6.67–6.64 (m, 1H. N=C**H**-), 4.87 (s, 1H. O-**CH_2_**-C=O), 4.48 (s, 1H. O-C**H_2_**-C=O), 2.84–2.80 (m, 2H), 2.43–2.36 (m, 2H), 2.12–2.06 (m, 2H), 1.84–1.78 (m, 1H), 0.91 (d, J = 2.9 Hz, 3H), 0.90 (d, *J* = 2.9 Hz, 3H). (* refer to the rotameric peak). **^13^C-NMR** (125 MHz, DMSO) δ 169.80, 163.86, 151.89, 147.77, 132.43, 131.99, 124.75, 114.41, 113.28, 66.90, 40.53, 30.32, 26.14, 25.12, 22.29, 22.26.

#### 3.2.4. Synthesis of (E)-N′-(4-Chlorobenzylidene)-2-((2-Oxo-1,2,3,4-Tetrahydroquinolin-6-Yl) Oxy) Acetohydrazide (**4b**)

A total of 150 mg of compound **3** (0.8 mmol) was heated with 134.45 mg of 4-chlorobenzaldehyde (0.956 mmol) in ethanol under reflux with the presence of AcOH (2 drops). The desired molecule was precipitated when the reaction finished (controlled by TLC) and the solvent was evaporated, and the obtained compound was washed by ethanol to obtain 210 mg (92%) as white powder. m.p:244–246 °C, LC-MS: *m*/*z* 357.91 [M + H]^+^. **^1^H-NMR** (500 MHz, DMSO-*d*_6_) δ 11.62 (* 11.59, s, 1H. C-**NH**-N=), 9.96 (* 9.92, s, 1H. C-N**H**-C), 8.33 (* 7.99, s, 1H. N=C**H**-) 7.74–6.72 (m, 7H**_arom_**), 5.07 (s, 1H. O-**CH_2_**-C=O), 4.60 (s, 1H. O-**CH_2_**-C=O), 2.86–2.81 (m, 2H. C**H_2_**CH_2_-C=O), 2.42–2.38 (m, 2H. C**H_2_**C=O). (* refer to the rotameric peak). **^13^C-NMR** (125 MHz, DMSO) δ 170.23, 164.97, 153.86, 147.04, 142.89, 134.81, 133.39, 132.46, 129.32 (2C), 129.04, 125.21, 116.13, 114.59, 113.52, 65.50, 30.81, 25.55.

#### 3.2.5. Synthesis of N′-(2,4-Dimethoxybenzylidene)-2-((2-Oxo-1,2,3,4-Tetrahydroquinolin-6-Yl)Oxy) Acetohydrazide (**4c**)

Starting from 6-hydroxy-3,4-dihydroquinolin-2*(1H)*-one, compound 4c was prepared through 3 steps, **i**, **ii** and **iii**, respectively. A total of 200 mg of compound **3** (0.8 mmol) was refluxed with 0.16 mL of 2,4-dimethoxybenzaldehyde (1 mmol) in ethanol and drops of acetic acid. After filtration, a pure white powder product was obtained. m.p: 243–245 °C, LC-MS: *m*/*z* 383.97 [M + H]^+^. **^1^H-NMR** (500 MHz, DMSO-*d*_6_) δ 11.39 (* 11.36, s, 1H. C-**NH**-N=), 9.95 (* 9.91, s, 1H. C-N**H**-C), 8.58 (* 8.25, s, 1H. N=C**H**-) 7.77–6.59 (m, 6H**_arom_**), 5.02 (s, 1H. O-**CH_2_**-C=O), 4.55 (s, 1H. O-**CH_2_**-C=O), 3.85 (s, 3H. –O-C**H_3_**), 3.81 (s, 3H. –O-C**H_3_**), 2.86–2.81 (m, 2H. C**H_2_**CH_2_-C=O), 2.43–2.38 (m, 2H. C**H_2_**C=O). (* refer to the rotameric peak). **^13^C-NMR** (125 MHz, DMSO) δ 170.22, 164.39, 162.74, 159.45, 153.93, 143.91 (-N=**C**H-), 139.95, 132.41, 127.16, 125.20, 116.13, 114.56, 113.51, 106.91, 98.56, 65.54, 56.23 (-O-**C**H_3_), 55.90 (-O-**C**H_3_), 30.81, 25.55.

#### 3.2.6. Synthesis 2-((2-Oxo-1,2,3,4-Tetrahydroquinolin-6-Yl)Oxy)-N′-(Thiophen-2-Ylmethylene)Acetohydrazide (**4d**)

A total of 161 mg of compound **3** (0.684 mmol) was heated with 134.45 mg of thiophene-2-carbaldehyde (0.821 mmol) in ethanol under reflux with the presence of AcOH (3 drops). The desired molecule was precipitated when the reaction finished (controlled by TLC) and the solvent was evaporated, and the obtained compound was washed by ethanol to obtain 200 mg (88.7%) as white powder. m.p: 223–225 °C, LC-MS: *m*/*z* 329.97 [M + H]^+^. **^1^H-NMR** (500 MHz, Chloroform-*d*) δ 11.54 (* 11.48, s, 1H. C-**NH**-N=), 9.95 (* 9.92, s, 1H. C-N**H**-C), 8.55 (* 8.18, s, 1H. N=C**H**-) 7.67–6.70 (m, 6H_arom_), 4.97 (s, 1H. O-**CH_2_**-C=O), 4.57 (s, 1H. O-**CH_2_**-C=O), 2.85–2.80 (m, 2H), 2.42–2.38 (m, 2H). (* refer to the rotameric peak). **^13^C-NMR** (125 MHz, Chloroform) δ 169.78, 164.26, 152.88, 143.06, 138.90, 132.48, 131.15, 129.10, 127.88, 124.87, 115.75, 114.39, 113.33, 66.98, 30.30, 25.10.

#### 3.2.7. Synthesis (E)-N′-(Furan-2-Ylmethylene)-2-((2-Oxo-1,2,3,4-Tetrahydroquinolin-6-Yl)Oxy)Acetohydrazide (**4e**)

Starting from 6-hydroxy-3,4-dihydroquinolin-2*(1H)*-one, compound **4e** was prepared through 3 steps, **i**, **ii**, and **iii**, respectively. A total of 210 mg of compound 3 (0.892 mmol) was refluxed with 0.89 mL of furan-2-carbaldehyde (1.07 mmol, d = 1.16 g/mL) in ethanol and drops of acetic acid. After filtration, a pure white powder product was obtained. m.p: 217–218 °C, LC-MS: *m*/*z* 314.04 [M + H]^+^. **^1^H-NMR** (500 MHz, DMSO-*d*) δ 11.50 (* 11.47, s, 1H. C-**NH**-N=), 9.95 (* 9.91, s, 1H. C-N**H**-C), 8.24 (* 7.88, s, 1H. N=C**H**-) 7.84–6.62 (m, 6H_arom_), 4.98 (s, 1H. O-**CH_2_**-C=O), 4.58 (s, 1H. O-**CH_2_**-C=O), 2.85–2.82 (m, 2H. O=C-CH_2_-C**H_2_**-), 2.42–2.38 (m, 2H. O=C-CH_2_-C**H_2_**-). (* refer to the rotameric peak). **^13^C-NMR** (125 MHz, DMSO) δ 169.79, 164.37, 153.37, 148.97, 145.05, 137.72, 133.91, 132.02, 124.78, 115.69, 113.79, 113.06, 112.16, 64.88, 30.35, 25.10.

#### 3.2.8. Synthesis (E)-N′-(4-Methoxybenzylidene)-2-((2-Oxo-1,2,3,4-Tetrahydroquinolin-6-Yl)Oxy)Acetohydrazide (**4f**)

A total of 190 mg of compound **3** (0.807 mmol) was heated with 0.118 mL of 4-methoxybenzaldehyde (0.97 mmol, d = 1.12 g/mL) in ethanol under reflux with the presence of AcOH (3 drops). The desired molecule was precipitated when the reaction finished (controlled by TLC). After workup, 217 mg (76%) of **4f** was obtained as yellowish white powder. m.p: 203–205 °C, LC-MS: *m*/*z* 354.22 [M + H]^+^. **^1^H-NMR** (500 MHz, DMSO-*d*) δ 11.44 (* 11.39, s, 1H. C-**NH**-N=), 9.96 (* 9.92, s, 1H. C-N**H**-C), 8.28 (* 7.95, s, 1H. N=C**H**-) 7.65–6.72 (m, 7H_arom_), 5.05 (s, 1H. O-**CH_2_**-C=O), 4.58 (s, 1H. O-**CH_2_**-C=O), 3.80 (s, 3H. -O-C**H_3_**) 2.86–2.81 (m, 2H. O=C-CH_2_-C**H_2_**-), 2.43–2.38 (m, 2H. O=C-CH_2_-C**H_2_**-). (* refer to the rotameric peak). **^13^C-NMR** (125 MHz, DMSO) δ 169.78 (O=**C**<), 164.12 (O=**C**-NH=N-), 160.69, 153.46, 143.61 (N=**C**H-), 131.98, 128.51, 126.57, 124.76, 115.69, 114.34, 114.28, 114.11, 113.30, 113.05, 65.04 (O-**C**H_2_-C=O), 55.31 (-**C**H_3_), 30.36, 25.10.

#### 3.2.9. Synthesis of (E)-N′-((2-Methyl-1H-Indol-3-Yl)Methylene)-2-((2-Oxo-1,2,3,4-Tetrahydroquinolin-6-Yl)Oxy)Acetohydrazide (**4g**)

Starting from 2-((2-oxo-1,2,3,4-tetrahydroquinolin-6-yl)oxy)acetohydrazide (compound **3**), 4g was prepared through 3 steps, respectively following the mentioned general procedure. A total of 200 mg of compound **3** (0.8 mmol) was refluxed with 162.41 mg of 2-Methylindole-3-carboxaldehyde (1 mmol) in ethanol and drops of acetic acid. After workup and recrystallisation by EtOH, 110 mg (34.5%) was obtained as white powder. m.p: 265–267 °C, LC-MS: *m*/*z* 377.12 [M + H]^+^. **^1^H-NMR** (500 MHz, DMSO-*d*_6_) δ 13.69 (s, 1H. N-**H**), 12.45 (O=C-N**H**-N), 11.25 (s, 1H. N=C**H**-), 9.97 (s, 1H. C-N**H**-), 7.58–6.81 (m, 7 **H_arom_**), 4.78 (s, 2H O-C**H_2_**-), 2.91–2.78 (m, 2H. O=C-CH_2_-C**H_2_**-), 2.50 (s, 3H. –C**H_3_**), 2.41 (t, *J* = 6.9 Hz, 2H. O=C-C**H_2_**-). **^13^C-NMR** (125 MHz, DMSO) δ 169.80 (O=**C**), 162.54 (O=**C**-NH-N=), 152.19, 142.67, 135.57, 131.99, 132.95, 125.06, 122.70, 121.07, 119.71, 115.84 (2C), 114.48, 113.47, 111.22 (2C), 67.45 (O-C**H_2_**-C=O), 30.26, 25.21, 25.06 (-**C**H_3_).

#### 3.2.10. Synthesis of (E)-2-((2-Oxo-1,2,3,4-Tetrahydroquinolin-6-Yl)Oxy)-N′-(4-Propylbenzylidene)Acetohydrazide (**4h**)

Starting from 6-hydroxy-3,4-dihydroquinolin-2*(1H)*-one, **4h** was prepared through 3 steps, i, ii, and iii, respectively. A total of 210 mg of compound **3** (0.893 mmol) was refluxed with 0.158 mL of 4-propylbenzaldehyde (1.07 mmol, d = 1.005 g/mL) in ethanol and drops of acetic acid. After filtration, a pure white powder product was yielded (285 mg, 87.3%). m.p: 189–190 °C, LC-MS: *m*/*z* 366.20 [M + H]^+^. **^1^H-NMR** (500 MHz, DMSO-*d*_6_) δ 11.50 (* 11.45, s, 1H. C-**NH**-N=), 9.96 (* 9.92, s, 1H. C-N**H**-C), 8.31 (* 7.97, s, 1H. N=C**H**-) 7.61–6.73 (m, 7H_arom_), 5.05 (s, 1H. O-**CH_2_**-C=O), 4.58 (s, 1H. O-**CH_2_**-C=O), 2.85–2.81 (m, 2H. O=C-C**H_2_**-), 2.57 (t, *J* = 7.5 Hz, 2H. O=C-CH_2_-C**H_2_**-), 2.43–2.36 (m, 2H. =C-C**H_2_**-CH_2_), 1.62–1.56 (m, 2H. –C**H**_2_-CH_3_), 0.88 (t, *J* = 8.6 Hz, 3H. CH_3_). (* refer to the rotameric peak). **^13^C-NMR** (125 MHz, DMSO) δ 169.76, 164.27, 153.44, 147.96, 144.63, 143.81, 132.00, 131.56, 128.80, 127.12, 126.90, 124.75, 115.68, 114.12, 113.05, 65.05, 37.10, 30.36, 25.09, 23.92, 13.59.

#### 3.2.11. Synthesis of (E)-N′-(Cyclohexylmethylene)-2-((2-Oxo-1,2,3,4-Tetrahydroquinolin-6-Yl)Oxy)Acetohydrazide (**4i**)

Starting from 6-hydroxy-3,4-dihydroquinolin-2*(1H)*-one, compound 2-((2-oxo-1,2,3,4-tetrahydroquinolin-6-yl)oxy)acetohydrazide (compound **3**) was prepared through a couple of steps, following procedures **i** and **ii**, respectively. After that, following procedure **iii**, 180 mg of compound **3** (0.765 mmol) was stirred under reflux with 0.112 mL of cyclohexanecarbaldehyde (0.918 mmol) in ethanol and 3 drops of acetic acid. After workup, by extracting with DCM and water, 200 mg (79.3%) was obtained as white powder. m.p: 161–163 °C, LC-MS: *m*/*z* 329.99 [M + H]^+^. **^1^H-NMR** (500 MHz, DMSO-d6) δ 11.08 (* 11.01, s, 1H. C-**NH**-N=), 9.94 (* 9.90, s, 1H. C-N**H**-C), 7.52 (* 7.21, s, 1H. N=C**H**-) 6.82–6.65 (m, 3H_arom_), 4.86 (s, 1H. O-**CH_2_**-C=O), 4.47 (s, 1H. O-**CH_2_**-C=O), 2.84–2.80 (m, 2H. O=C-C**H_2_**-), 2.41–2.37 (m, 2H. O=C-CH_2_-C**H_2_**-), 2.21–2.15 (m, 1H. =CH-C**H**<), 1.76–1.60 (m, 5H_CycloHexane_), 1.31–1.13 (m, 5H_CycloHexane_). (* r efer to the rotameric peak). **^13^C-NMR** (125 MHz, DMSO) δ 169.75, 168.62, 163.85, 155.78, 153.40, 151.61, 131.96, 124.73, 115.66, 114.04, 112.99, 64.89, 30.34, 30.29, 29.60, 29.53, 25.50, 25.08, 24.96, 24.95.

#### 3.2.12. Synthesis of (E)-N′-Benzylidene-2-((2-Oxo-1,2,3,4-Tetrahydroquinolin-6-Yl)Oxy)Acetohydrazide (**4j**)

Starting from 6-hydroxy-3,4-dihydroquinolin-2*(1H)*-one, compound 2-((2-oxo-1,2,3,4-tetrahydroquinolin-6-yl)oxy)acetohydrazide (compound **3**) was prepared through a couple of steps, following procedures **i** and **ii**, respectively. After that, following procedure **iii**, 200 mg of compound **3** (0.850 mmol) was refluxed with 95 μL of benzaldehyde (0.935 mmol) in ethanol and drops of acetic acid for 6 h. After completing the reaction, the precipitate was filtered out of the hot ethanol, washed with hot ethanol, and dried to obtain 220 mg (80%) of the final product as white powder. m.p: 161–163 °C, LC-MS: *m*/*z* 324.11 [M + H]^+^. **^1^H-NMR** (500 MHz, DMSO-*d*_6_) δ 11.56 (* 11.53, s, 1H. C-**NH**-N=), 9.96 (* 9.92, s, 1H. C-N**H**-C), 8.34 (* 8.01, s, 1H. N=C**H**-) 7.70–6.74 (m, 8H_arom_), 5.07 (s, 1H. O-**CH_2_**-C=O), 4.60 (s, 1H. O-**CH_2_**-C=O), 2.86–2.81 (m, 2H. O=C-CH_2_-C**H_2_**-), 2.43–2.38 (m, 2H. O=C-CH_2_-C**H_2_**-). (* refer to the rotameric peak). **^13^C-NMR** (125 MHz, DMSO) δ 170.26 (O=**C**<), 164.89 (O=**C**-NH=N-), 153.88, 148.39, 144.21 (N=**C**H-), 134.41, 132.44, 130.41, 129.30, 127.37 (2C), 125.23, 116.15, 114.59, 113.51, 65.51, 30.80, 25.54.

#### 3.2.13. Synthesis of (Z)-2-((2-Oxo-1,2,3,4-Tetrahydroquinolin-6-Yl)Oxy)-N′-(2-Oxoindolin-3-Ylidene)Acetohydrazide (**4k**)

Starting from hydroxy-3,4-dihydroquinolin-2*(1H)*-one, **4k** was prepared through three steps, following procedures **i**, **ii**, and **iii**, respectively. In the first and second steps, compounds **2** and **3** were obtained, as previously mentioned. In the third step, 250 mg of 2-((2-oxo-1,2,3,4-tetrahydroquinolin-6-yl)oxy)acetohydrazide (compound **3**) (1.06 mmol) was refluxed with 156 mg of isatin (1.06 mmol) in ethanol and 3 drops of acetic acid for 5 h. After completing the reaction, the product was filtered out the hot ethanol to obtain 335 mg of the final product as yellow powder with a yield of 86%. m.p: >300 °C, LC-MS: *m*/*z* 363.34 [M − H]^−^. **^1^H-NMR** (500 MHz, DMSO) δ 13.69 (s, 1H .O=C-N**H**-), 11.25 (s, 1H .C=N-N**H**-), 9.97 (s, 1H.N=N=C**H**-), 7.58–6.81 (m, 6**H**_arom._), 4.78 (s, 2H. O-C**H_2_**-), 2.89–2.84 (m, 2H. O=C-C**H_2_**-), 2.41 (t, *J* = 3 Hz, 2H. O=CH2-C**H_2_**-). **^13^C-NMR** (125 MHz, DMSO) δ 169.80, 162.54, 152.19, 142.67, 135.57, 131.99, 125.06, 122.70, 121.07, 119.71, 115.84 (2C), 114.48, 113.47, 111.22 (2C), 67.45, 30.26, 25.06.

#### 3.2.14. Synthesis of (Z)-N′-(5-Fluoro-1-Methyl-2-Oxoindolin-3-Ylidene)-2-((2-Oxo-1,2,3,4-Tetrahydroquinolin-6-Yl)Oxy)Acetohydrazide (**4l**)

Starting from 6-hydroxy-3,4-dihydroquinolin-2*(1H)*-one, compound 2-((2-oxo-1,2,3,4-tetrahydroquinolin-6-yl)oxy)acetohydrazide (compound **3**) was prepared through a couple of steps, following procedures **i** and **ii**, respectively. Next, 5-fluoro-1-methyl-2-indolin-2,3-dione was prepared according to procedure **i**, by reacting 5-fluoruisatin with methyl iodide under the basic condition of K_2_CO_3_ at room temperature_._ After that, following procedure (**ii**), 200 mg of compound **3** (0.850 mmol) was refluxed with 152 mg of 5-fluoro-1-methyl-2-indolin-2,3-dione (0.850 mmol) in ethanol and drops of acetic acid for 10 h. After completing the reaction, the precipitate was filtered out of the hot ethanol, washed with hot ethanol, and dried to obtain 275 mg (81%) of the final product as white powder. m.p: >300 °C, LC-MS: *m*/*z* 397.12 [M + H]^+^. **^1^H-NMR** (500 MHz, DMSO-d6) δ 13.74 (s, 1H .O=C-N**H**-), 11.35 (s, 1H .C=N-N**H**-), 7.51–6.78 (m, 6**H**_arom._), 4.80 (s, 2H. O-C**H_2_**-), 3.38 (s, 3H. -CH_3_), 2.89–2.86 (m, 2H. O=C-C**H_2_**-), 2.48 (t, *J* = 3 Hz, 2H. O=CH_2_-C**H_2_**-). **^13^C-NMR** (125 MHz, DMSO) δ 170.24, 163.10, 159.74, 157.85, 139.35, 125.46, 121.46, 121.39, 118.64, 116.28, 114.88, 113.89, 112.81, 112.74, 108.72, 108.50, 56.49, 30.72, 27.48, 25.51.

#### 3.2.15. Synthesis of (Z)-N′-(5-Fluoro-2-Oxoindolin-3-Ylidene)-2-((2-Oxo-1,2,3,4-Tetrahydroquinolin-6-Yl)Oxy)Acetohydrazide (**4m**)

Starting from hydroxy-3,4-dihydroquinolin-2*(1H)*-one, **4m** was prepared through three steps, following procedure **i**, **ii**, and **iii**, respectively. In the first and second steps, compounds **2** and **3** were obtained as previously mentioned. In the third step, 250 mg of 2-((2-oxo-1,2,3,4-tetrahydroquinolin-6-yl)oxy)acetohydrazide, compound **3** (1.06 mmol), was refluxed with 175 mg of 5-flouroisatin (1.06 mmol) in ethanol and 3 drops of acetic acid for 9 h. After completing the reaction, the product was filtered out the hot ethanol to obtain 321 mg of the final product as yellow powder with a yield of 79%. m.p: >300 °C, LC-MS: *m*/*z* 381.10 [M − H]^−^. **^1^H-NMR** (500 MHz, DMSO-d6) δ 13.70 (s, 1H. O=C-N**H**-), 11.28 (s, 1H .C=N-N**H**-), 9.97 (s, 1H. O=C-N**H_Indol_**), 7.40–6.81 (m, 6**H**_arom._), 4.80 (s, 2H. O-C**H_2_**-), 2.87–2.84 (m, 2H. O=C-C**H_2_**-), 2.41 (t, *J* = 3 Hz, 2H. O=CH_2_-C**H_2_**-). **^13^C-NMR** (125 MHz, DMSO) δ 170.24, 163.10, 159.74, 157.85, 139.35, 125.46, 121.46, 121.39, 118.64, 116.28, 114.88, 113.89, 112.81, 112.74, 108.72, 108.50, 56.49, 30.72, 25.51.

#### 3.2.16. Synthesis of (E)-N′-(4-Nitrobenzylidene)-2-((2-Oxo-1,2,3,4-Tetrahydroquinolin-6-Yl)Oxy)Acetohydrazide (**4n**)

Starting from 6-hydroxy-3,4-dihydroquinolin-2*(1H)*-one, compound 2-((2-oxo-1,2,3,4-tetrahydroquinolin-6-yl)oxy)acetohydrazide (compound **3**) was prepared through a couple of steps, following procedures **i** and **ii**, respectively. After that, following procedure **iii**, 200 mg of compound **3** (0.850 mmol) was refluxed with 151 mg of 4-nitrobenzaldehyde (0.850 mmol) in ethanol and drops of acetic acid for 6 h. After completing the reaction, the precipitate was filtered out of the hot ethanol, washed with hot ethanol, and dried to obtain 271 mg (86%) of the final product as white powder. m.p: 249–251 °C, LC-MS: *m*/*z* 367.21 [M−H]^−^. **^1^H-NMR** (500 MHz, DMSO-*d*_6_) δ 11.86 (* 11.84, s, 1H. C-**NH**-N=), 9.97 (* 9.93, s, 1H. C-N**H**-C), 8.45 (* 8.25, s, 1H. N=C**H**-) 8.30–8.25 (m, 2H**_arom_**), 7.98–6.77 (m, 5H**_arom_**), 5.12 (s, 1H. O-**CH_2_**-C=O), 4.65 (s, 1H. O-**CH_2_**-C=O), 2.85–2.82 (m, 2H. O=C-CH_2_-C**H_2_**-), 2.42–2.39 (m, 2H. O=C-CH_2_-C**H_2_**-). (* refer to the rotameric peak). **^13^C-NMR** (125 MHz, DMSO) δ 170.23 (O=**C**<), 165.38 (O=**C**-NH=N-), 153.81, 148.17, 145.89, 141.80, 140.72, 132.51, 128.32 (2C), 124.43 (2C), 116.14, 114.61, 113.52, 65.52 (O-**C**H_2_-C=O), 30.80, 25.54.

#### 3.2.17. Synthesis of (Z)-N′-(5-Nitro-2-Oxoindolin-3-Ylidene)-2-((2-Oxo-1,2,3,4-Tetrahydroquinolin-6-Yl)Oxy)Acetohydrazide (**4o**)

Starting from hydroxy-3,4-dihydroquinolin-2*(1H)*-one, TR-G056 was prepared through three steps, following procedures **i**, **ii**, and iii, respectively. In the first and second steps, compounds **2** and **3** were obtained as previously mentioned. In the third step, 250 mg of 2-((2-oxo-1,2,3,4-tetrahydroquinolin-6-yl)oxy)acetohydrazide, compound **3** (1.06 mmol), was refluxed with 204 mg of 5-nitroisatin (1.06 mmol) in ethanol and 3 drops of acetic acid for 9 h. After completing the reaction, the product was filtered out of the hot ethanol to obtain 387 mg of the final product as yellow powder with a yield of 86%. m.p: 95–97 °C, LC-MS: *m*/*z* 408.26 [M−H]^−^. **^1^H-NMR** (500 MHz, DMSO-d6) δ 13.40 (s, 1H. O=C-N**H**-), 11.30 (s, 1H. C=N-N**H**-), 10.02 (s, 1H. O=C-N**H_Indol_**), 7.43–6.84 (m, 6**H**_arom._), 4.81 (s, 2H. O-C**H_2_**-), 2.88–2.86 (m, 2H. O=C-C**H_2_**-), 2.42 (t, *J* = 3 Hz, 2H. O=CH_2_-C**H_2_**-). **^13^C-NMR** (125 MHz, DMSO) δ 172.61, 163.14, 160.03, 158.03, 140.39, 126.49, 121.46, 121.41, 118.68, 117.23, 117.88, 115.89, 114.81, 113.74, 109.72, 109.50, 57.49, 29.72, 26.51.

#### 3.2.18. Synthesis of (E)-2-((2-Oxo-1,2,3,4-Tetrahydroquinolin-6-Yl)Oxy)-N′-(Pyridin-2-Ylmethylene)Acetohydrazide (**4p**)

Starting from 6-hydroxy-3,4-dihydroquinolin-2*(1H)*-one, compound 2-((2-oxo-1,2,3,4-tetrahydroquinolin-6-yl)oxy)acetohydrazide (compound **3**) was prepared through a couple of steps, following procedures **i** and **ii**, respectively. After that, following procedure **iii**, 200 mg of compound **3** (0.850 mmol) was refluxed with 88 μL of 2-pyridinecarboxaldehyde (0.935 mmol) in ethanol and drops of acetic acid for 6 h. After completing the reaction, the precipitate was filtered out of the hot ethanol, washed with hot ethanol, and dried to obtain 220 mg (80%) of the final product as beige powder. m.p: 134–136 °C, LC-MS: *m*/*z* 325.2 [M + H]^+^. **^1^H-NMR** (500 MHz, DMSO-*d*_6_) δ 11.84 (s, 1H. C-**NH**-N=), 9.97 (* 9.93, s, 1H. C-N**H**-C), 8.59 (* 8.37, s, 1H. N=C**H**-) 8.05–6.74 (m, 7H_arom_), 5.09 (s, 1H. O-**CH_2_**-C=O), 4.64 (s, 1H. O-**CH_2_**-C=O), 2.84–2.81 (m, 2H. O=C-CH_2_-C**H_2_**-), 2.41–2.38 (m, 2H. O=C-CH_2_-C**H_2_**-). (* refer to the rotameric peak). **^13^C-NMR** (125 MHz, DMSO) δ 169.91, 153.44, 152.88, 149.52, 144.25, 136.88, 132.07, 124.85, 124.40, 119.91, 115.77, 114.46, 114.20, 113.13, 65.08, 30.34, 25.13.

#### 3.2.19. Synthesis of (E)-N′-((2-(4-Chlorophenyl)Pyrimidin-5-Yl)Methylene)-2-((2-Oxo-1,2,3,4-Tetrahydroquinolin-6-Yl)Oxy)Acetohydrazide (**4q**)

Starting from 6-hydroxy-3,4-dihydroquinolin-2*(1H)*-one, **4q** was prepared through 3 steps, following procedures **i**, **ii**, and **iii**, respectively. Starting from 6-hydroxy-3,4-dihydroquinolin-2(1*H*)-one, firstly, 300 mg of 6-hydroxy-3,4-dihydroquinolin-2*(1H)*-one (1.84 mmol) was stirred with 381 mg of the base K_2_CO_3_ (2.76 mmol) for 15 min in THF and drops of DMF, then 244 μL of ethyl 2-bromoacetate (2.21 mmol, d = 1.51 g/mL) was added slowly to the reaction mixture. After the completion of the reaction, the solvent was evaporated and the residue was washed with water, filtered, and air-dried to obtain ethyl 2-((2-oxo-1,2,3,4-tetrahydroquinolin-6-yl)oxy)acetate (compound **2**), which was further refluxed with 156 μL hydrazine hydrate 99% (4.81 mmol) for 5 h, then cooled to room temperature, filtered out of ethanol, and dried to obtain 2-((2-oxo-1,2,3,4-tetrahydroquinolin-6-yl)oxy)acetohydrazide. Later, 200 mg of 2-((2-oxo-1,2,3,4-tetrahydroquinolin-6-yl)oxy)acetohydrazide (compound **3**) (0.850 mmol) was refluxed with 278.83 mg of 2-(4-chlorophenyl)pyrimidine-5-carbaldehyde (1.28 mmol) in ethanol and drops of acetic acid for 6 h. After filtration, the product was obtained as pure white powder. m.p: 259–261 °C, LC-MS: *m*/*z* 436.81 [M + H]^+^. **^1^H-NMR** (500 MHz, DMSO-*d*_6_) δ 11.96 (* 11.88, s, 1H. C-**NH**-N=), 9.96 (* 9.92, s, 1H. C-N**H**-C), 9.20–9.13 (m, 2H_pyrimidine_), 8.42–8.39 (m, 2H_arom_), 8.07 (* 8.04, s, 1H. N=C**H**-) 7.60–6.74 (m, 5H_arom_), 5.07 (s, 1H. O-**CH_2_**-C=O), 4.60 (s, 1H. O-**CH_2_**-C=O), 2.86–2.81 (m, 2H. O=C-CH_2_-C**H_2_**-), 2.43–2.38 (m, 2H. O=C-CH_2_-C**H_2_**-). (* refer to the rotameric peak). **^13^C-NMR** (125 MHz, DMSO) δ 169.77, 169.58, 162.18, 155.66, 153.38, 152.87, 142.62, 138.08, 136.12, 135.52, 132.51, 132.02, 129.56, 128.96, 126.30, 124.76, 115.68, 114.15, 113.11, 65.10, 30.36, 25.10.

#### 3.2.20. Synthesis of (E)-N′-((2-Aminopyrimidin-5-Yl)Methylene)-2-((2-Oxo-1,2,3,4-Tetrahydroquinolin-6-Yl)Oxy)Acetohydrazide (**4r**)

Starting from 6-hydroxy-3,4-dihydroquinolin-2*(1H)*-one, compound 2-((2-oxo-1,2,3,4-tetrahydroquinolin-6-yl)oxy)acetohydrazide (compound **3**) was prepared through a couple of steps, following procedures **i** and **ii**, respectively. After that, following procedure **iii**, 200 mg of compound **3** (0.850 mmol) was refluxed with 105 mg of 2-pyridinecarboxaldehyde (0.850 mmol) in ethanol and drops of acetic acid for 6 h. After completing the reaction, the precipitate was filtered out of the hot ethanol, washed with hot ethanol, and dried to obtain 231 mg (79%) of the final product as white powder. m.p: 284–286 °C, LC-MS: *m*/*z* 339.21 [M−H]^−^. **^1^H-NMR** (500 MHz, DMSO-*d*) δ 11.42 (* 11,40, s, 1H. C-**NH**-N=), 9.95 (* 9.90, s, 1H. C-N**H**-C), 8.55 (s, 1H. -N**H**_2_), 8.51 (s, 1H. -N**H**_2_), 8.16 (* 7.82, s, 1H. N=C**H**-), 7.17–6.71 (m, 5H**_arom_**), 5.02 (s, 1H. O-**CH_2_**-C=O), 4.55 (s, 1H. O-**CH_2_**-C=O), 2.84–2.81 (m, 2H. O=C-CH_2_-C**H_2_**-), 2.42–2.37 (m, 2H. O=C-CH_2_-C**H_2_**-). (* refer to the rotameric peak). **^13^C-NMR** (125 MHz, DMSO) δ 169.79, 163.70, 156.98, 153.46, 144.36, 139.97, 132.44, 131.94, 124.87, 116.91, 115.67, 114.11, 113.08, 65.05, 30.31, 25.10.

#### 3.2.21. Synthesis of (E)-N′-(4-Fluorobenzylidene)-2-((2-Oxo-1,2,3,4-Tetrahydroquinolin-6-Yl)Oxy)Acetohydrazide (**4s**)

Starting from 6-hydroxy-3,4-dihydroquinolin-2*(1H)*-one, compound 2-((2-oxo-1,2,3,4-tetrahydroquinolin-6-yl)oxy)acetohydrazide (compound **3**) was prepared through a couple of steps, following procedures **i** and **ii**, respectively. After that, following procedure **iii**, 200 mg of compound **3** (0.850 mmol) was refluxed with 100 μL of 4-flourobenzaldehyde (0.935 mmol) in ethanol and drops of acetic acid for 6 h. After completing the reaction, the precipitate was filtered out of the hot ethanol, washed with hot ethanol, and dried to obtain 250 mg (86%) of the final product as white powder. m.p: 238–240 °C, LC-MS: *m*/*z* 342.22 [M + H]^+^. **^1^H-NMR** (500 MHz, DMSO-d6) δ 11.58 (* 11.54, s, 1H. C-**NH**-N=), 9.97 (* 9.93, s, 1H. C-N**H**-C), 8.35 (* 8.01, s, 1H. N=C**H**-) 7.79–6.73 (m, 7H_arom_), 5.08 (s, 1H. O-**CH_2_**-C=O), 4.61 (s, 1H. O-**CH_2_**-C=O), 2.87–2.82 (m, 2H. O=C-CH_2_-C**H_2_**-), 2.43–2.39 (m, 2H. O=C-CH_2_-C**H_2_**-). (* refer to the rotameric peak). **^13^C-NMR** (125 MHz, DMSO) δ 169.82, 164.47, 153.45, 146.84, 142.61, 132.49, 132.02, 129.37, 129.10, 124.79, 115.95, 115.78, 114.41, 114.15, 113.08, 65.07, 30.38, 25.12.

#### 3.2.22. Synthesis of (E)-N′-(3-(4-Chlorophenoxy)Benzylidene)-2-((2-Oxo-1,2,3,4-Tetrahydroquinolin-6-Yl)Oxy)Acetohydrazide (**4t**)

Starting from 6-hydroxy-3,4-dihydroquinolin-2(1*H*)-one, compound 2-((2-oxo-1,2,3,4-tetrahydroquinolin-6-yl)oxy)acetohydrazide (compound **3**) was prepared through a couple of steps, following procedures **i** and **ii**, respectively. After that, following procedure **iii**, 200 mg of compound **3** (0.850 mmol) was refluxed with 198 mg of 3-(4-chlorophenoxy)benzaldehyde (0.850 mmol) in ethanol and drops of acetic acid for 6 h. After completing the reaction, the precipitate was filtered out of the hot ethanol, washed with hot ethanol, and dried to obtain 320 mg (83%) of the final product. m.p: 205–207 °C, LC-MS: *m*/*z* 450.92 [M + H]^+^. **^1^H-NMR** (500 MHz, DMSO-*d*) δ 11.61 (* 11.56, s, 1H. C-**NH**-N=), 9.96 (* 9.92, s, 1H. C-N**H**-C), 8.32 (* 7.98, s, 1H. N=C**H**-) 7.49–6.70 (m, 11H**_arom_**), 5.03 (s, 1H. O-**CH_2_**-C=O), 4.59 (s, 1H. O-**CH_2_**-C=O), 2.83–2.80 (m, 2H. O=C-CH_2_-C**H_2_**-), 2.41–2.37 (m, 2H. O=C-CH_2_-C**H_2_**-). (* refer to the rotameric peak). **^13^C-NMR** (125 MHz, DMSO) δ 169.77, 164.52, 156.57, 155.44, 153.39, 152.88, 147.08, 142.87, 136.19, 132.47, 132.00, 130.71, 129.94, 127.39, 124.74, 122.72, 120.28, 116.74, 115.74, 114.13, 113.03, 65.03, 30.36, 25.10.

#### 3.2.23. Synthesis of (E)-N′-(Naphthalen-1-Ylmethylene)-2-((2-Oxo-1,2,3,4-Tetrahydroquinolin-6-Yl)Oxy)Acetohydrazide (**4u**)

Starting from 6-hydroxy-3,4-dihydroquinolin-2*(1H)*-one, compound 2-((2-oxo-1,2,3,4-tetrahydroquinolin-6-yl)oxy)acetohydrazide (compound **3**) was prepared through a couple of steps, following procedures **i** and **ii**, respectively. After that, following procedure **iii**, 200 mg of compound **3** (0.850 mmol) was refluxed with 125 μL of 1-naphthaldehyde (0.935 mmol) in ethanol and drops of acetic acid for 6 h. After completing the reaction, the precipitate was filtered out of the hot ethanol, washed with hot ethanol, and dried to obtain 231 mg (72%) of the final product as white powder. m.p: 259–261 °C, LC-MS: *m*/*z* 374.28 [M + H]^+^. **^1^H-NMR** (500 MHz, DMSO-*d*) δ 11.63 (* 11.59, s, 1H. C-**NH**-N=), 9.97 (* 9.93, s, 1H. C-N**H**-C), 9.02 (* 8.68, s, 1H. N=C**H**-) 8.81–6.75 (m, 10H**_arom_**), 5.15 (s, 1H. O-**CH_2_**-C=O), 4.66 (s, 1H. O-**CH_2_**-C=O), 2.87–2.82 (m, 2H. O=C-CH_2_-C**H_2_**-), 2.43–2.38 (m, 2H. O=C-CH_2_-C**H_2_**-). (* refer to the rotameric peak). **^13^C-NMR** (125 MHz, DMSO) δ 169.78, 164.46, 153.44, 147.88, 143.50, 133.50, 132.02, 130.43, 130.05, 129.22, 128.85, 127.39, 126.30, 125.56, 124.78, 123.75, 115.72, 114.49, 113.07, 65.24, 54.94, 30.31, 25.11.

#### 3.2.24. Synthesis of (E)-N′-([1,1′-Biphenyl]-4-Ylmethylene)-2-((2-Oxo-1,2,3,4-Tetrahydroquinolin-6-Yl)Oxy)Acetohydrazide (**4v**)

According to procedure **i**, **ii**, and **iii**, respectively, starting from 6-hydroxy-3,4-dihydroquinolin-2(1*H*)-one, 300 mg of 6-hydroxy-3,4-dihydroquinolin-2*(1H)*-one (1.84 mmol) was stirred with 381 mg of the base K_2_CO_3_ (2.76 mmol) for 15 min in THF and drops of DMF, then 244 μL of ethyl 2-bromoacetate (2.21 mmol, d = 1.51 g/mL) was added slowly to the reaction mixture. After the completion of the reaction, the solvent was evaporated and the residue was washed with water, filtered, and air-dried to obtain ethyl 2-((2-oxo-1,2,3,4-tetrahydroquinolin-6-yl)oxy)acetate (compound **2**), which was further refluxed with 241 μL hydrazine hydrate 99% (4.81 mmol) for 5 h, then cooled to room temperature, filtered out of ethanol, and dried to obtain 2-((2-oxo-1,2,3,4-tetrahydroquinolin-6-yl)oxy)acetohydrazide (compound **3**). Later, 210 mg of compound **3** (0.892 mmol) was refluxed with 211.47 mg of [1,1′-biphenyl]-4-carbaldehyde (1.16 mmol) in ethanol and drops of acetic acid for 6 h. After that, the precipitate was filtered out of ethanol to obtain 283 mg of the final product, as white powder with a yield of 79%. m.p: 224–226 °C, LC-MS: *m*/*z* 400.59 [M + H]^+^. **^1^H-NMR** (500 MHz, DMSO-*d*) δ 11.59 (s, 1H .C-**NH**-N=), 9.97 (* 9.93, s, 1H. C-N**H**-C), 8.39 (* 8.05, s, 1H. N=C**H**-) 7.79–6.76 (m, 12H**_arom_**), 5.10 (s, 1H. O-**CH_2_**-C=O), 4.61 (s, 1H. O-**CH_2_**-C=O), 2.84–2.81 (m, 2H. O=C-CH_2_-C**H_2_**-), 2.42–2.38 (m, 2H. O=C-CH_2_-C**H_2_**-). (* refer to the rotameric peak). **^13^C-NMR** (125 MHz, DMSO) δ 169.79 (O=**C**<), 164.43 (O=**C**-NH=N-), 153.45, 147.49, 143.35, 141.42, 139.32, 133.09, 132.48, 132.02, 129.04 (2C), 127.75, 127.06, 126.68 (2C), 124.78, 115.77, 115.71, 114.15, 113.08, 65.09, 30.31, 25.11.

#### 3.2.25. Synthesis of (E)-N′-((4′-Chloro-[1,1′-Biphenyl]-4-Yl)Methylene)-2-((2-Oxo-1,2,3,4-Tetrahydroquinolin-6-Yl)Oxy)Acetohydrazide (**4w**)

Starting from 6-hydroxy-3,4-dihydroquinolin-2*(1H)*-one, **4w** was prepared through 3 steps following procedures **i**, **ii**, and **iii**, respectively. A total of 200 mg of compound **3** (0.850 mmol) was refluxed with 221 mg of 4′-chloro-[1,1′-biphenyl]-4-carbaldehyde (1.02 mmol) in ethanol and drops of acetic acid. After filtration, the product was obtained as pure off-white powder with a yield of 84% (310 mg). m.p: 279–281 °C, LC-MS: *m*/*z* 434.97 [M + H]^+^. **^1^H-NMR** (500 MHz, DMSO-*d*) δ 11.61 (s, 1H. C-**NH**-N=), 9.96 (* 9.92, s, 1H. C-N**H**-C), 8.38 (* 8.04, s, 1H. N=C**H**-) 7.79–6.73 (m, 11H_arom_), 5.09 (s, 1H. O-**CH_2_**-C=O), 4.61 (s, 1H. O-**CH_2_**-C=O), 2.86–2.81 (m, 2H. O=C-CH_2_-C**H_2_**-), 2.42–2.38 (m, 2H. O=C-CH_2_-C**H_2_**-). (* refer to the rotameric peak). **^13^C-NMR** (125 MHz, DMSO) δ 169.77 (O=**C**<), 164.45 (O=**C**-NH=N-), 153.43, 147.33, 143.20, 140.00, 138.11, 133.43, 132.75, 132.47, 132.01, 128.98, 128.45, 128.44 (2C), 127.58, 126.96, 124.76, 115.69, 114.14, 113.07, 65.08 (O-**C**H_2_-C=O), 30.36, 25.10.

### 3.3. Molecular Docking

To understand the molecular interaction mechanisms between the target proteins and the suitably repositioned therapeutic candidates, molecular docking investigations were carried out. The protein data bank’s 3D coordinates for the VEGFR2 kinase domain protein were obtained (PDB ID: 2XIR). A computational docking study was performed using the virtual screening tool PyRx. This software uses Lamarck’s Genetic Algorithm (LGA) as a scoring function in AutoDock Vina. The PyRx-virtual screening tool was used in this investigation to perform molecular docking interactions via the AutoDock VINA module [45]. Schrödinger Maestro and Pymol software were used to get and visualise the docked compounds with better binding affinity (kcal/mol) results. The protein structure was prepared, and only polar hydrogens were added, using the AutoDock Tools 1.5.6 module in PyRx tool, and the pdbqt files of proteins were generated. ChemBioDraw Ultra 14.0 was used to build each compound, charged and minimised by the MMFF94x force field, along with the adjustment of hydrogens and lone pairs [46]. The compounds were saved in the SDF format. The target protein active site pocket was docked with the synthetic derivatives and reference molecules. Based on the interaction profile and docking scores, the best suited docked conformation was chosen.

### 3.4. Molecular Dynamics Simulation

Molecular dynamics (MD) simulations were performed using Desmond via the Schrödinger Maestro interface, 13.8 version. These simulations followed the docking phase and involved an orthorhombic simulation cell filled with TIP3 water model molecules. Chloride ions were added to neutralise the overall charge of the complex. The simulation ran for 100 ns within an NPT ensemble, which maintained a constant number of atoms, pressure at 1.01325 bar, and temperature at 300 K. The Nose–Hoover chain method with a 1.0 ps interval was employed as the thermostat, while the Martyna–Tobias–Klein method with a 2.0 ps interval was used as the barostat. The Maestro simulation interaction diagram was utilised to analyse the MD simulation results.

### 3.5. In Silico ADME Study

A computational analysis of 23 novel analogues **4**(**a**–**w**) was carried out using the Schrödinger QikProp tool for prediction of ADME properties, SwissADME, and the PreADME online server, a computational toolbox, to determine which substances have the best possible pharmacokinetic characteristics or BBB permeability.

### 3.6. In Vitro Experiments on GBM Models

#### 3.6.1. Cell Cultures

U87-MG (HTB-16) and U138-MG (HTB-14) lines were purchased from ATCC. The cells were cultured in DMEM-HG Medium (41966-029, Gibco, Grand Island, NE, USA) and supplemented with 10% fetal bovine serum (FBS, 10500-064, Gibco) and 1% Penicillin–Streptomycin (P/S, 15140-122, Gibco). After 24 h of seeding cells, all compounds dissolved in 0.4% dimethyl sulfoxide (DMSO, A3672-0250, AppliChem, Darmstadt, Germany) were treated in the cells.

#### 3.6.2. MTT Assay

The proliferation effects of the drug candidates on the U87-MG and U138-MG cell lines were tested using the MTT (3-(4,5-dimethylthiazol-2-yl)-2,5-diphenyltetrazolium bromide) assay. A total of 7 × 10^3^ cells per well of both U87-MG and U138-MG cells were seeded into a 96-well plate in triplicate for the assay. After 24 h, each compound was added at a dose of 10 μM and incubated for 48 h. After the incubation period, 10 μL of MTT solution (5 mg/mL in PBS) was added to each well. The MTT solution was aspirated from the wells after 2 h. Then, 100 μL of DMSO was added and mixed to dissolve the formazan crystals. Cell proliferation was analysed by measuring the absorbance of the dissolved formazan at a wavelength of 570 nm using a microplate reader (Epoch, San Jose, CA, USA). IC50 values were later calculated using eight drug concentrations (0, 1.25, 2.5, 5, 10, 20, 40, 80, and 160 μM). Each experiment was carried out in triplicate to ensure reliability and statistical significance.

#### 3.6.3. PAMPA Assay

The PAMPA assay (BioAssay System, Hayward, CA 94545, USA) has been used for testing the permeability rates of drugs through the BBB-mimicking membrane. The permeability assay and analyses were performed according to the manufacturer’s instructions. Briefly, high permeability and low permeability standards, compounds (500 μM), and equilibrium standards (200 μM) were prepared. After the loading of lipid solutions into the pores, all compounds’ solutions and the permeability controls were put onto the lipid membrane in donor wells. The permeability assay was performed for 18 h in a 37 °C incubator. All samples were collected from acceptor wells after the incubation period was finished, and the absorbance values were measured with equilibrium standards for normalisation.

## 4. Conclusions

The most frequent malignant primary brain tumour is glioblastoma. Patients with this illness often have a dismal prognosis, and long-term survival is rare.

The common treatment upon initial diagnosis typically includes surgical resection, followed by concurrent radiation therapy and temozolomide treatment. However, biological factors, such as the blood–brain barrier, besides the unique tumour and immune microenvironment, present significant challenges to the development of novel therapeutics compared to other solid tumours. To improve outcomes for glioblastoma patients, a combination of innovative clinical trial designs, targeted drug therapies, and biomarker enrichment approaches is required.

This study focuses on the synthesis of 23 novel analogues of 3,4-dihydroquinolin-2(1H)-one, aiming to offer potential therapeutic solutions targeting the VEGFR2 gene for glioblastoma treatment. The synthesis and evaluation of these analogues were carried out using a deliberate strategy of design and structural modifications to optimise their interactions with the VEGFR2 protein.

This research includes extensive in vitro analyses to assess the therapeutic efficacy of these compounds in two glioblastoma cell lines, U87-MG and U138-MG. The findings demonstrated that all compounds, particularly **4u**, **4t**, **4m**, and **4q**, exhibited strong binding affinity for VEGFR2, with 4m showing the highest binding energy values.

To further assess the stability of the ligand–protein complexes, molecular dynamics (MD) simulations were performed, analysing their root mean square deviation (RMSD) and root mean square fluctuation (RMSF). Additionally, in silico ADMET predictions suggested that the synthesised derivatives possess favourable drug-like properties.

The significance of this study lies in its introduction of promising compounds—specifically **4u** (IC_50_ = 7.96 μM), **4t** (IC_50_ = 10.48 μM), **4m** (IC_50_ = 4.20 μM), and **4q** (IC_50_ = 8.00 μM)—that demonstrate specificity toward VEGFR2 kinase. This specificity highlights a promising route for the development of novel therapeutic agents for glioblastoma treatment. The findings and general indications of this study contribute to the ongoing efforts to address the critical need for more effective glioblastoma treatments, advancing the field of cancer therapy.

## Figures and Tables

**Table 1:** In vitro cell proliferation of compounds **4**(**a**–**w**) with their reagents against **U87-MG** and **U138-MG** cell lines.

No	Code	R	Cell Proliferation ± Std dev. (%)		No	Code	R	Cell Proliferation ± Std dev. (%)	
			U87	U138				U87	U138
**1**	**4a**	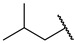	79 ± 1.3	84 ± 5.2	**13**	**4m**	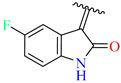	69 ± 2.1	54 ± 1.4
**2**	**4b**	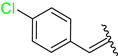	65 ± 4.9	60 ± 4.0	**14**	**4n**	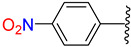	85 ± 1.2	92 ± 5.2
**3**	**4c**	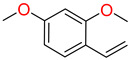	98 ± 2.5	75 ± 3.0	**15**	**4o**	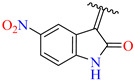	99 ± 7.0	83 ± 2.5
**4**	**4d**	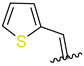	80 ± 4.5	70 ± 5.0	**16**	**4p**	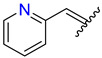	79 ± 1.4	81 ± 4.9
**5**	**4e**	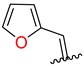	75 ± 3.1	90 ± 5.0	**17**	**4q**	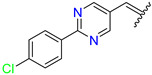	67 ± 4.9	39 ± 3.0
**6**	**4f**	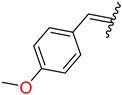	92 ± 1.4	90 ± 2.6	**18**	**4r**	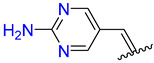	97 ± 1.3	92 ± 1.8
**7**	**4g**	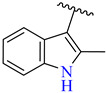	66 ± 4.9	54 ± 3.0	**19**	**4s**	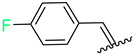	68 ± 5.0	68 ± 5.0
**8**	**4h**	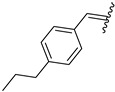	64 ± 5.2	100 ± 2.3	**20**	**4t**	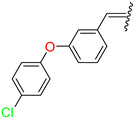	50 ± 3.2	65 ± 3.5
**9**	**4ı**	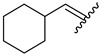	82 ± 5.5	68 ± 5.2	**21**	**4u**	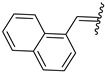	53 ± 6.4	65 ± 5.5
**10**	**4j**	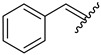	86 ± 2.6	74 ± 2.8	**22**	**4v**	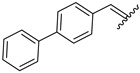	49 ± 5.5	59 ± 7.0
**11**	**4k**	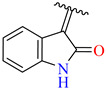	71± 2.1	95 ± 5	**23**	**4w**	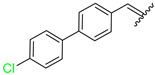	63 ± 2.5	80 ± 6.9
**12**	**4l**	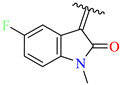	77 ± 7.0	73 ± 3.6	**24**	**TMZ**	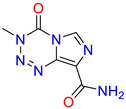	80 ± 5	78 ± 5

**Table 2:** Molecular docking values of the synthesised analogues with TMZ as a reference drug.

Molecules	VEGF Receptor (PDB ID: 2XIR)		
	Binding Affinity (kcal/mol)	Number of H-Bonds	H-Bonding Residues
**4a**	−9.0	2	Asp1046
**4b**	−10.3	1	Glu885
**4c**	−9.9	2	Glu885, Arg1027
**4d**	−8.4	3	Leu840, Asn923
**4e**	−9.2	2	Asp1046, Glu885
**4f**	−8.8	2	Asp1046, Glu885,
**4g**	−10.3	2	Asp1046, Glu885
**4h**	−10.2	2	Glu885, Asp1046
**4i**	−9.2	5	Asp1046, Ile1025, Arg1027, Glu885
**4j**	−10.1	1	Glu885
**4k**	−10.7	5	Asp1046, Arg1027, Glu885
**4l**	−9.0	4	Ala881, His1026, Arg1027, Ile1025
**4m**	−9.9	5	Asp1052, Leu840, Asn923, Cys919
**4n**	−10.2	1	Asp1046
**4o**	−9.3	1	Asp1046
**4p**	−9.9	2	Asp1046, Glu885
**4q**	−11.1	3	Asp1046, Glu885, Ile1025
**4r**	−9.4	2	Asp1046, Glu885
**4s**	−9.6	2	Asp1046, Glu885
**4t**	−11.4	4	Asp1046, Glu885, Ile1025
**4u**	−11.4	3	Asp1046, Glu885
**4v**	−10.4	2	Ser930, Cys919
**4w**	−12.2	1	Glu885
**TMZ**	−6.2	4	Leu840, Cys919

**Table 3:** In silico pharmacokinetic parameters obtained by the QikProp tool Schrödinger.

Comp.	CNS (−2 to +2)	Mw < 500	HBD (0–6)	HBA (2.0–20)	QPlogPo/w (−2.0–6.5)	QPlogS (−6.5–0.5)	QPPCaco <25 Poor >500 High	Metabsm (1–8)	%Human Oral Ab (>80% High <25% Poor)	N&O (<2 – 15>)	Rule of 5 (Max 4)	Rule of 3 (Max 3)
**4a**	−2	303.36	2	5.75	2.431	−4.886	209.18	5	82.715	6	0	0
**4b**	−2	357.796	2	5.75	3.319	−5.642	313.438	4	91.058	6	0	0
**4c**	−2	383.403	2	7.25	3.075	−5.791	296.37	6	89.191	8	0	1
**4d**	−2	329.373	2	5.75	2.765	−4.901	308.989	5	87.702	6	0	0
**4e**	−2	313.312	2	6.25	2.163	−4.157	301.102	5	83.972	7	0	0
**4f**	−2	353.377	2	6.5	2.938	−5.185	309.992	5	88.736	7	0	0
**4g**	−2	376.414	3	5.75	3.386	−5.97	228.303	5	88.981	7	0	1
**4h**	−2	365.431	2	5.75	3.849	−6.233	312.955	5	94.148	6	0	1
**4i**	−2	329.398	2	5.75	2.831	−5.617	203.198	5	84.828	6	0	0
**4j**	−2	323.351	2	5.75	2.831	−4.93	310.285	4	88.119	6	0	0
**4k**	−2	364.36	2	6.75	2.144	−5.335	49.12	4	69.77	8	0	0
**4l**	−2	396.377	1	7.25	2.775	−5.346	102.083	4	79.152	8	0	0
**4m**	−2	382.35	2	6.75	2.404	−5.603	54.475	4	72.098	8	0	0
**4n**	−2	368.348	2	6.75	2.135	−5.132	35.954	5	67.293	9	0	0
**4o**	−2	423.384	1	8.25	1.733	−4.265	27.701	5	49.954	11	1	0
**4p**	−2	323.351	2	5.75	2.828	−4.948	303.005	4	87.915	6	0	0
**4q**	−2	435.869	2	7.75	3.732	−6.758	171.738	6	88.794	8	0	1
**4r**	−2	340.341	4	8.25	0.686	−4.007	25.356	6	56.094	9	0	0
**4s**	−2	341.341	2	5.75	3.062	−5.289	310.103	4	89.47	6	0	0
**4t**	−2	449.893	2	6.25	4.882	−7.62	306.659	4	100	7	0	1
**4u**	−2	373.41	2	5.75	3.724	−6.051	304.844	4	93.21	6	0	1
**4v**	−2	399.448	2	5.75	4.409	−6.883	305.004	4	100	6	0	1
**4w**	−2	433.893	2	5.75	4.89	−7.596	303.939	4	100	6	0	1
**TMZ**	−2	194.152	2	7	−1.207	−1.379	59.807	1	51.677	8	0	0

**Table 4:** Blood–brain barrier (BBB) parameter obtained by the SwissADME, PreADME, and QikProp Schrödinger tools. HBD: Hydrogen bond doner, HBA: Hydrogen bond acceptor.

Molecules	BBB			
	PreADME	SwissADME	QikProp Schrödinger	BBB
**4a**	0.097/Low	No	Yes	Yes/Low
**4b**	0.149/Med	No	Yes	Yes/Low
**4c**	0.030/Low	No	Yes	Yes/Low
**4d**	0.0220/Low	No	Yes	Yes/Low
**4e**	0.0236/Low	No	Yes	Yes/Low
**4f**	0.0375/Low	No	Yes	Yes/Low
**4g**	0.314/Medl	No	Yes	Yes/Med
**4h**	0.394/Medl	No	Yes	Yes/Med
**4i**	0.258/Medl	No	Yes	Yes/Med
**4j**	0.062/Low	No	Yes	Yes/Low
**4k**	0.025/Low	No	Yes	Yes/Low
**4l**	0.0212/Low	No	Yes	Yes/Low
**4m**	0.024/Low	No	No	Yes/Low
**4n**	0.024/Low	No	No	Yes/Low
**4o**	1.061/Medl	Yes	No	Yes/Medl
**4p**	0.020/Low	No	Yes	Yes/Low
**4q**	0.015/Low	No	Yes	Yes/Low
**4r**	0.028/Low	No	No	Yes/Low
**4s**	0.082/Low	No	Yes	Yes/Low
**4t**	0.184/Medl	No	Yes	Yes/Medl
**4u**	0.090/Low	No	Yes	Yes/Low
**4v**	0.156/Medl	No	Yes	Yes/Medl
**4w**	0.450/Med	No	Yes	Yes/Medl
**TMZ**	0.017/Low	No	No	Yes/Low

**Table 5:** A summary comparing the key properties of the most promising compounds, including IC50 values, molecular docking scores, and ADME properties, as obtained using the QikProp tool from Schrödinger.

No.	Binding Affinity for VEGFR2 (kcal/mol)	IC_50_ Values (μM) U138	ADME						
			BBB	CNS (−2 to +2)	MolWt < 500	QPlogS (−6.5–0.5)	QPPCaco <25 Poor >500 High	Metabsm (1–8)	%Human OralAb >80% High <25% Poor
**4g**	−10.3	9.2	Yes	−2	376.41	−5.97	228.303	5	88.981
**4m**	−9.9	4.2	No	−2	382.35	−5.603	54.475	4	72.098
**4q**	−11.1	8.0	Yes	−2	435.86	−6.758	171.738	6	88.794
**TMZ**	−6.2	93.09	Yes	−2	194.15	−1.379	59.807	1	51.677

## Data Availability

The original contributions presented in this study are included in the article. Further inquiries can be directed to the corresponding author.

## Supplementary Materials

The following supporting information can be downloaded at: https://www.mdpi.com/article/10.3390/ph18020233/s1.
