# Supplementary material for: Design, Synthesis, and Computational Evaluation of 3,4-Dihydroquinolin-2(1H)-One Analogues as Potential VEGFR2 Inhibitors in Glioblastoma Multiforme
Source: Pharmaceuticals (Basel). 2025 Feb 8;18(2):233. doi: 10.3390/ph18020233 (PMC11859309; doi:10.3390/ph18020233)
Supplement: Supplementary file 1 [file pharmaceuticals-18-00233-s001.zip › pharmaceuticals-3414884-supplementary.pdf]

Supporting information

Design, Synthesis, and Computational Evaluation of 3,4-Dihydroquinolin-2(1H)-One Analogues as Potential VEGFR2 Inhibitors in Glioblastoma Multiforme

Shafeek Buhlak [1,†] , Nadeem Abad [1,†], Jihane Akachar [1], Sana Saffour [1], Yunus Kesgun [1], Sevval Dik [1], Betul Yasin [1] , Gizem Bati-Ayaz [1] , Essam Hanashalshahaby [1] , Hasan Türkez [2] and Adil Mardinoglu [3,4,*]

1 Trustlife Labs, Drug Research & Development Center, Istanbul 34774, Turkey; shafeekbuhlak@outlook.com (S.B.); abadnadeem3@gmail.com (N.A.); jihane.akachar@trustlifelabs.com (J.A.); sasaffour@gmail.com (S.S.); yunus.kesgun@trustlifelabs.com (Y.K.); sevvaldik8@gmail.com (S.D.); btlyasin@hotmail.com (B.Y.); batigizem@gmail.com (G.B.-A.); essam.hanash@trustlifelabs.com (E.H.)
2 Department of Medical Biology, Faculty of Medicine, Atatürk University, Erzurum 25240, Turkey; hturkez@atauni.edu.tr
3 Science for Life Laboratory, KTH-Royal Institute of Technology, SE-17165 Stockholm, Sweden
4 Centre for Host-Microbiome Interactions, Faculty of Dentistry, Oral & Craniofacial Sciences, King's College London, London SE1 9RT, UK
* Correspondence: adilm@scilifelab.se
† These authors contributed equally to this work.

Table of figures:

Figure 1. ^{1}H-NMR of compound (E)-N'-(3-methylbutylidene)-2-((2-oxo-1,2,3,4-tetrahydroquinolin-6-yl)oxy)acetohydrazide (**4a**) 5
Figure 2. ^{13}C-NMR of (E)-N'-(3-methylbutylidene)-2-((2-oxo-1,2,3,4-tetrahydroquinolin-6-yl)oxy)acetohydrazide (**4a**) 5
Figure 3. Mass spectroscopy of (E)-N'-(3-methylbutylidene)-2-((2-oxo-1,2,3,4-tetrahydroquinolin-6-yl)oxy)acetohydrazide (**4a**) 6
Figure 4. ^{1}H-NMR of (E)-N'-(4-chlorobenzylidene)-2-((2-oxo-1,2,3,4-tetrahydroquinolin-6-yl) oxy) acetohydrazide (**4b**) 6
Figure 5. ^{13}C-NMR of (E)-N'-(4-chlorobenzylidene)-2-((2-oxo-1,2,3,4-tetrahydroquinolin-6-yl) oxy) acetohydrazide (**4b**) 7
Figure 6. Mass spectroscopy of (E)-N'-(4-chlorobenzylidene)-2-((2-oxo-1,2,3,4-tetrahydroquinolin-6-yl) oxy) acetohydrazide (**4b**) 7
Figure 7. ^{1}H-NMR of N'-(2,4-dimethoxybenzylidene)-2-((2-oxo-1,2,3,4-tetrahydroquinolin-6-yl)oxy) acetohydrazide (**4c**) 8
Figure 8. ^{13}C-NMR of N'-(2,4-dimethoxybenzylidene)-2-((2-oxo-1,2,3,4-tetrahydroquinolin-6-yl)oxy) acetohydrazide (**4c**) 8
Figure 9. Mass spectroscopy of N'-(2,4-dimethoxybenzylidene)-2-((2-oxo-1,2,3,4-tetrahydroquinolin-6-yl)oxy) acetohydrazide (**4c**) 9
Figure 10. ^{1}H-NMR of 2-((2-oxo-1,2,3,4-tetrahydroquinolin-6-yl)oxy)-N'-(thiophen-2-ylmethylene)acetohydrazide (**4d**) 9
Figure 11. ^{13}C-NMR of 2-((2-oxo-1,2,3,4-tetrahydroquinolin-6-yl)oxy)-N'-(thiophen-2-ylmethylene)acetohydrazide (**4d**) 10
Figure 12. Mass spectoscopy of 2-((2-oxo-1,2,3,4-tetrahydroquinolin-6-yl)oxy)-N'-(thiophen-2-ylmethylene)acetohydrazide (**4d**) 10
Figure 13. ^{1}H-NMR of (E)-N'-(furan-2-ylmethylene)-2-((2-oxo-1,2,3,4-tetrahydroquinolin-6-yl)oxy)acetohydrazide (**4e**) 11
Figure 14. ^{13}C-NMR of (E)-N'-(furan-2-ylmethylene)-2-((2-oxo-1,2,3,4-tetrahydroquinolin-6-yl)oxy)acetohydrazide (**4e**) 11
Figure 15. Mass Spectroscopy of (E)-N'-(furan-2-ylmethylene)-2-((2-oxo-1,2,3,4-tetrahydroquinolin-6-yl)oxy)acetohydrazide (**4e**) 12
Figure 16. ^{1}H-NMR of (E)-N'-(4-methoxybenzylidene)-2-((2-oxo-1,2,3,4-tetrahydroquinolin-6-yl)oxy)acetohydrazide (**4f**) 12
Figure 17. ^{13}C-NMR of (E)-N'-(4-methoxybenzylidene)-2-((2-oxo-1,2,3,4-tetrahydroquinolin-6-yl)oxy)acetohydrazide (**4f**) 13
Figure 18. Mass spectroscopy of (E)-N'-(4-methoxybenzylidene)-2-((2-oxo-1,2,3,4-tetrahydroquinolin-6-yl)oxy)acetohydrazide (**4f**) 13
Figure 19. ^{1}H-NMR of (E)-N'-((2-methyl-1H-indol-3-yl)methylene)-2-((2-oxo-1,2,3,4-tetrahydroquinolin-6-yl)oxy)acetohydrazide (**4g**) 14
Figure 20. ^{13}C-NMR of (E)-N'-((2-methyl-1H-indol-3-yl)methylene)-2-((2-oxo-1,2,3,4-tetrahydroquinolin-6-yl)oxy)acetohydrazide (**4g**) 14
Figure 21. Mass spectroscopy of (E)-N'-((2-methyl-1H-indol-3-yl)methylene)-2-((2-oxo-1,2,3,4-tetrahydroquinolin-6-yl)oxy)acetohydrazide (**4g**) 15
Figure 22. LCMS/MS of compound (E)-N'-((2-methyl-1H-indol-3-yl)methylene)-2-((2-oxo-1,2,3,4-tetrahydroquinolin-6-yl)oxy)acetohydrazide (**4g**) showing 99.33% purity. 15

Figure 23. ^{1}H-NMR of (E)-2-((2-oxo-1,2,3,4-tetrahydroquinolin-6-yl)oxy)-N'-(4-propylbenzylidene)acetohydrazide (**4h**) 16
Figure 24. ^{13}C-NMR of (E)-2-((2-oxo-1,2,3,4-tetrahydroquinolin-6-yl)oxy)-N'-(4-propylbenzylidene)acetohydrazide (**4h**) 16
Figure 25. Mass spectroscopy of (E)-2-((2-oxo-1,2,3,4-tetrahydroquinolin-6-yl)oxy)-N'-(4-propylbenzylidene)acetohydrazide (**4h**) 17
Figure 26. ^{1}H-NMR of (E)-N'-(cyclohexylmethylene)-2-((2-oxo-1,2,3,4-tetrahydroquinolin-6-yl)oxy)acetohydrazide (**4i**) 17
Figure 27. ^{13}C-NMR of (E)-N'-(cyclohexylmethylene)-2-((2-oxo-1,2,3,4-tetrahydroquinolin-6-yl)oxy)acetohydrazide (**4i**) 18
Figure 28. Mass spectroscopy of (E)-N'-(cyclohexylmethylene)-2-((2-oxo-1,2,3,4-tetrahydroquinolin-6-yl)oxy)acetohydrazide (**4i**) 18
Figure 29. ^{1}H-NMR of (E)-N'-benzylidene-2-((2-oxo-1,2,3,4-tetrahydroquinolin-6-yl)oxy)acetohydrazide (**4j**) 19
Figure 30. ^{13}C-NMR of (E)-N'-benzylidene-2-((2-oxo-1,2,3,4-tetrahydroquinolin-6-yl)oxy)acetohydrazide (**4j**) 19
Figure 31. Mass spectroscopy of(E)-N'-benzylidene-2-((2-oxo-1,2,3,4-tetrahydroquinolin-6-yl)oxy)acetohydrazide (**4j**) 20
Figure 32. ^{1}H-NMR of (Z)-2-((2-oxo-1,2,3,4-tetrahydroquinolin-6-yl)oxy)-N'-(2-oxoindolin-3-ylidene)acetohydrazide (**4k**) 20
Figure 33. ^{13}C-NMR of (Z)-2-((2-oxo-1,2,3,4-tetrahydroquinolin-6-yl)oxy)-N'-(2-oxoindolin-3-ylidene)acetohydrazide (**4k**) 21
Figure 34. Mass spectroscopy of (Z)-2-((2-oxo-1,2,3,4-tetrahydroquinolin-6-yl)oxy)-N'-(2-oxoindolin-3-ylidene)acetohydrazide (**4k**) 21
Figure 35. Mass spectroscopy of (Z)-N'-(5-fluoro-1-methyl-2-oxoindolin-3-ylidene)-2-((2-oxo-1,2,3,4-tetrahydroquinolin-6-yl)oxy)acetohydrazide (**4l**) 22
Figure 36. LCMS/MS of compound (Z)-N'-(5-fluoro-1-methyl-2-oxoindolin-3-ylidene)-2-((2-oxo-1,2,3,4-tetrahydroquinolin-6-yl)oxy)acetohydrazide (**4l**) showing 95.36% purity. 22
Figure 37. ^{1}H-NMR of (Z)-N'-(5-fluoro-2-oxoindolin-3-ylidene)-2-((2-oxo-1,2,3,4-tetrahydroquinolin-6-yl)oxy)acetohydrazide (**4m**) 23
Figure 38. ^{13}C-NMR of (Z)-N'-(5-fluoro-2-oxoindolin-3-ylidene)-2-((2-oxo-1,2,3,4-tetrahydroquinolin-6-yl)oxy)acetohydrazide (**4m**) 23
Figure 39. Mass spectroscopy of (Z)-N'-(5-fluoro-2-oxoindolin-3-ylidene)-2-((2-oxo-1,2,3,4-tetrahydroquinolin-6-yl)oxy)acetohydrazide (**4m**) 24
Figure 40. ^{1}H-NMR of (E)-N'-(4-nitrobenzylidene)-2-((2-oxo-1,2,3,4-tetrahydroquinolin-6-yl)oxy)acetohydrazide (**4n**) 24
Figure 41. ^{13}C-NMR of (E)-N'-(4-nitrobenzylidene)-2-((2-oxo-1,2,3,4-tetrahydroquinolin-6-yl)oxy)acetohydrazide (**4n**) 25
Figure 42. Mass spectroscopy of (E)-N'-(4-nitrobenzylidene)-2-((2-oxo-1,2,3,4-tetrahydroquinolin-6-yl)oxy)acetohydrazide (**4n**) 25
Figure 43. Mass spectroscopy of (Z)-N'-(5-nitro-2-oxoindolin-3-ylidene)-2-((2-oxo-1,2,3,4-tetrahydroquinolin-6-yl)oxy)acetohydrazide (**4o**) 26
Figure 44. ^{1}H-NMR of (E)-2-((2-oxo-1,2,3,4-tetrahydroquinolin-6-yl)oxy)-N'-(pyridin-2-ylmethylene)acetohydrazide (**4p**) 26
Figure 45. ^{13}C-NMR of (E)-2-((2-oxo-1,2,3,4-tetrahydroquinolin-6-yl)oxy)-N'-(pyridin-2-ylmethylene)acetohydrazide (**4p**) 27
Figure 46. Mass spectroscopy of (E)-2-((2-oxo-1,2,3,4-tetrahydroquinolin-6-yl)oxy)-N'-(pyridin-2-ylmethylene)acetohydrazide (**4p**) 27

Figure 47. ^{1}H-NMR of (E)-N'-((2-(4-chlorophenyl)pyrimidin-5-yl)methylene)-2-((2-oxo-1,2,3,4-tetrahydroquinolin-6-yl)oxy)acetohydrazide (**4q**) ... 28
Figure 48. ^{13}C-NMR of (E)-N'-((2-(4-chlorophenyl)pyrimidin-5-yl)methylene)-2-((2-oxo-1,2,3,4-tetrahydroquinolin-6-yl)oxy)acetohydrazide (**4q**) ... 28
Figure 49. Mass spectroscopy of (E)-N'-((2-(4-chlorophenyl)pyrimidin-5-yl)methylene)-2-((2-oxo-1,2,3,4-tetrahydroquinolin-6-yl)oxy)acetohydrazide (**4q**) ... 29
Figure 50. ^{1}H-NMR of (E)-N'-((2-aminopyrimidin-5-yl)methylene)-2-((2-oxo-1,2,3,4-tetrahydroquinolin-6-yl)oxy)acetohydrazide (**4r**) ... 29
Figure 51. ^{13}C-NMR of (E)-N'-((2-aminopyrimidin-5-yl)methylene)-2-((2-oxo-1,2,3,4-tetrahydroquinolin-6-yl)oxy)acetohydrazide (**4r**) ... 30
Figure 52. Mass spectroscopy of (E)-N'-((2-aminopyrimidin-5-yl)methylene)-2-((2-oxo-1,2,3,4-tetrahydroquinolin-6-yl)oxy)acetohydrazide (**4r**) ... 30
Figure 53. LCMS/MS of compound (E)-N'-((2-aminopyrimidin-5-yl)methylene)-2-((2-oxo-1,2,3,4-tetrahydroquinolin-6-yl)oxy)acetohydrazide (**4r**) showing 97.54% purity. ... 31
Figure 54. ^{1}H-NMR of (E)-N'-(4-fluorobenzylidene)-2-((2-oxo-1,2,3,4-tetrahydroquinolin-6-yl)oxy)acetohydrazide (**4s**) ... 31
Figure 55. ^{13}C-NMR of (E)-N'-(4-fluorobenzylidene)-2-((2-oxo-1,2,3,4-tetrahydroquinolin-6-yl)oxy)acetohydrazide (**4s**) ... 32
Figure 56. Mass spectroscopy of (E)-N'-(4-fluorobenzylidene)-2-((2-oxo-1,2,3,4-tetrahydroquinolin-6-yl)oxy)acetohydrazide (**4s**) ... 32
Figure 57. ^{1}H-NMR of (E)-N'-(3-(4-chlorophenoxy)benzylidene)-2-((2-oxo-1,2,3,4-tetrahydroquinolin-6-yl)oxy)acetohydrazide (**4t**) ... 33
Figure 58. ^{13}C-NMR of (E)-N'-(3-(4-chlorophenoxy)benzylidene)-2-((2-oxo-1,2,3,4-tetrahydroquinolin-6-yl)oxy)acetohydrazide (**4t**) ... 33
Figure 59. Mass spectroscopy of (E)-N'-(3-(4-chlorophenoxy)benzylidene)-2-((2-oxo-1,2,3,4-tetrahydroquinolin-6-yl)oxy)acetohydrazide (**4t**) ... 34
Figure 60. ^{1}H-NMR of (E)-N'-(naphthalen-1-ylmethylene)-2-((2-oxo-1,2,3,4-tetrahydroquinolin-6-yl)oxy)acetohydrazide (**4u**) ... 34
Figure 61. ^{13}C-NMR of (E)-N'-(naphthalen-1-ylmethylene)-2-((2-oxo-1,2,3,4-tetrahydroquinolin-6-yl)oxy)acetohydrazide (**4u**) ... 35
Figure 62. Mass spectroscopy of (E)-N'-(naphthalen-1-ylmethylene)-2-((2-oxo-1,2,3,4-tetrahydroquinolin-6-yl)oxy)acetohydrazide (**4u**) ... 35
Figure 63. ^{1}H-NMR of (E)-N'-([1,1'-biphenyl]-4-ylmethylene)-2-((2-oxo-1,2,3,4-tetrahydroquinolin-6-yl)oxy)acetohydrazide (**4v**) ... 36
Figure 64. ^{13}C-NMR of (E)-N'-([1,1'-biphenyl]-4-ylmethylene)-2-((2-oxo-1,2,3,4-tetrahydroquinolin-6-yl)oxy)acetohydrazide (**4v**) ... 36
Figure 65. Mass spectroscopy of (E)-N'-([1,1'-biphenyl]-4-ylmethylene)-2-((2-oxo-1,2,3,4-tetrahydroquinolin-6-yl)oxy)acetohydrazide (**4v**) ... 37
Figure 66. ^{1}H-NMR of (E)-N'-((4'-chloro-[1,1'-biphenyl]-4-yl)methylene)-2-((2-oxo-1,2,3,4-tetrahydroquinolin-6-yl)oxy)acetohydrazide (**4w**) ... 37
Figure 67. ^{13}C-NMR of (E)-N'-((4'-chloro-[1,1'-biphenyl]-4-yl)methylene)-2-((2-oxo-1,2,3,4-tetrahydroquinolin-6-yl)oxy)acetohydrazide (**4w**) ... 38
Figure 68. Mass spectroscopy of (E)-N'-((4'-chloro-[1,1'-biphenyl]-4-yl)methylene)-2-((2-oxo-1,2,3,4-tetrahydroquinolin-6-yl)oxy)acetohydrazide (**4w**) ... 38

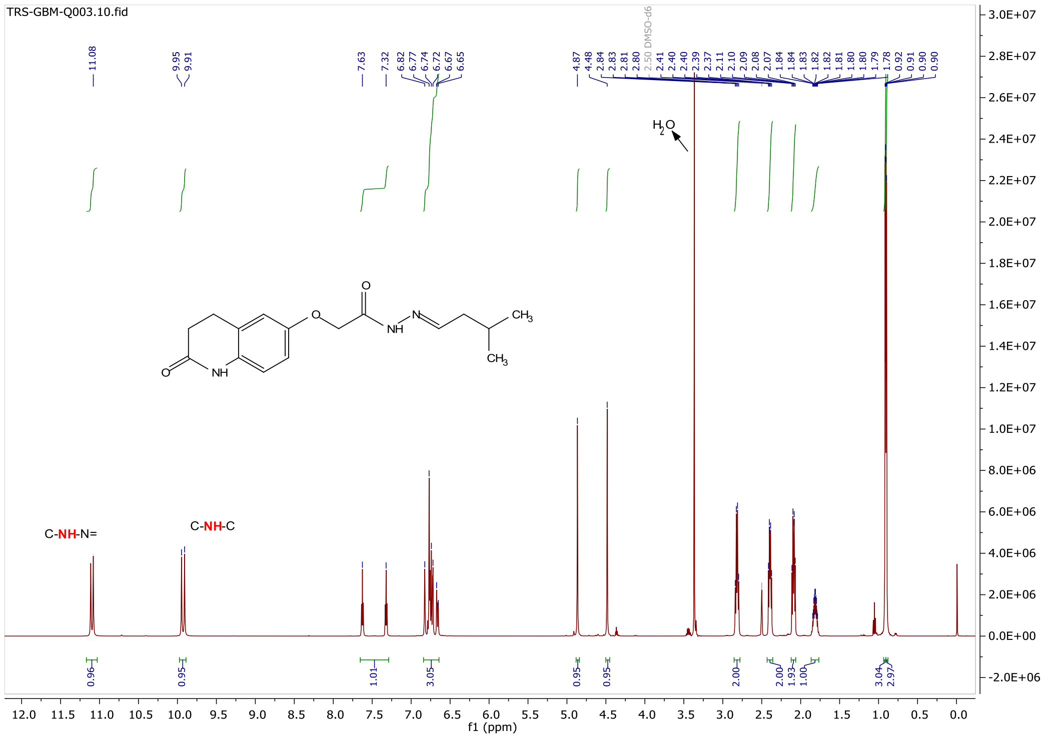

*Figure 1. ^{1}H-NMR of compound (E)-N'-(3-methylbutylidene)-2-((2-oxo-1,2,3,4-tetrahydroquinolin-6-yl)oxy)acetohydrazide (**4a**)*

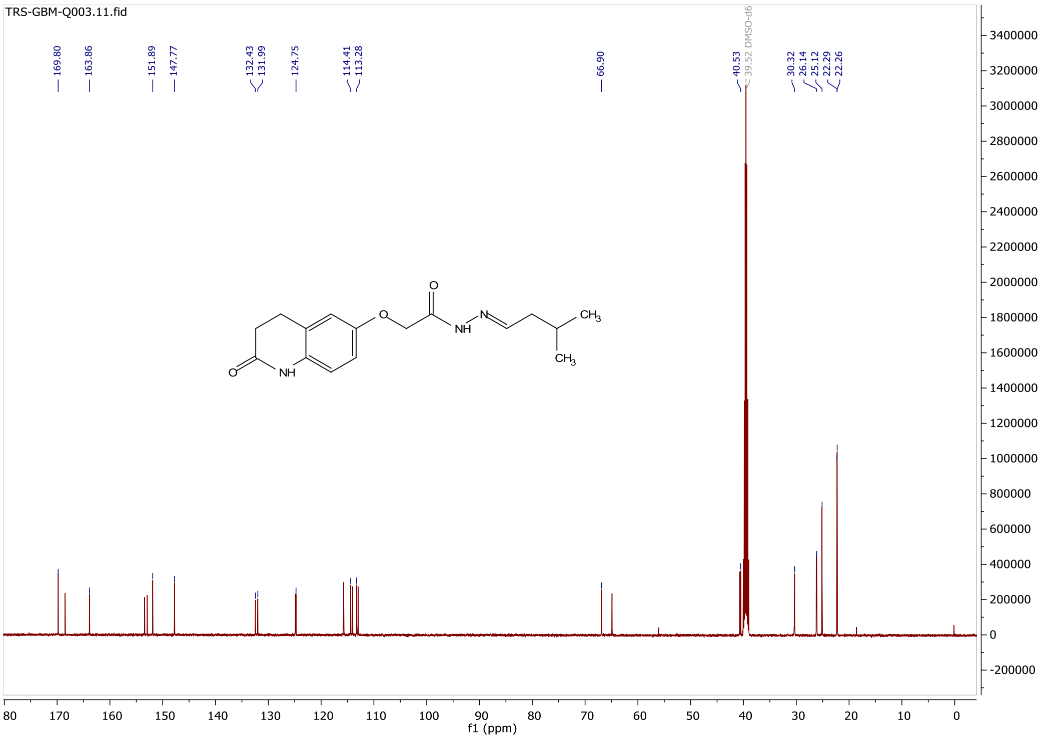

*Figure 2. ^{13}C-NMR of (E)-N'-(3-methylbutylidene)-2-((2-oxo-1,2,3,4-tetrahydroquinolin-6-yl)oxy)acetohydrazide (**4a**)*

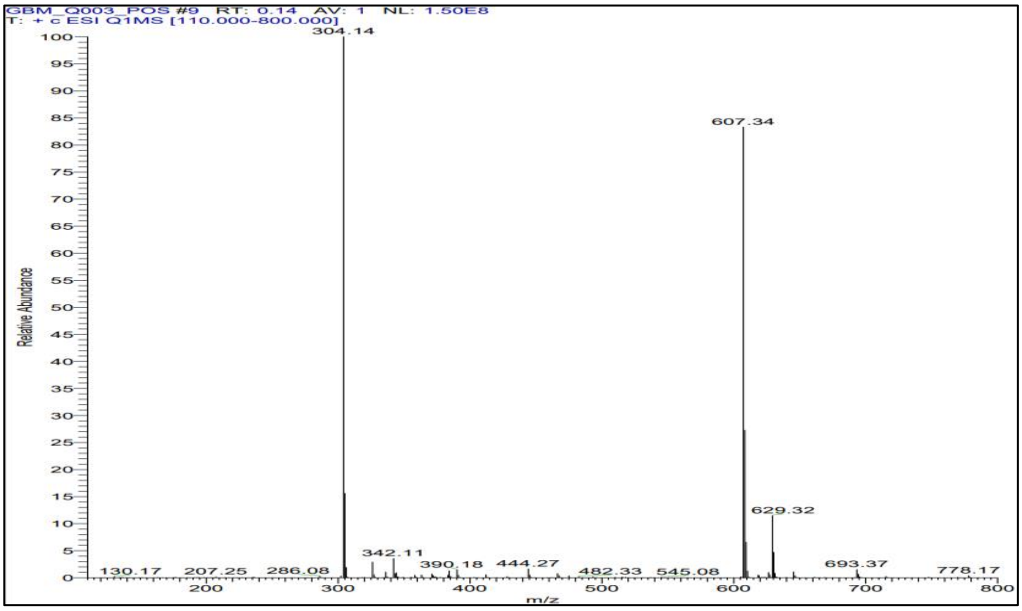

*Figure 3. Mass spectroscopy of (E)-N'-(3-methylbutylidene)-2-((2-oxo-1,2,3,4-tetrahydroquinolin-6-yl)oxy)acetohydrazide (**4a**)*

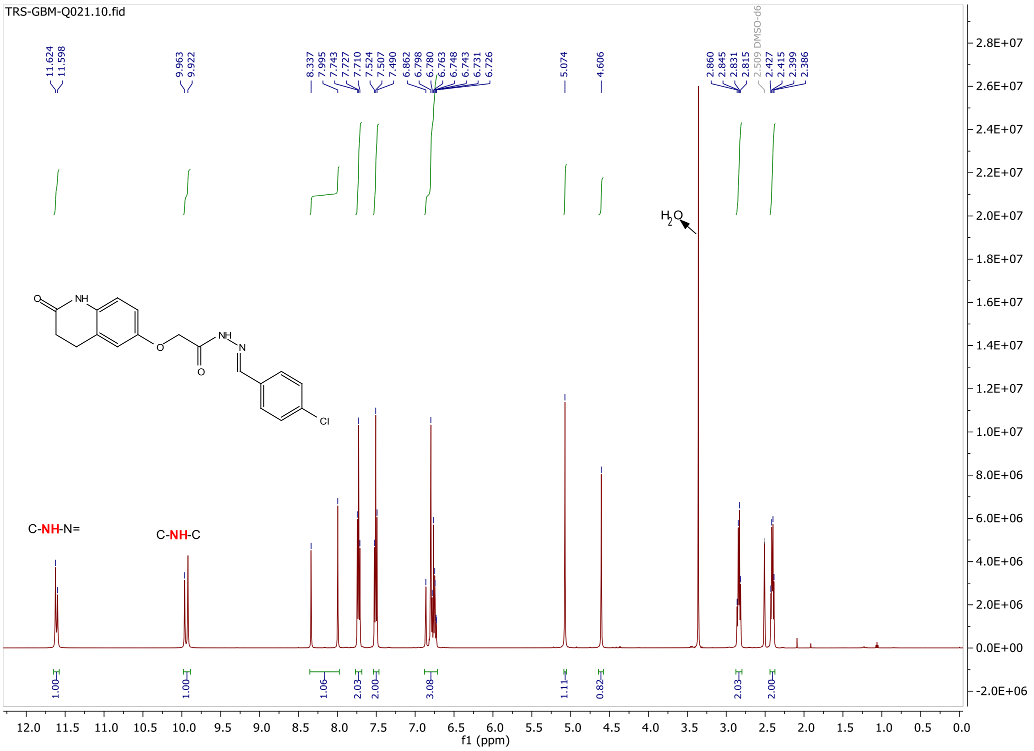

*Figure 4. ^{1}H-NMR of (E)-N'-(4-chlorobenzylidene)-2-((2-oxo-1,2,3,4-tetrahydroquinolin-6-yl) oxy) acetohydrazide (**4b**)*

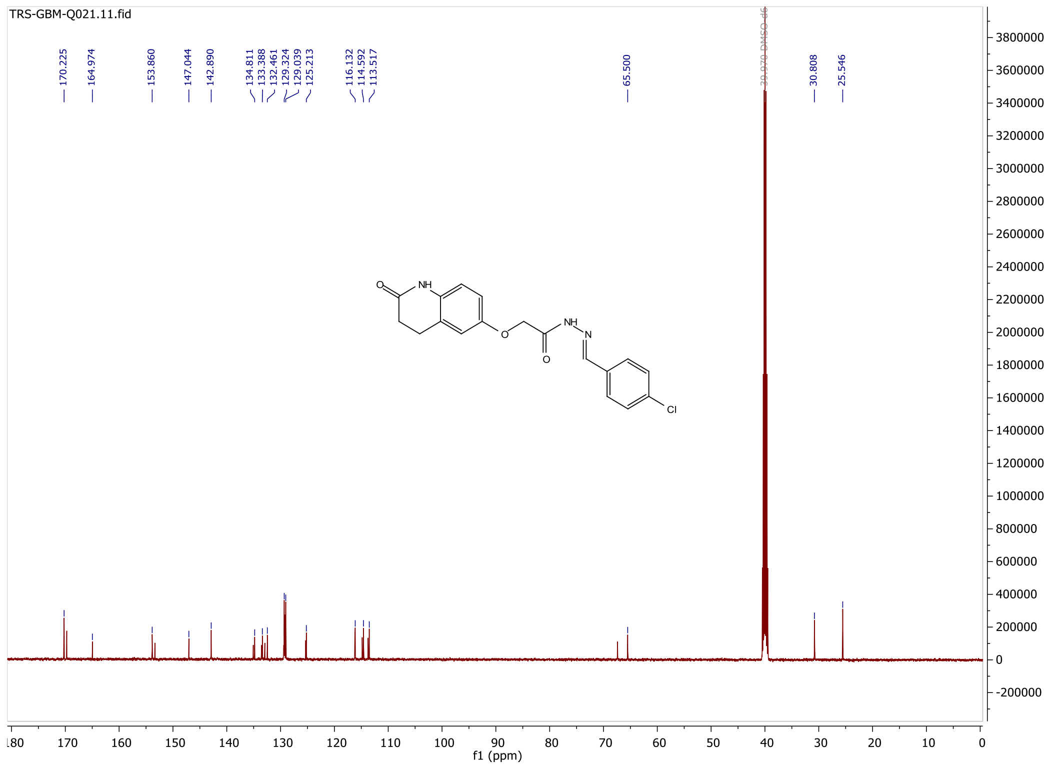

Figure 5. ^{13}C-NMR of (E)-N'-(4-chlorobenzylidene)-2-((2-oxo-1,2,3,4-tetrahydroquinolin-6-yl) oxy) acetohydrazide (**4b**)

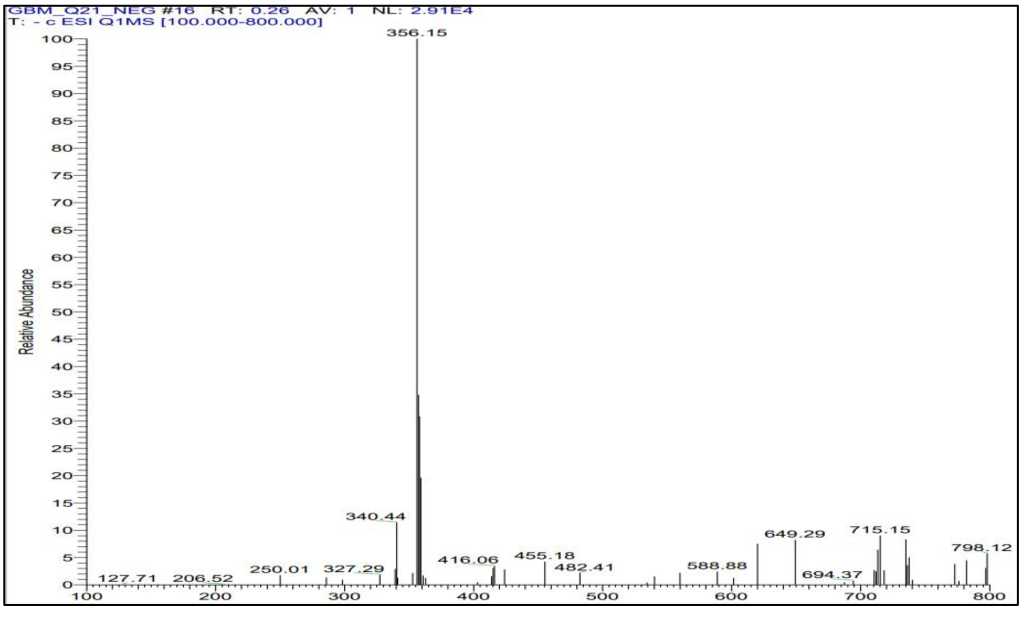

Figure 6. Mass spectroscopy of (E)-N'-(4-chlorobenzylidene)-2-((2-oxo-1,2,3,4-tetrahydroquinolin-6-yl) oxy) acetohydrazide (**4b**)

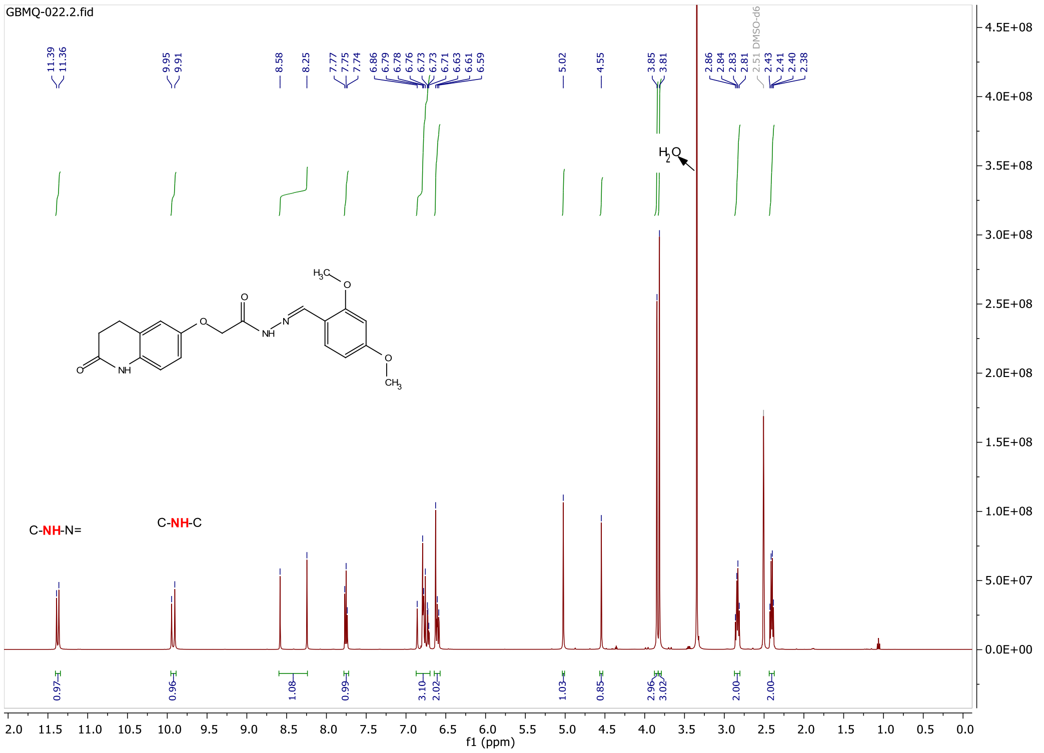

*Figure 7. ^{1}H-NMR of N'-(2,4-dimethoxybenzylidene)-2-((2-oxo-1,2,3,4-tetrahydroquinolin-6-yl)oxy) acetohydrazide (**4c**)*

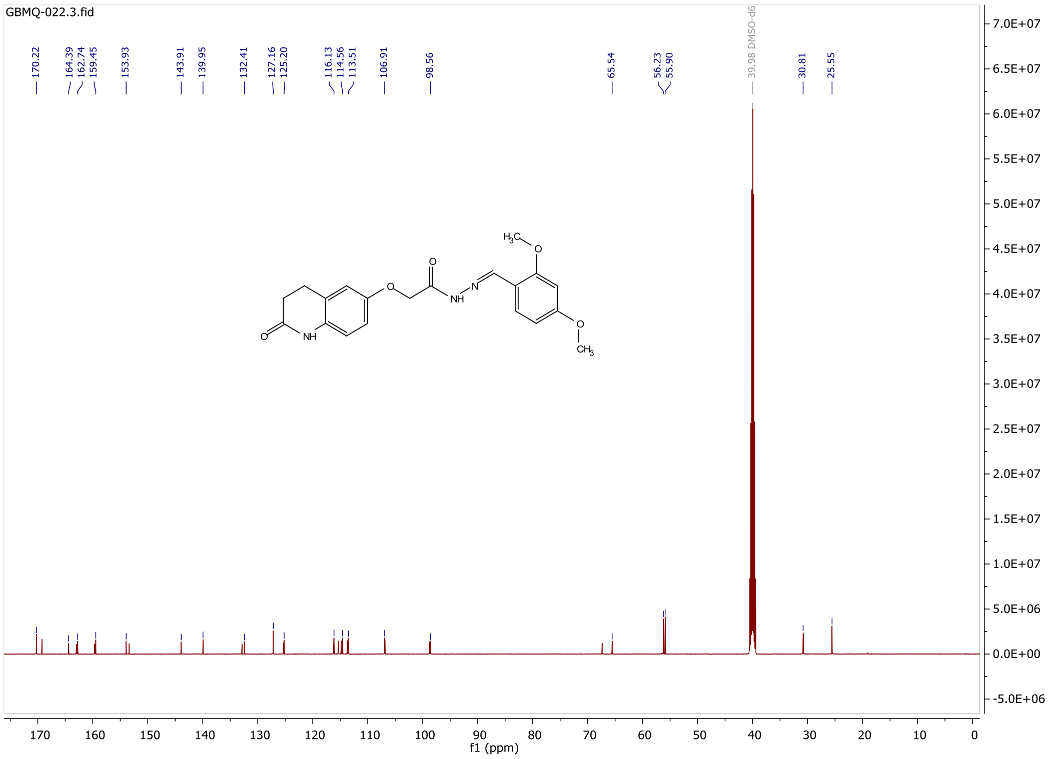

*Figure 8. ^{13}C-NMR of N'-(2,4-dimethoxybenzylidene)-2-((2-oxo-1,2,3,4-tetrahydroquinolin-6-yl)oxy) acetohydrazide (**4c**)*

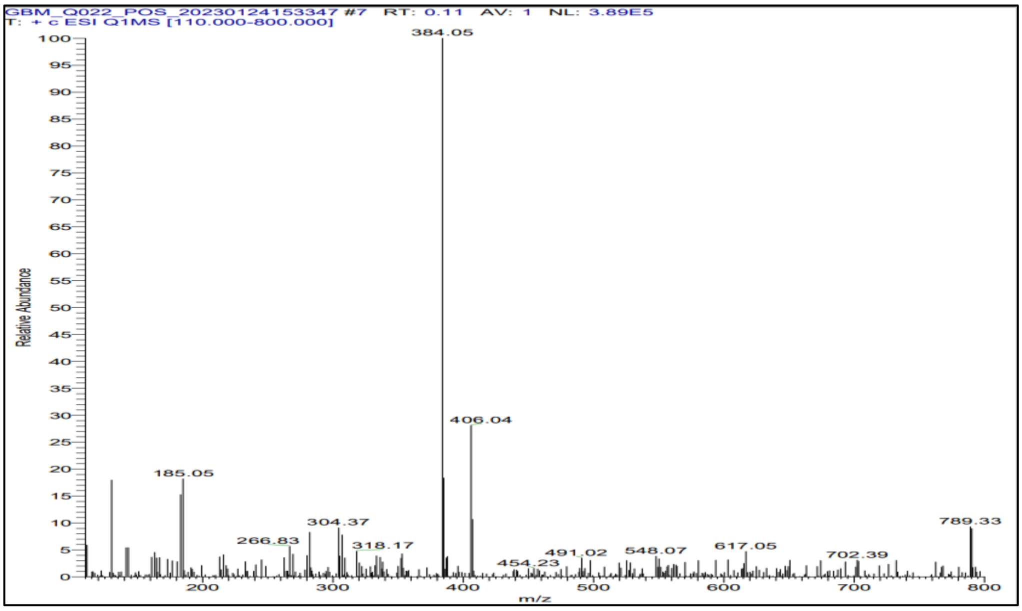

Figure 9. Mass spectroscopy of N'-(2,4-dimethoxybenzylidene)-2-((2-oxo-1,2,3,4-tetrahydroquinolin-6-yl)oxy) acetohydrazide (**4c**)

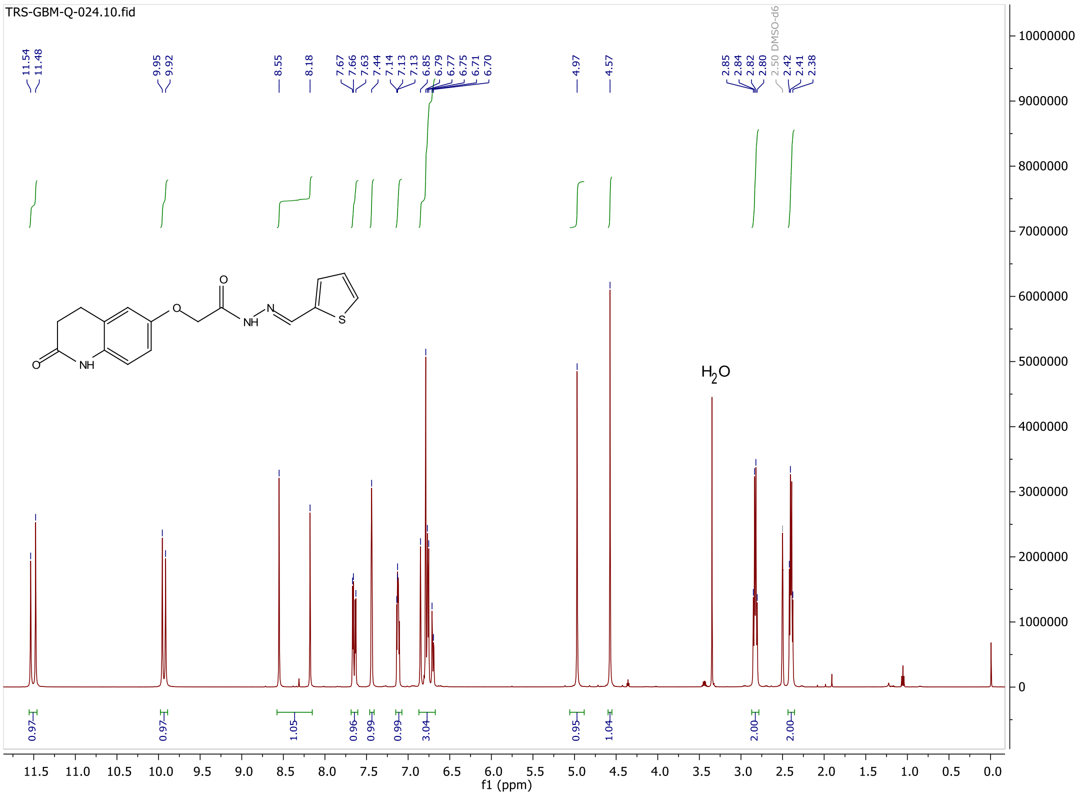

Figure 10. ^{1}H-NMR of 2-((2-oxo-1,2,3,4-tetrahydroquinolin-6-yl)oxy)-N'-(thiophen-2-ylmethylene)acetohydrazide (**4d**)

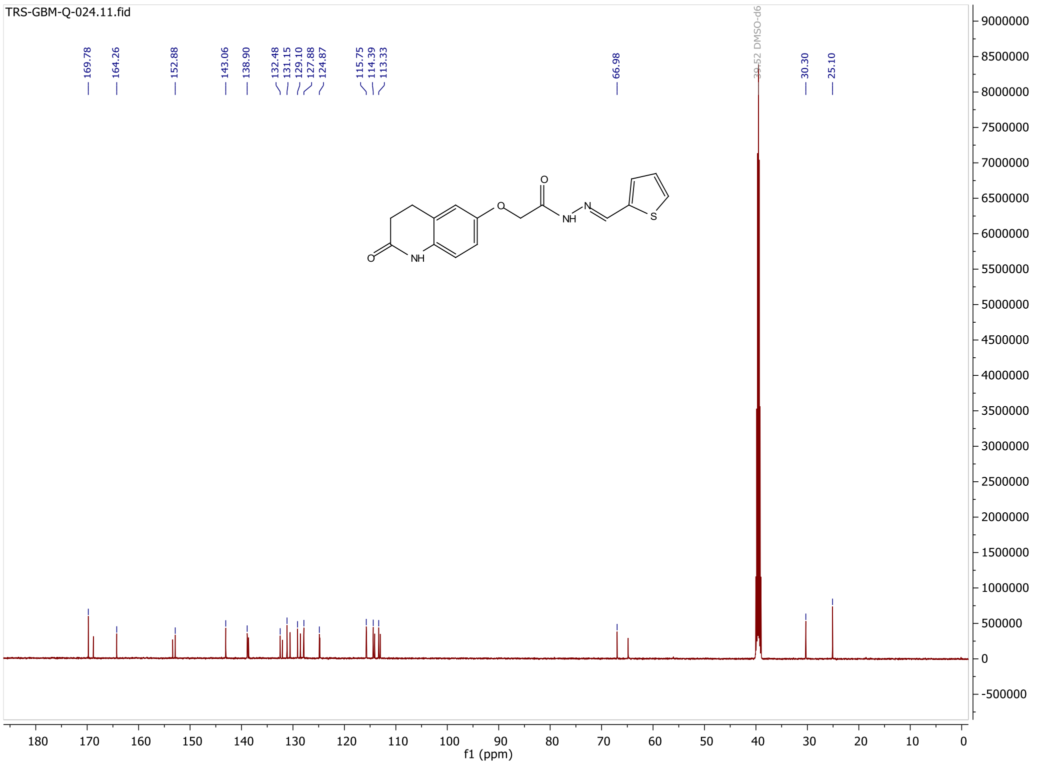

*Figure 11. ^{13}C-NMR of 2-((2-oxo-1,2,3,4-tetrahydroquinolin-6-yl)oxy)-N'-(thiophen-2-ylmethylene)acetohydrazide (**4d**)*

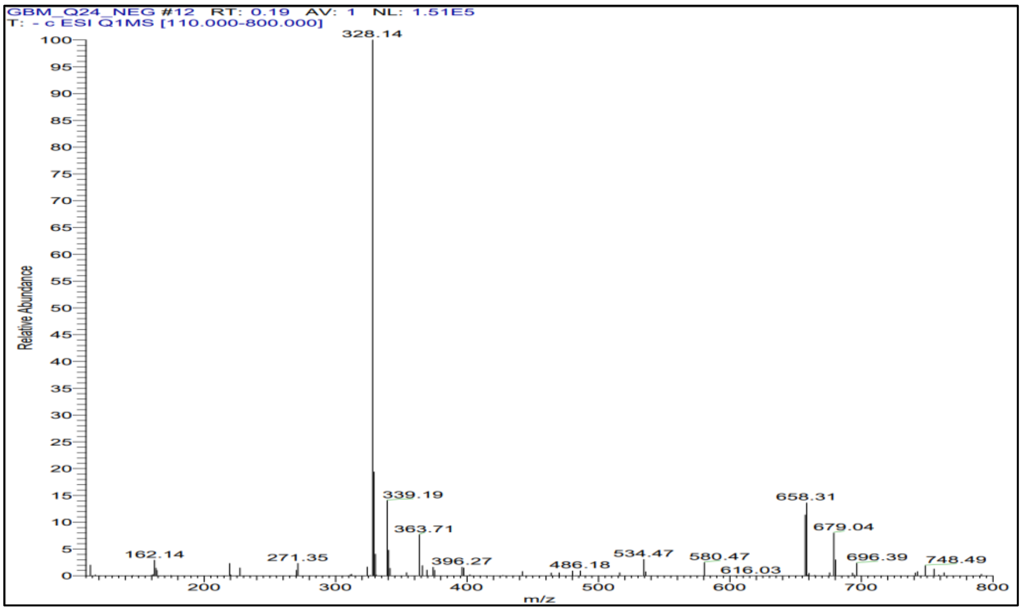

*Figure 12. Mass spectoscopy of 2-((2-oxo-1,2,3,4-tetrahydroquinolin-6-yl)oxy)-N'-(thiophen-2-ylmethylene)acetohydrazide (**4d**)*

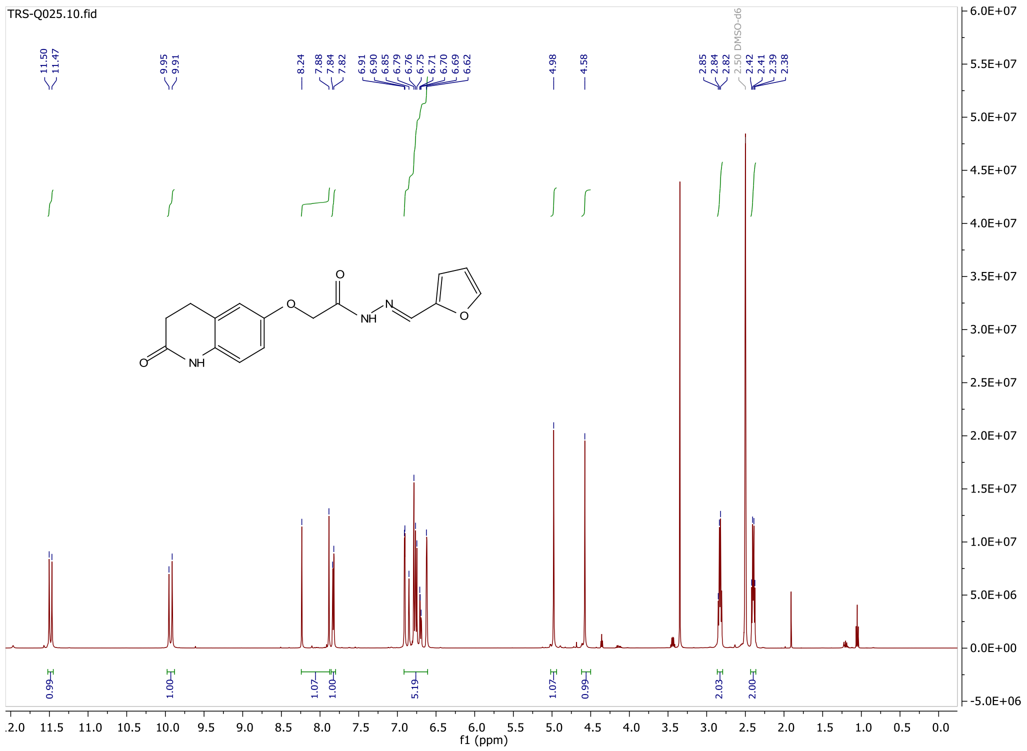

Figure 13. ^{1}H-NMR of (E)-N'-(furan-2-ylmethylene)-2-((2-oxo-1,2,3,4-tetrahydroquinolin-6-yl)oxy)acetohydrazide (**4e**)

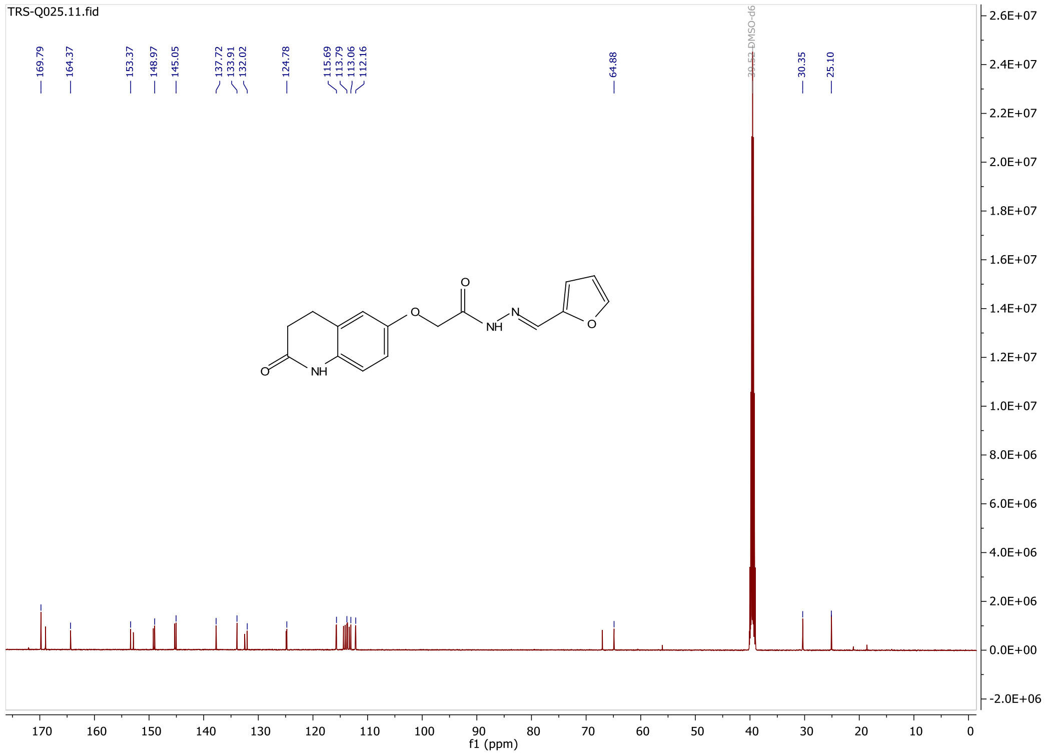

Figure 14. ^{13}C-NMR of (E)-N'-(furan-2-ylmethylene)-2-((2-oxo-1,2,3,4-tetrahydroquinolin-6-yl)oxy)acetohydrazide (**4e**)

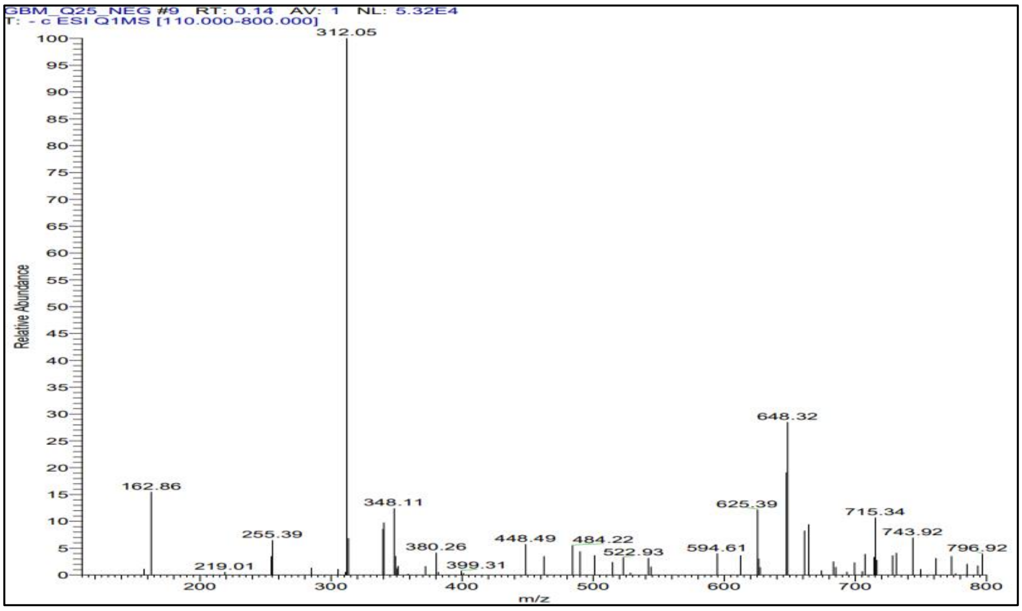

*Figure 15. Mass Spectroscopy of (E)-N'-(furan-2-ylmethylene)-2-((2-oxo-1,2,3,4-tetrahydroquinolin-6-yl)oxy)acetohydrazide (**4e**)*

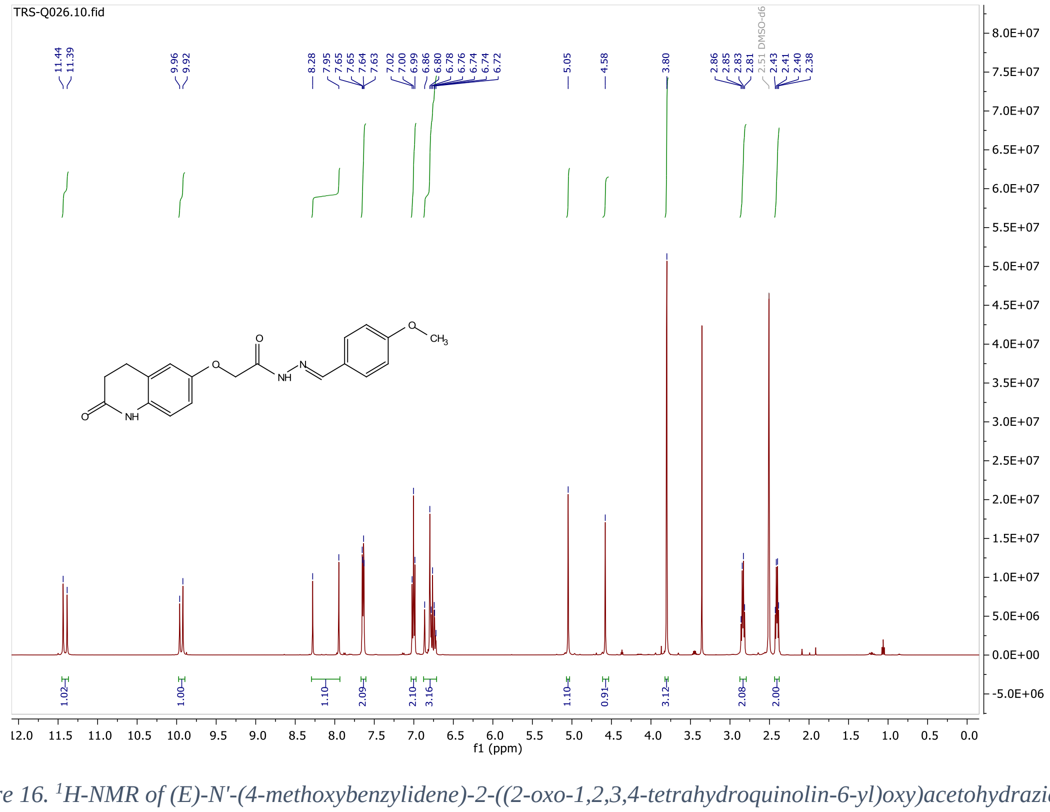

*Figure 16. ^{1}H-NMR of (E)-N'-(4-methoxybenzylidene)-2-((2-oxo-1,2,3,4-tetrahydroquinolin-6-yl)oxy)acetohydrazide (**4f**)*

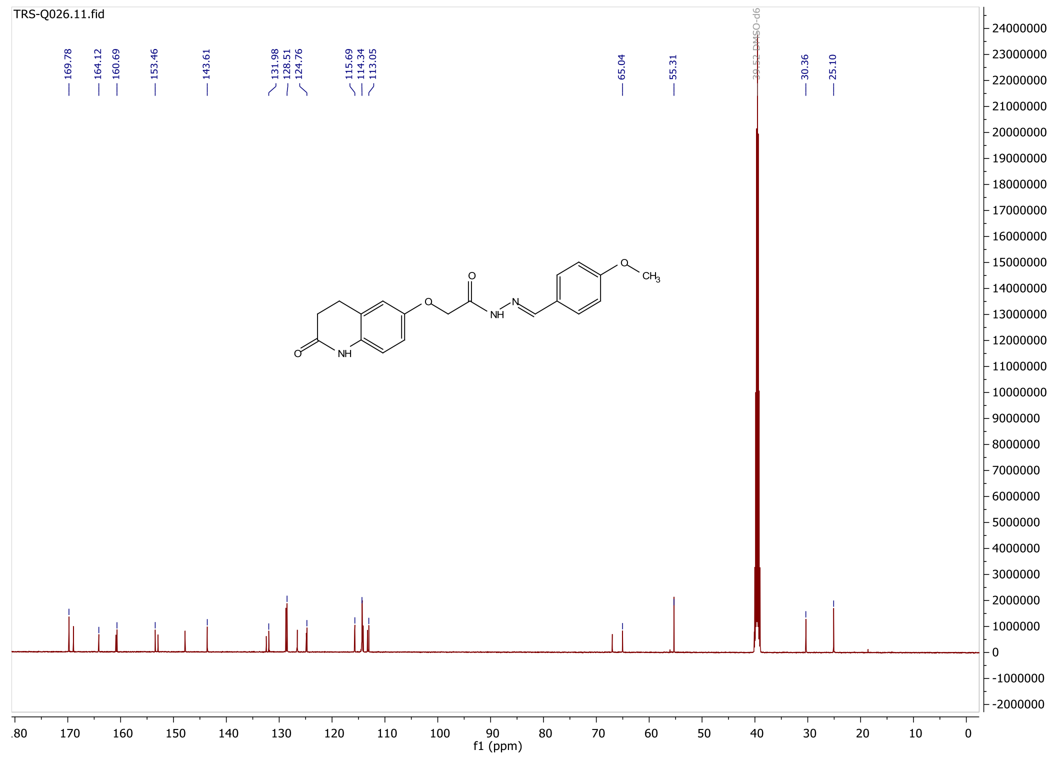

*Figure 17. ^{13}C-NMR of (E)-N'-(4-methoxybenzylidene)-2-((2-oxo-1,2,3,4-tetrahydroquinolin-6-yl)oxy)acetohydrazide (**4f**)*

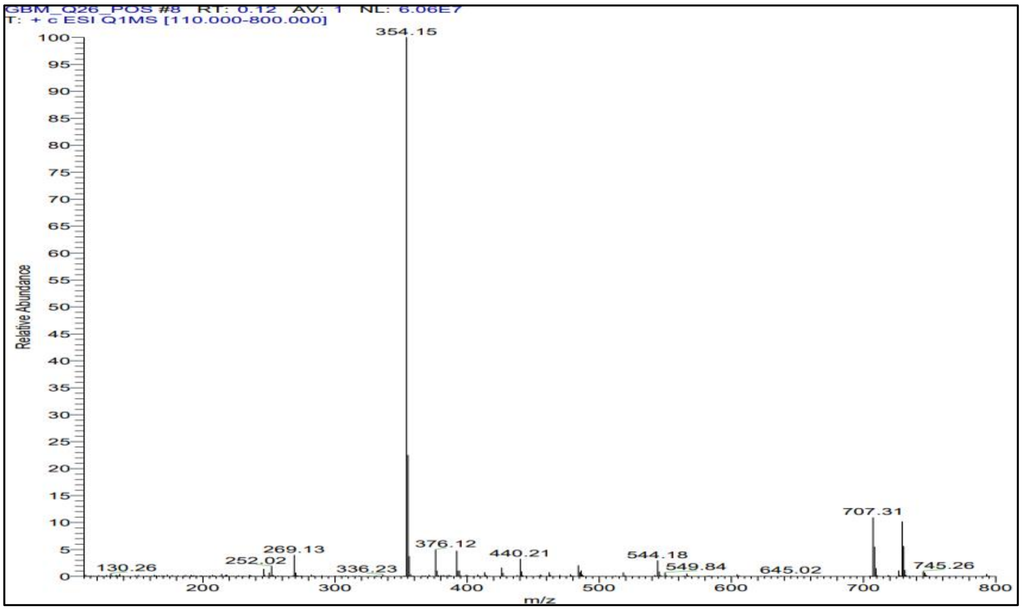

*Figure 18. Mass spectroscopy of (E)-N'-(4-methoxybenzylidene)-2-((2-oxo-1,2,3,4-tetrahydroquinolin-6-yl)oxy)acetohydrazide (**4f**)*

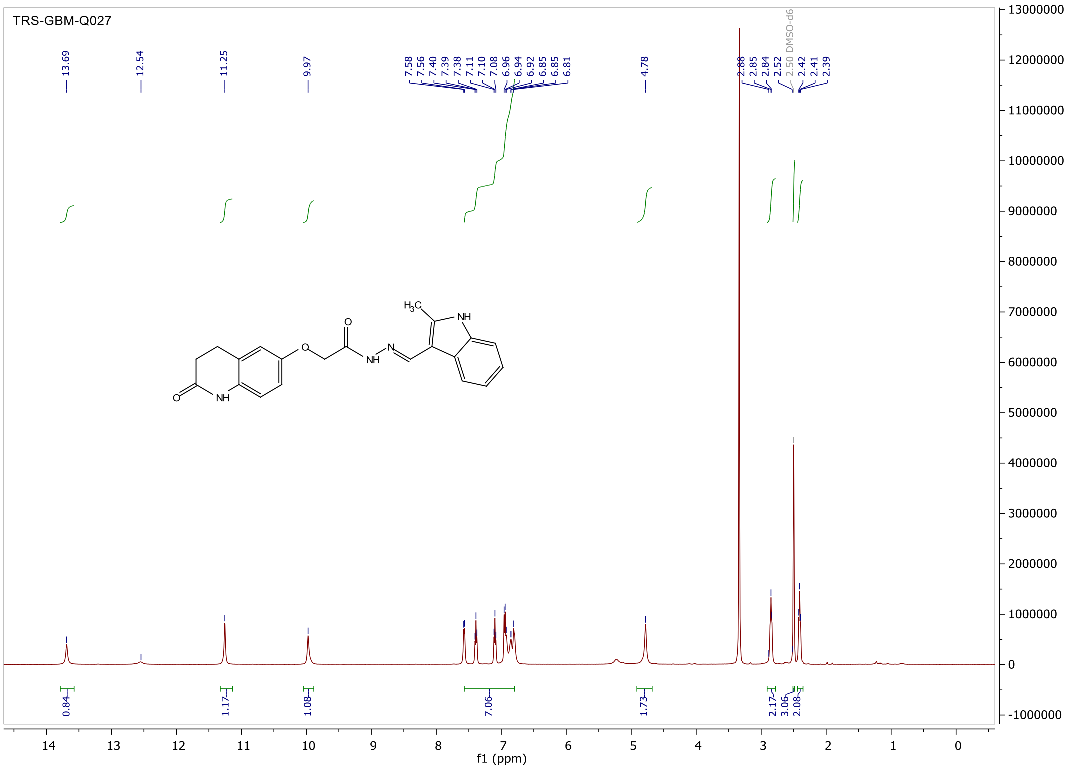

*Figure 19. ¹H-NMR of (E)-N'-((2-methyl-1H-indol-3-yl)methylene)-2-((2-oxo-1,2,3,4-tetrahydroquinolin-6-yl)oxy)acetohydrazide (**4g**)*

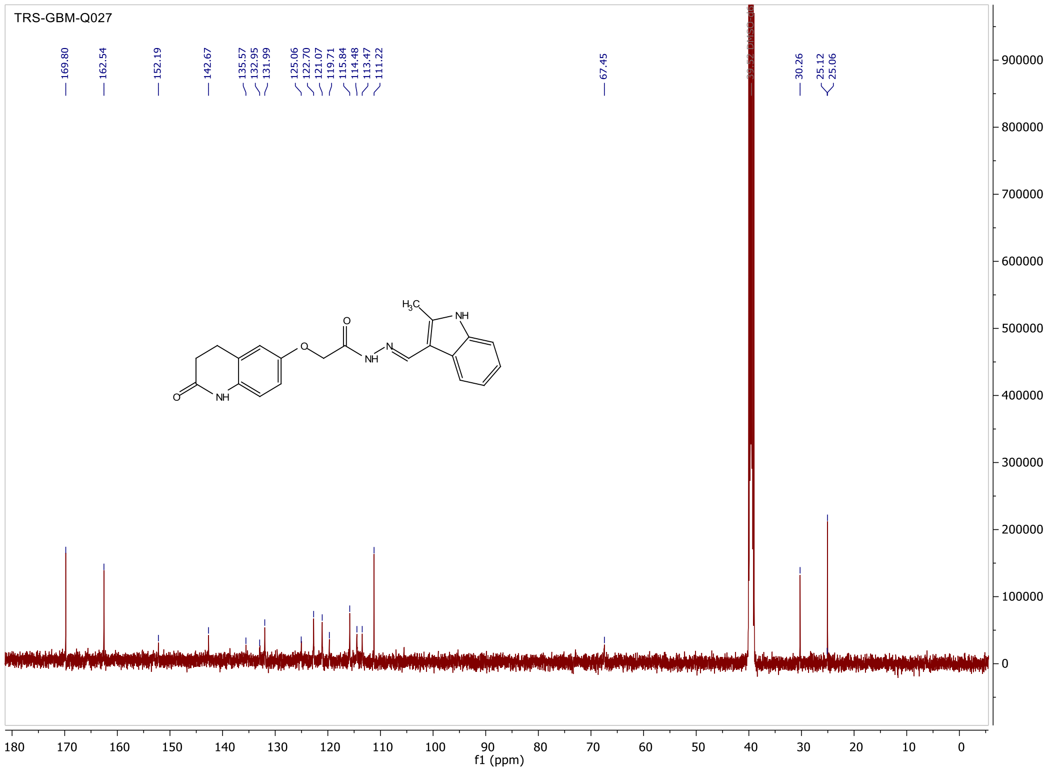

*Figure 20. ¹³C-NMR of (E)-N'-((2-methyl-1H-indol-3-yl)methylene)-2-((2-oxo-1,2,3,4-tetrahydroquinolin-6-yl)oxy)acetohydrazide (**4g**)*

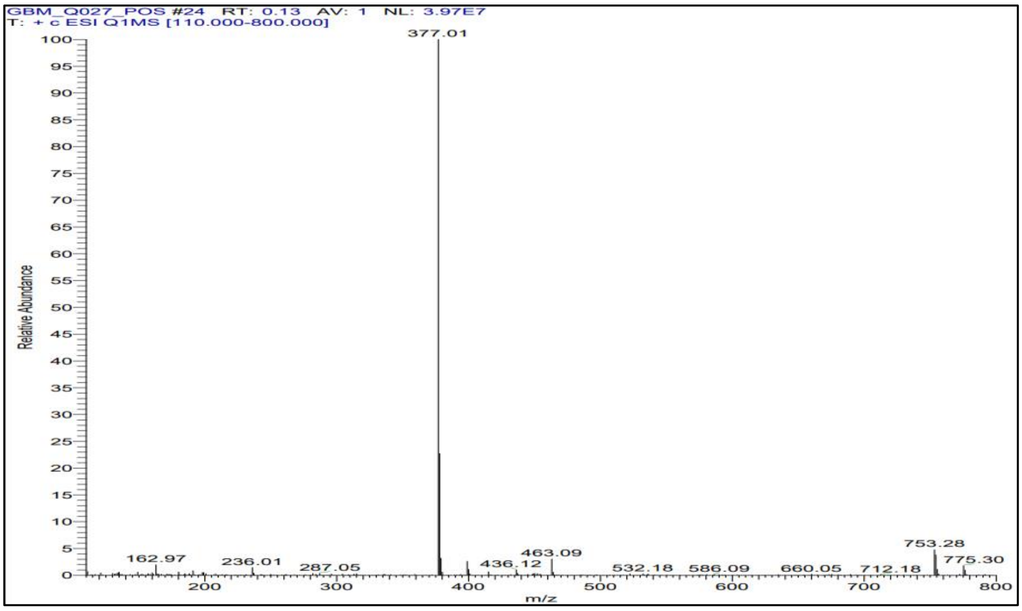

*Figure 21. Mass spectroscopy of (E)-N'-((2-methyl-1H-indol-3-yl)methylene)-2-((2-oxo-1,2,3,4-tetrahydroquinolin-6-yl)oxy)acetohydrazide (**4g**)*

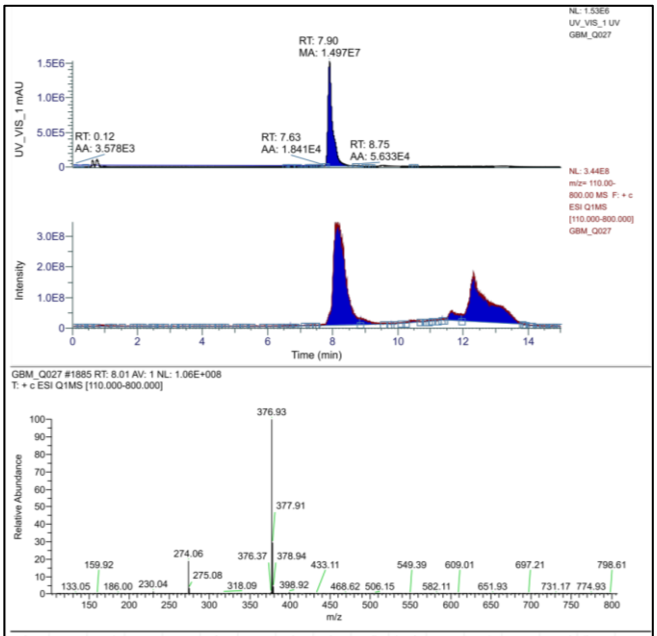

Index	Name	RT (Mi...	RT (Sec)	Start RT	End RT	Base P...	Peak...	Peak...	Baseli...	Signal...	% Area	% Hei...	Scan F...	TraceId	FileNa...	Integr...
10		7.89...	473....	7.77...	8.68...	0	1496...	1514...	0.90...	2102...	99.33	99.11	No F...	Trace1	C:\X...	Man...
7		8.75...	525....	8.68...	8.97...	0	5632...	5765...	0.28...	319....	0.37	0.38	No F...	Trace1	C:\X...	Aut...
6		7.63...	457....	7.56...	7.72...	0	1841...	3983...	0.15...	220....	0.12	0.26	No F...	Trace1	C:\X...	Aut...
5		7.49...	449....	7.38...	7.56...	0	9991...	1606...	0.17...	88.9...	0.07	0.11	No F...	Trace1	C:\X...	Aut...
3		7.06...	424....	6.92...	7.22...	0	9660...	1060...	0.30...	58.7...	0.06	0.07	No F...	Trace1	C:\X...	Aut...
1		0.11...	7.103	0.03...	0.39...	0	3578...	315....	0.355	17.4...	0.02	0.02	No F...	Trace1	C:\X...	Aut...
4		7.36...	442....	7.31...	7.38...	0	1360...	454....	0.06...	25.1...	0.01	0.03	No F...	Trace1	C:\X...	Aut...
2		6.62...	397....	6.53...	6.70...	0	532....	95.1...	0.16...	5.27...	0	0.01	No F...	Trace1	C:\X...	Aut...
8		9.12...	547....	9.09...	9.16...	0	425....	150....	0.07...	8.36...	0	0.01	No F...	Trace1	C:\X...	Aut...
9		10.4...	627....	10.4...	10.4...	0	426....	132....	0.08...	7.36...	0	0.01	No F...	Trace1	C:\X...	Aut...

*Figure 22. LCMS/MS of compound (E)-N'-((2-methyl-1H-indol-3-yl)methylene)-2-((2-oxo-1,2,3,4-tetrahydroquinolin-6-yl)oxy)acetohydrazide (**4g**) showing 99.33% purity.*

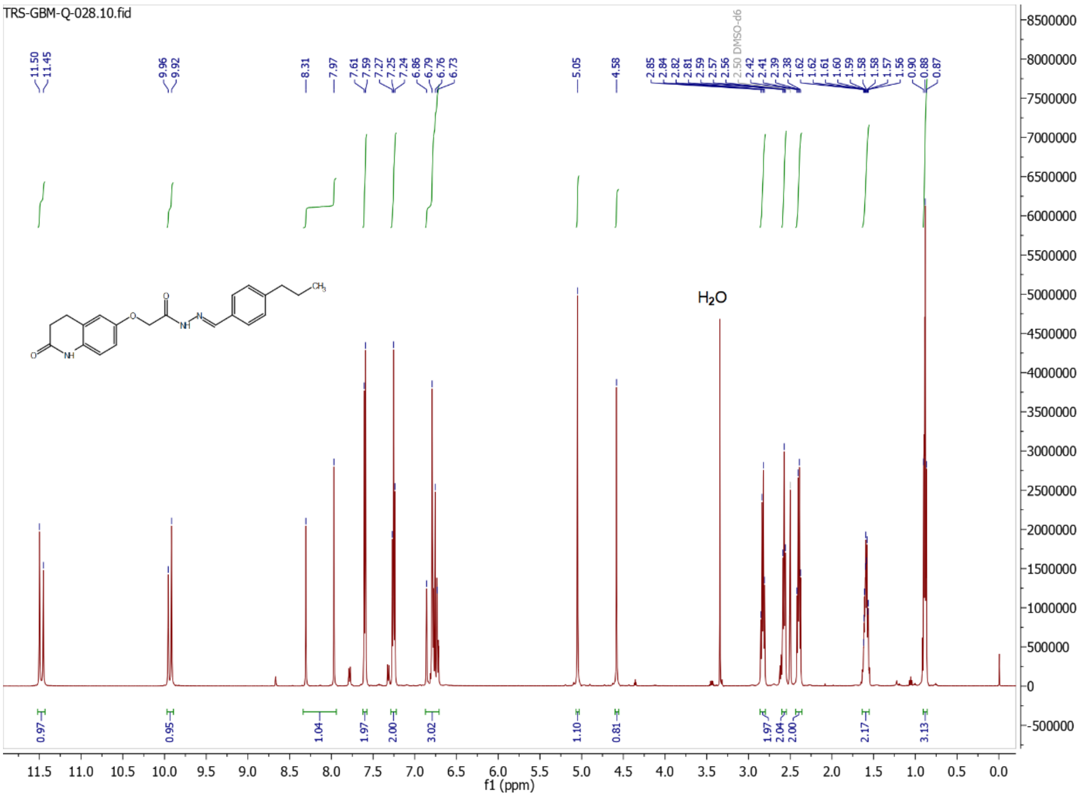

Figure 23. ^{1}H-NMR of (E)-2-((2-oxo-1,2,3,4-tetrahydroquinolin-6-yl)oxy)-N'-(4-propylbenzylidene)acetohydrazide (**4h**)

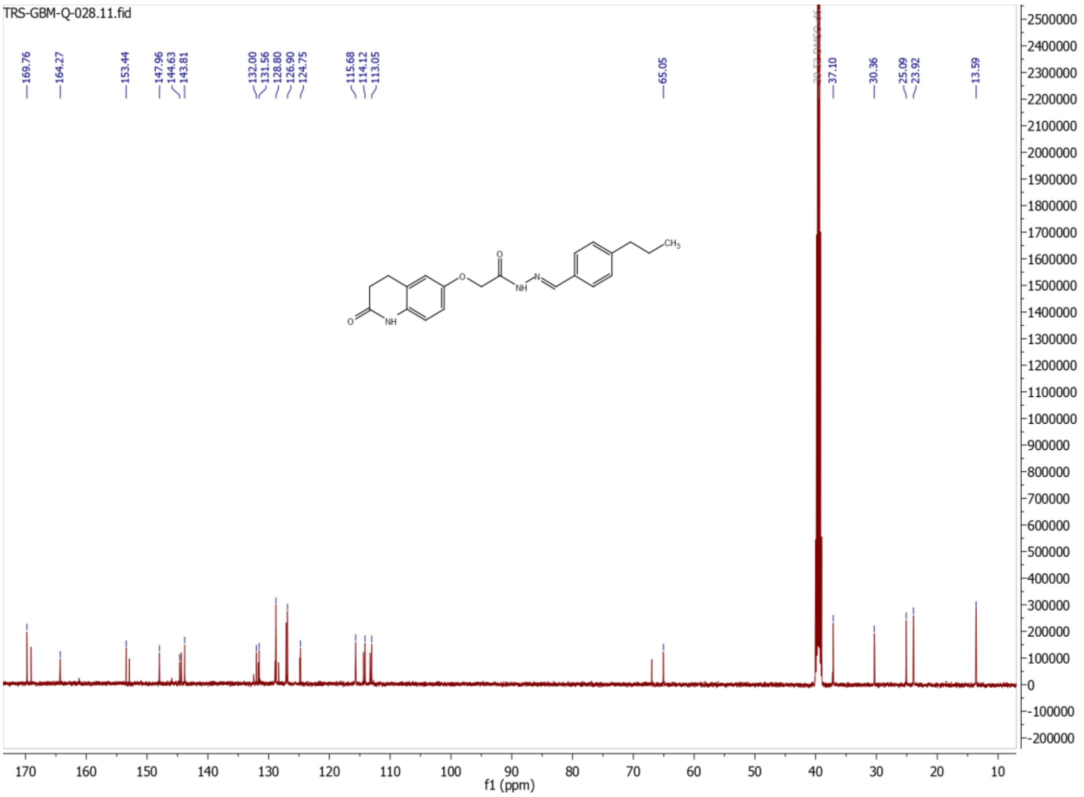

Figure 24. ^{13}C-NMR of (E)-2-((2-oxo-1,2,3,4-tetrahydroquinolin-6-yl)oxy)-N'-(4-propylbenzylidene)acetohydrazide (**4h**)

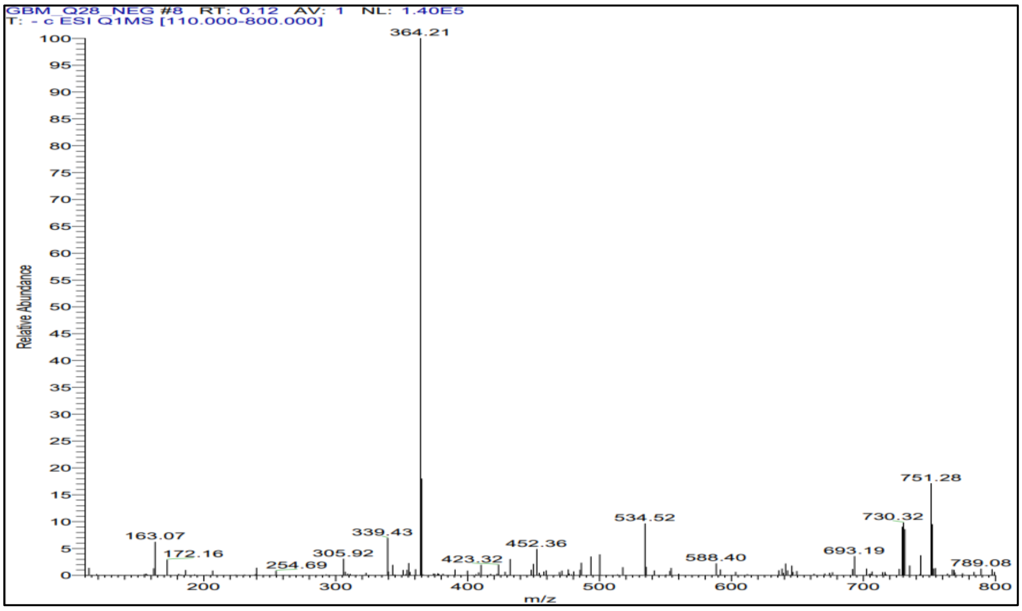

*Figure 25. Mass spectroscopy of (E)-2-((2-oxo-1,2,3,4-tetrahydroquinolin-6-yl)oxy)-N'-(4-propylbenzylidene)acetohydrazide (**4h**)*

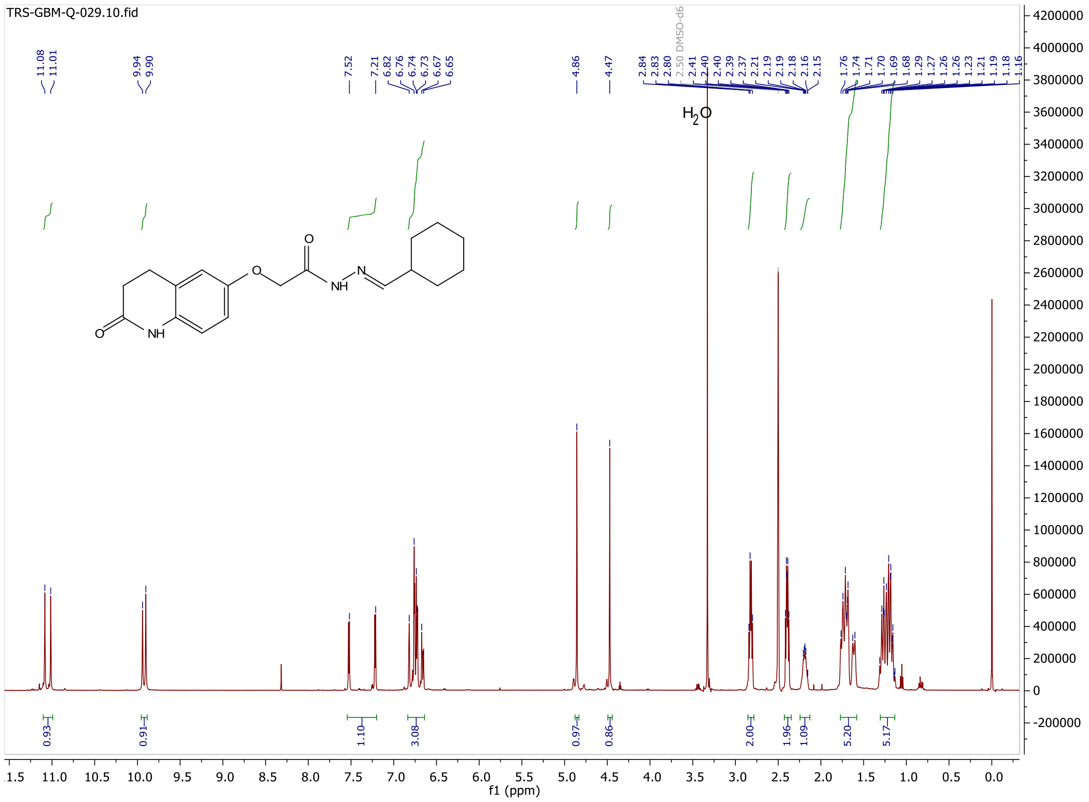

*Figure 26. ^{1}H-NMR of (E)-N'-(cyclohexylmethylene)-2-((2-oxo-1,2,3,4-tetrahydroquinolin-6-yl)oxy)acetohydrazide (**4i**)*

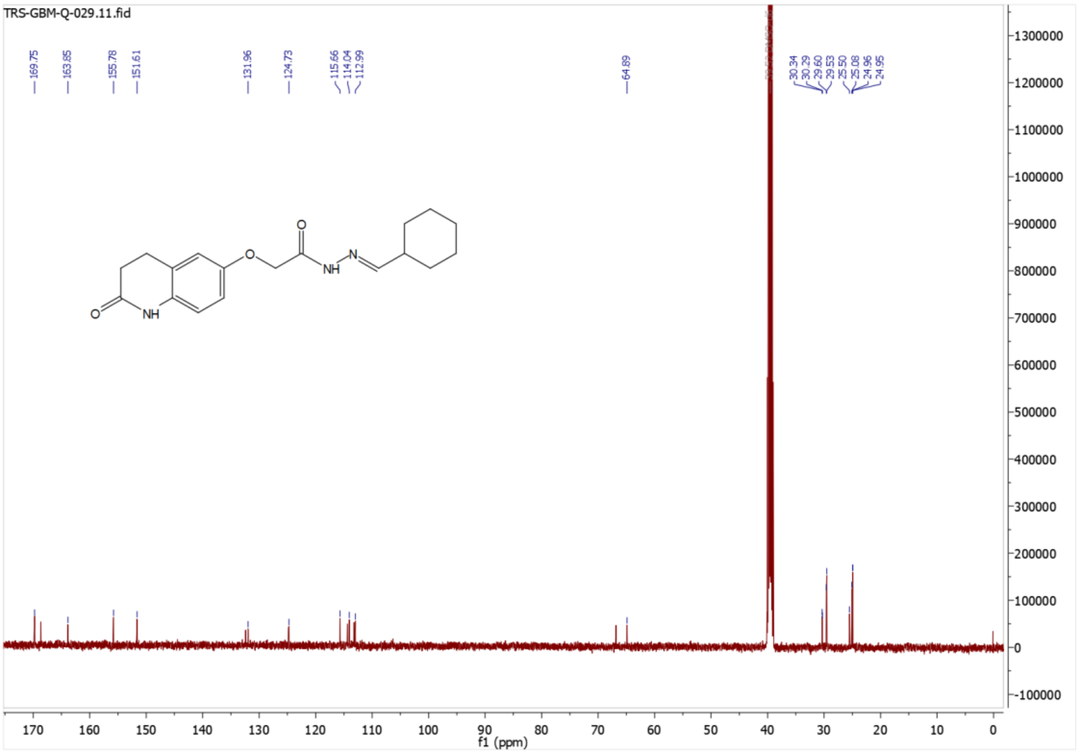

Figure 27. ^{13}*C-NMR of (E)-N'-(cyclohexylmethylene)-2-((2-oxo-1,2,3,4-tetrahydroquinolin-6-yl)oxy)acetohydrazide (**4i**)*

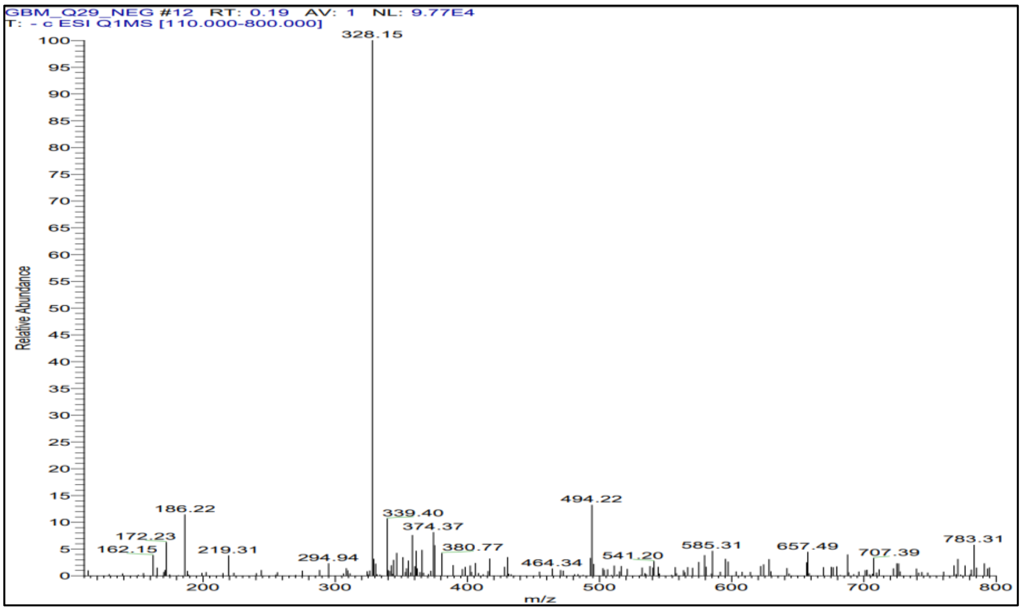

*Figure 28. Mass spectroscopy of (E)-N'-(cyclohexylmethylene)-2-((2-oxo-1,2,3,4-tetrahydroquinolin-6-yl)oxy)acetohydrazide (**4i**)*

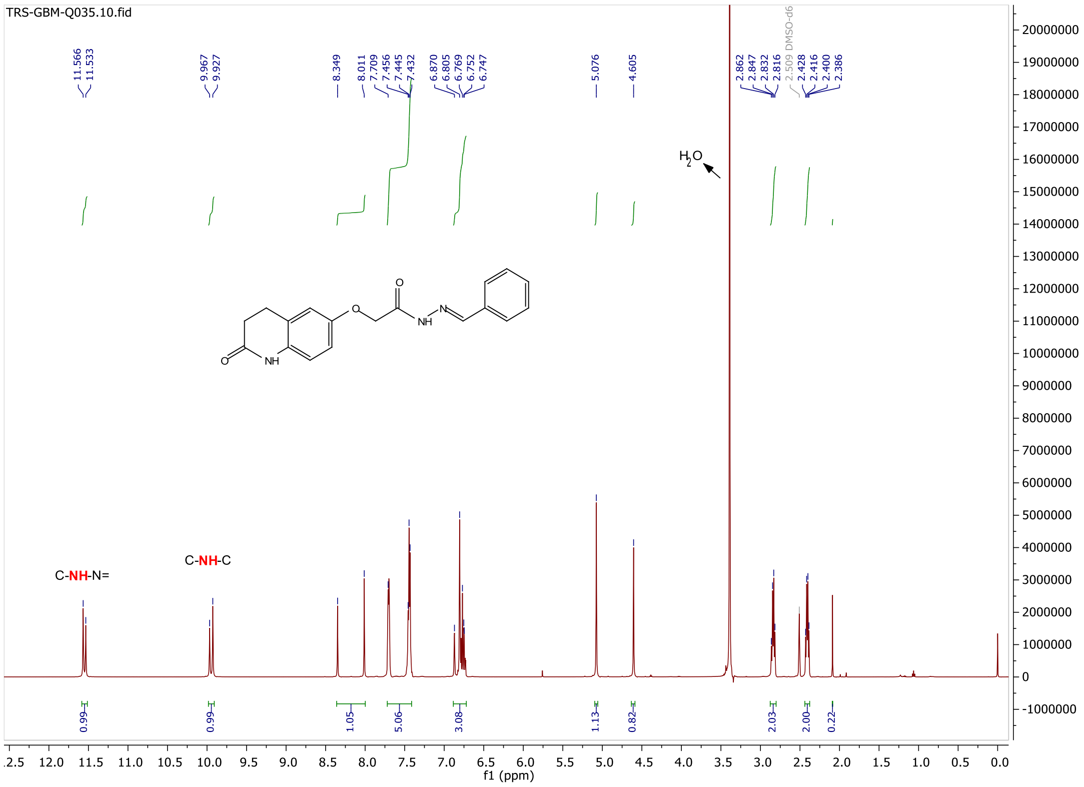

*Figure 29. ^{1}H-NMR of (E)-N'-benzylidene-2-((2-oxo-1,2,3,4-tetrahydroquinolin-6-yl)oxy)acetohydrazide (**4j**)*

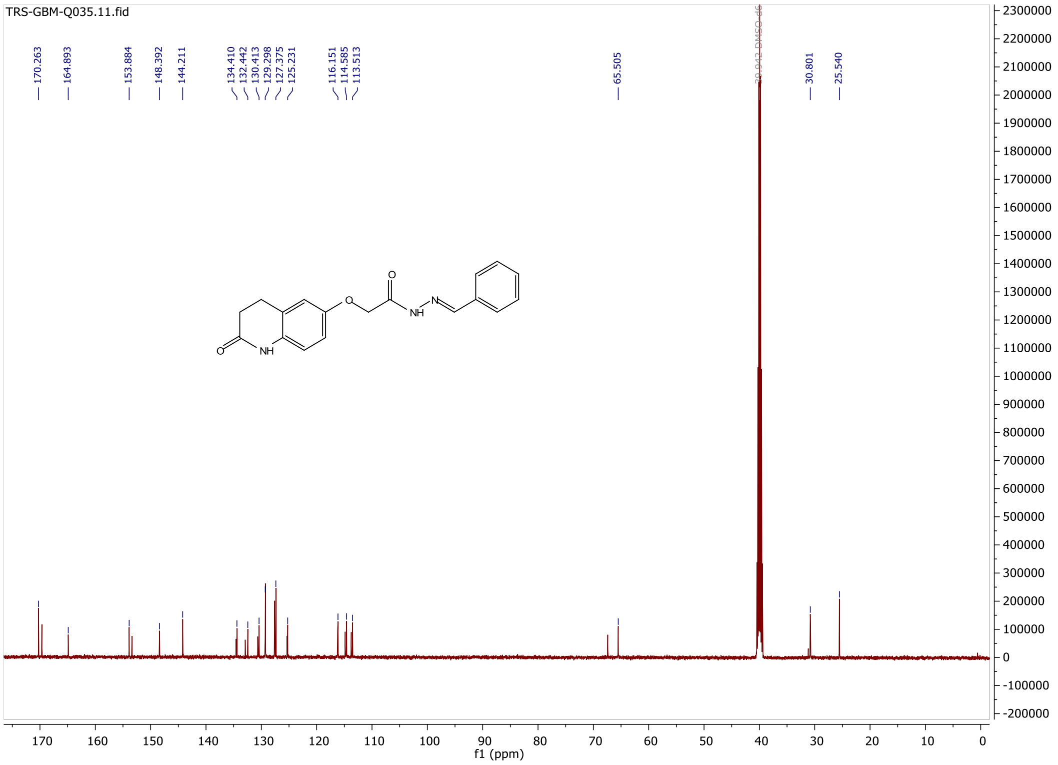

*Figure 30. ^{13}C-NMR of (E)-N'-benzylidene-2-((2-oxo-1,2,3,4-tetrahydroquinolin-6-yl)oxy)acetohydrazide (**4j**)*

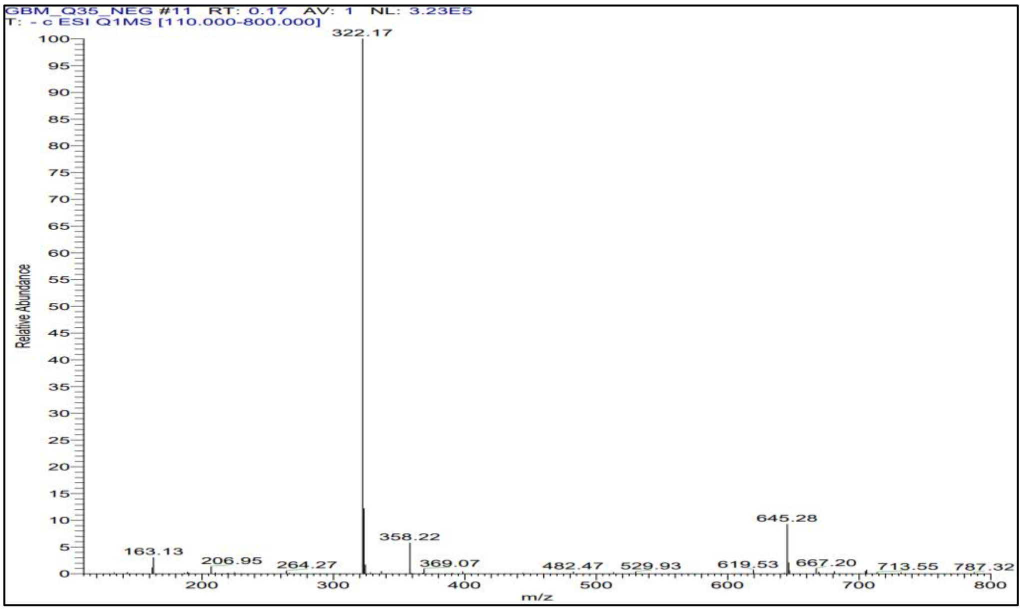

*Figure 31. Mass spectroscopy of(E)-N'-benzylidene-2-((2-oxo-1,2,3,4-tetrahydroquinolin-6-yl)oxy)acetohydrazide (**4j**)*

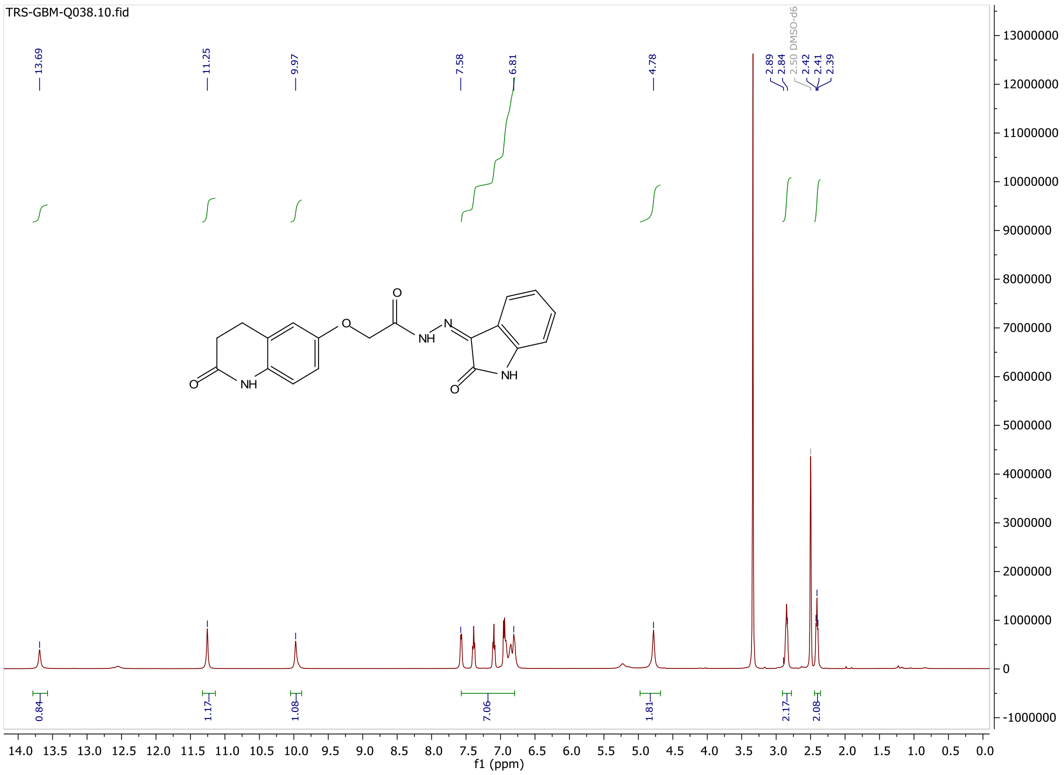

*Figure 32. ^{1}H-NMR of (Z)-2-((2-oxo-1,2,3,4-tetrahydroquinolin-6-yl)oxy)-N'-(2-oxoindolin-3-ylidene)acetohydrazide (**4k**)*

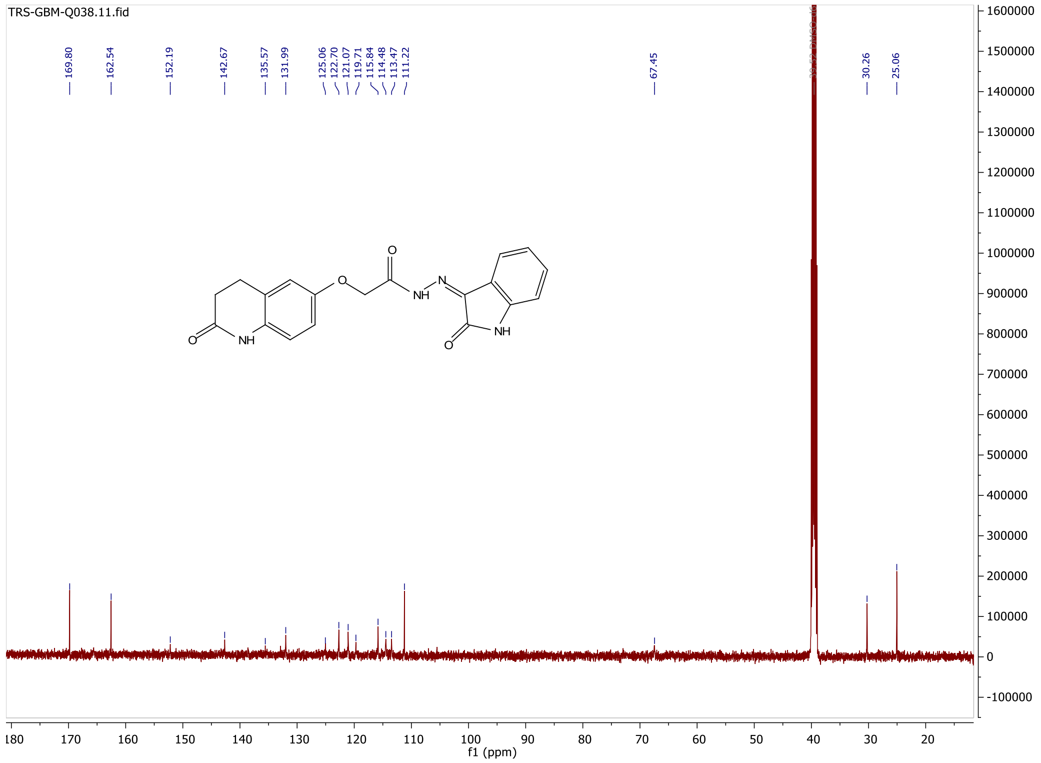

Figure 33. ^{13}C-NMR of (Z)-2-((2-oxo-1,2,3,4-tetrahydroquinolin-6-yl)oxy)-N'-(2-oxoindolin-3-ylidene)acetohydrazide (**4k**)

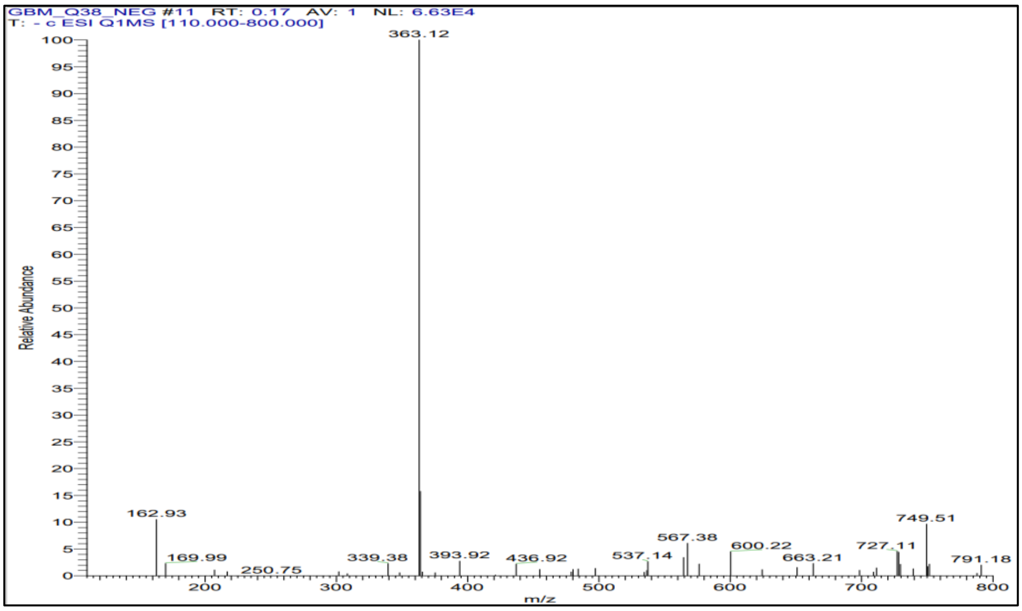

Figure 34. Mass spectroscopy of (Z)-2-((2-oxo-1,2,3,4-tetrahydroquinolin-6-yl)oxy)-N'-(2-oxoindolin-3-ylidene)acetohydrazide (**4k**)

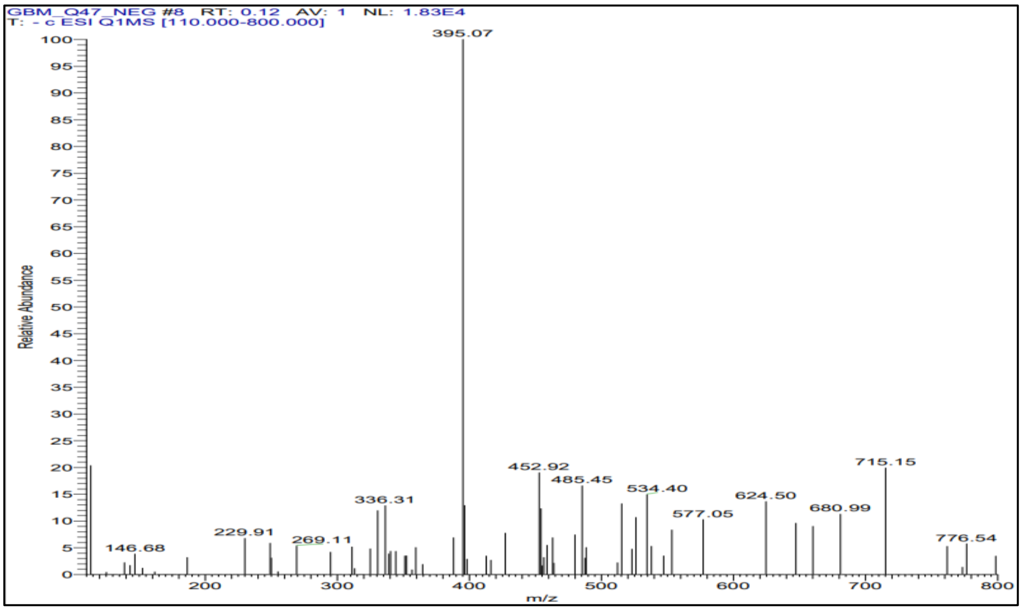

*Figure 35. Mass spectroscopy of (Z)-N'-(5-fluoro-1-methyl-2-oxoindolin-3-ylidene)-2-((2-oxo-1,2,3,4-tetrahydroquinolin-6-yl)oxy)acetohydrazide (**4l**)*

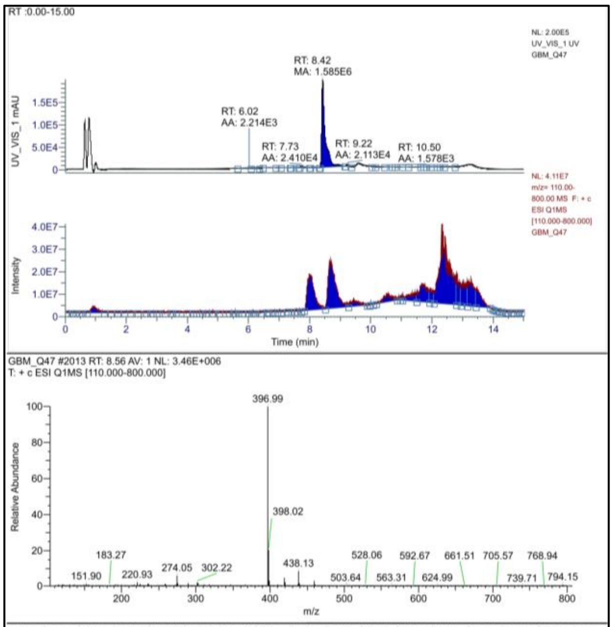

Index	Name	RT (Mi...	RT (Sec)	Start RT	End RT	Base P...	Peak...	Peak...	Baseli...	Signal...	% Area	% Hei...	Scan F...	TraceId	FileNa...	Integr...
19		8.42...	505....	8.31...	9.1334	0	1585...	1946...	0.81...	342....	95.36	94.13	No F...	Trace1	C:\X...	Man...
6		7.73...	463....	7.6584	7.9884	0	2410...	3408...	0.33	499....	1.45	1.65	No F...	Trace1	C:\X...	Aut...
8		9.2234	553....	9.1584	9.35...	0	2112...	4441...	0.19...	650....	1.27	2.15	No F...	Trace1	C:\X...	Aut...
7		8.1034	486....	8.01...	8.3034	0	9833...	1528...	0.28...	223....	0.59	0.74	No F...	Trace1	C:\X...	Aut...
3		6.6834	401....	6.45...	6.87...	0	6286...	529....	0.42...	77.5...	0.38	0.26	No F...	Trace1	C:\X...	Aut...
5		7.4634	447....	7.3684	7.62...	0	2845...	416....	0.25...	61.0...	0.17	0.2	No F...	Trace1	C:\X...	Aut...
1		6.02...	361....	5.62...	6.05...	0	2214...	143....	0.43	20.9...	0.13	0.07	No F...	Trace1	C:\X...	Aut...
18		12.5...	754....	12.3...	12.7...	0	1790...	138....	0.32...	20.2...	0.11	0.07	No F...	Trace1	C:\X...	Aut...
2		6.07...	364....	6.05...	6.32...	0	1421...	140....	0.27...	20.6...	0.09	0.07	No F...	Trace1	C:\X...	Aut...
10		10.5...	630....	10.4...	10.6...	0	1577...	266....	0.22...	39.0...	0.09	0.13	No F...	Trace1	C:\X...	Aut...

*Figure 36. LCMS/MS of compound (Z)-N'-(5-fluoro-1-methyl-2-oxoindolin-3-ylidene)-2-((2-oxo-1,2,3,4-tetrahydroquinolin-6-yl)oxy)acetohydrazide (**4l**) showing 95.36% purity.*

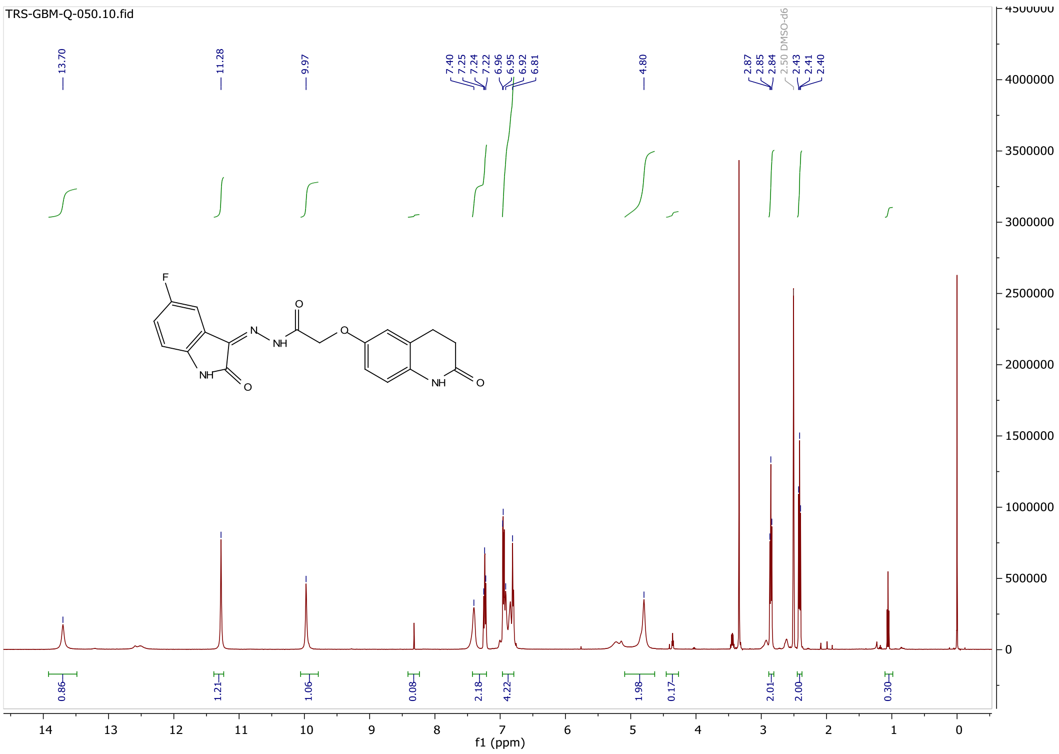

*Figure 37. ¹H-NMR of (Z)-N'-(5-fluoro-2-oxoindolin-3-ylidene)-2-((2-oxo-1,2,3,4-tetrahydroquinolin-6-yl)oxy)acetohydrazide (**4m**)*

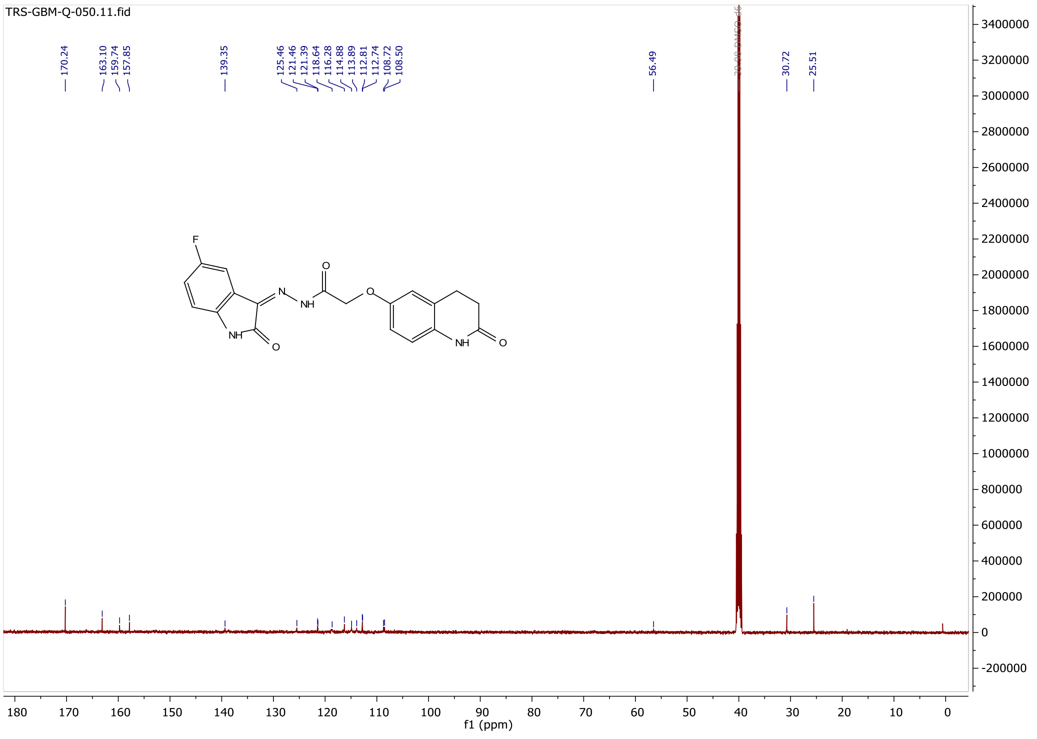

*Figure 38. ¹³C-NMR of (Z)-N'-(5-fluoro-2-oxoindolin-3-ylidene)-2-((2-oxo-1,2,3,4-tetrahydroquinolin-6-yl)oxy)acetohydrazide (**4m**)*

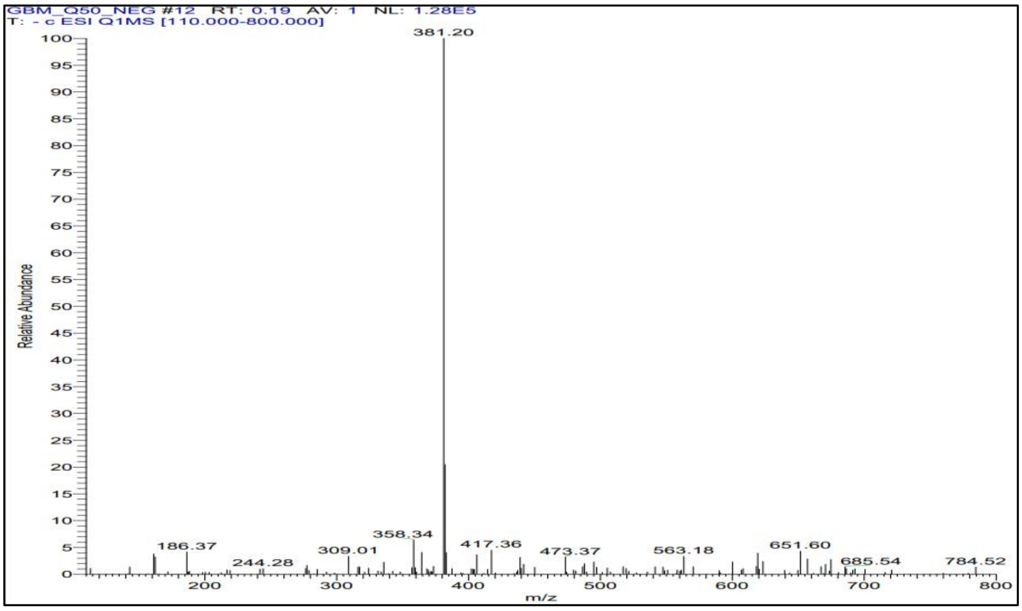

*Figure 39. Mass spectroscopy of (Z)-N'-(5-fluoro-2-oxoindolin-3-ylidene)-2-((2-oxo-1,2,3,4-tetrahydroquinolin-6-yl)oxy)acetohydrazide (**4m**)*

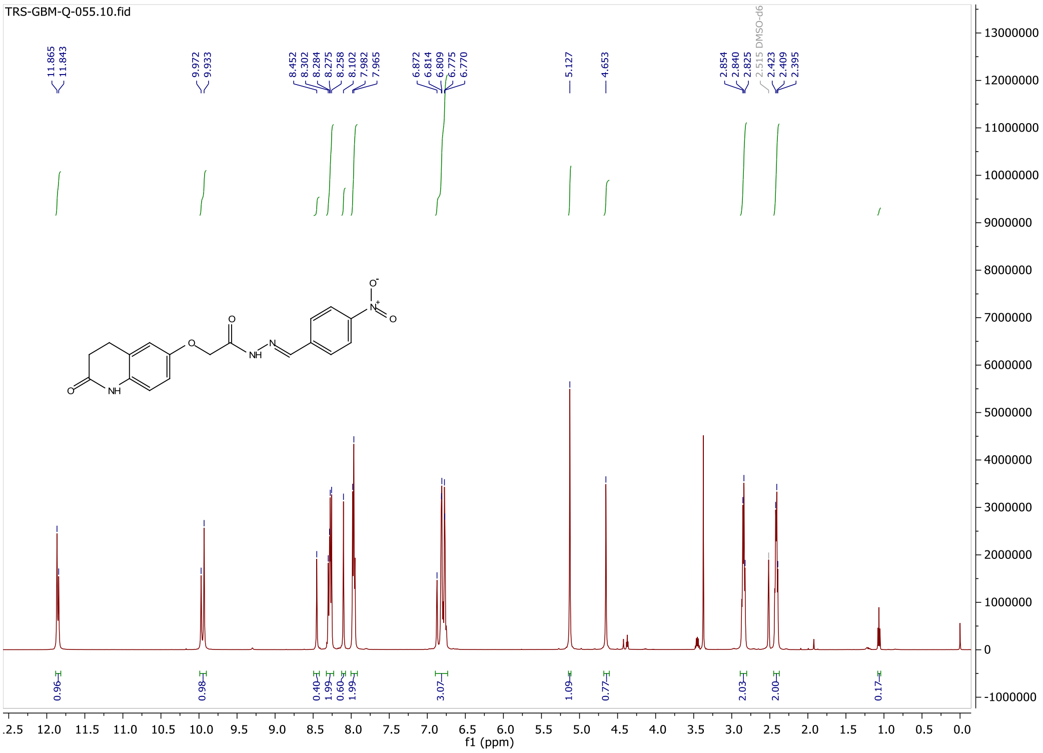

*Figure 40. ^{1}H-NMR of (E)-N'-(4-nitrobenzylidene)-2-((2-oxo-1,2,3,4-tetrahydroquinolin-6-yl)oxy)acetohydrazide (**4n**)*

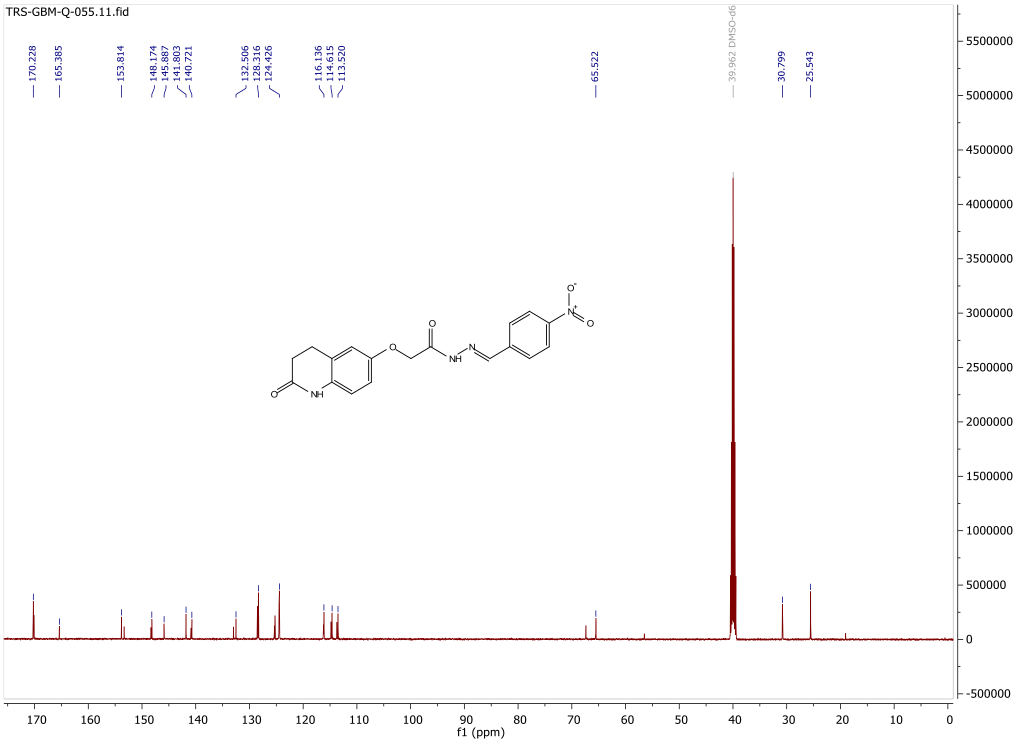

*Figure 41. ^{13}C-NMR of (E)-N'-(4-nitrobenzylidene)-2-((2-oxo-1,2,3,4-tetrahydroquinolin-6-yl)oxy)acetohydrazide (**4n**)*

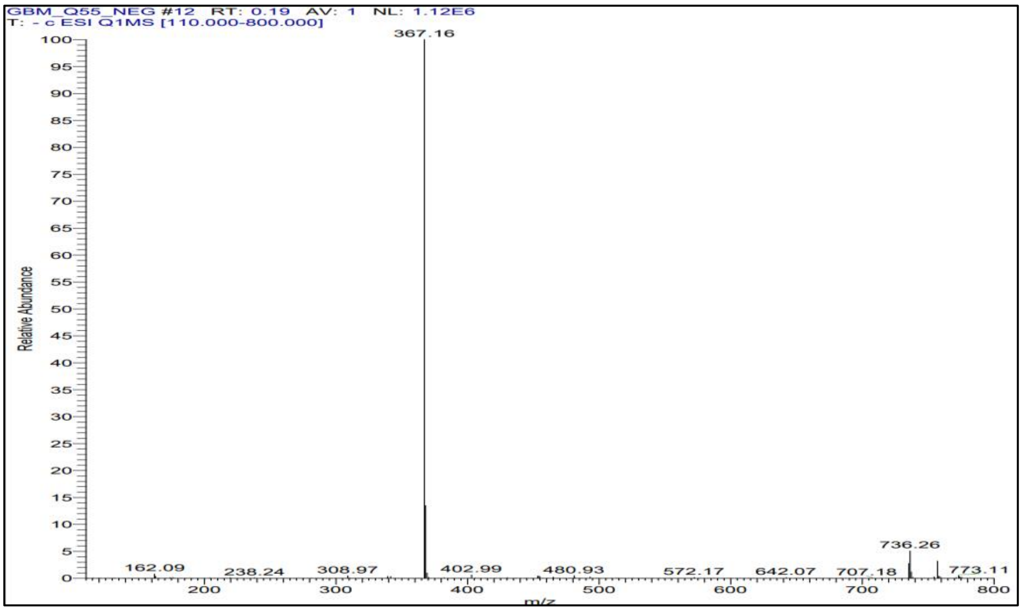

*Figure 42. Mass spectroscopy of (E)-N'-(4-nitrobenzylidene)-2-((2-oxo-1,2,3,4-tetrahydroquinolin-6-yl)oxy)acetohydrazide (**4n**)*

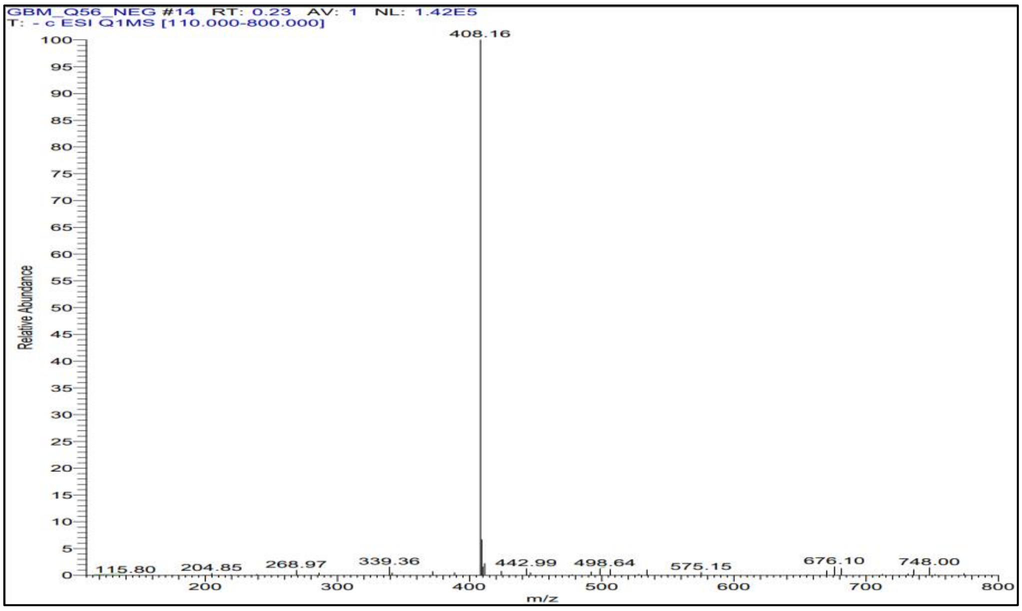

*Figure 43. Mass spectroscopy of (Z)-N'-(5-nitro-2-oxoindolin-3-ylidene)-2-((2-oxo-1,2,3,4-tetrahydroquinolin-6-yl)oxy)acetohydrazide (**4o**)*

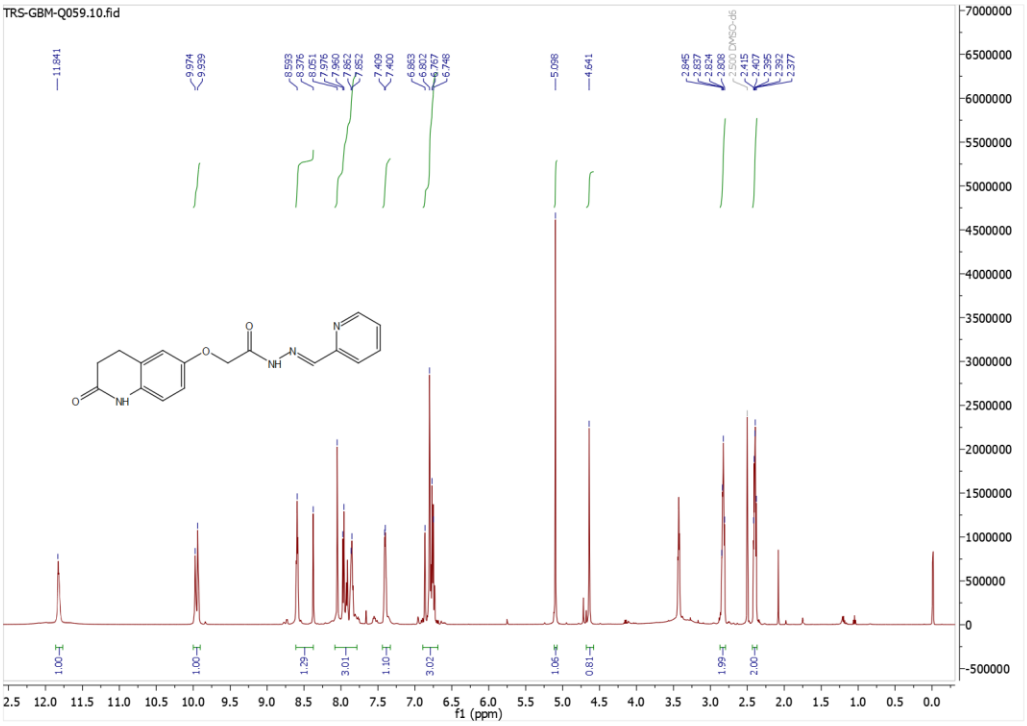

*Figure 44. ^{1}H-NMR of (E)-2-((2-oxo-1,2,3,4-tetrahydroquinolin-6-yl)oxy)-N'-(pyridin-2-ylmethylene)acetohydrazide (**4p**)*

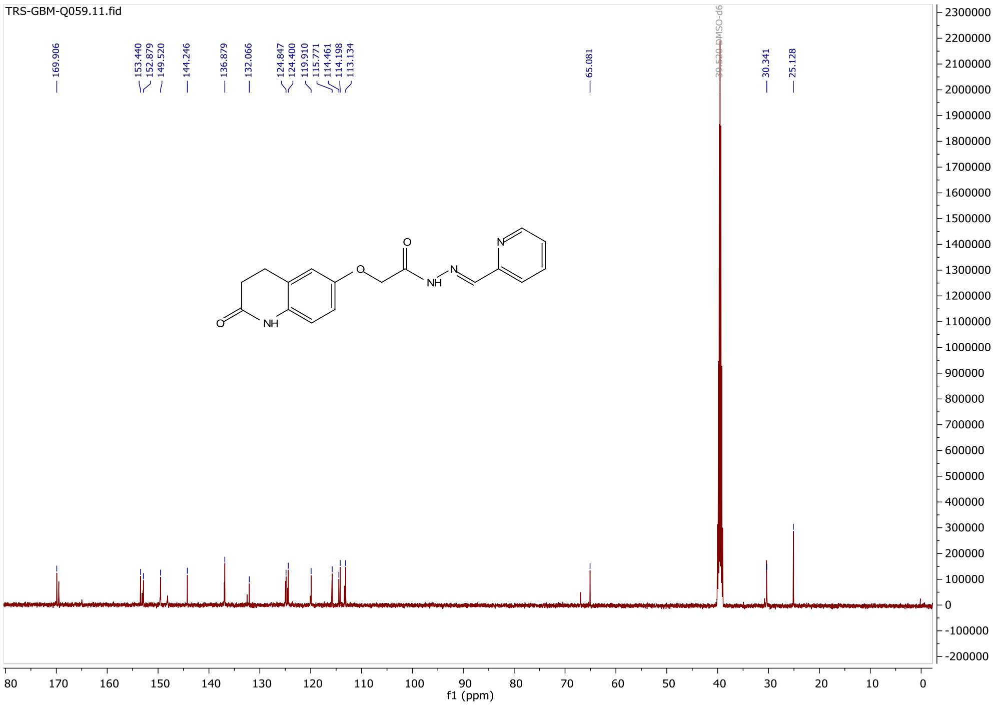

*Figure 45. ^{13}C-NMR of (E)-2-((2-oxo-1,2,3,4-tetrahydroquinolin-6-yl)oxy)-N'-(pyridin-2-ylmethylene)acetohydrazide (**4p**)*

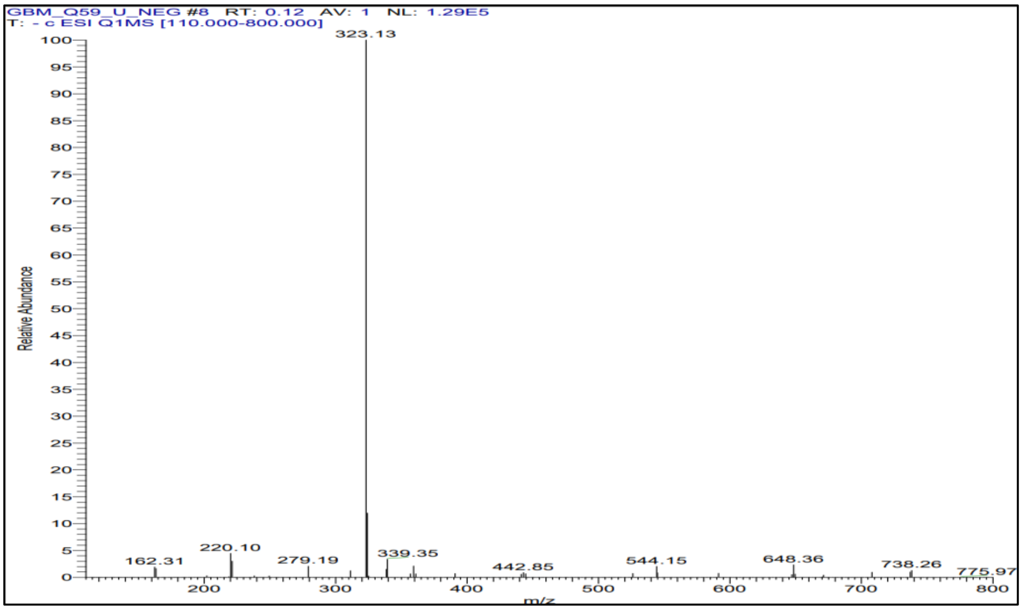

*Figure 46. Mass spectroscopy of (E)-2-((2-oxo-1,2,3,4-tetrahydroquinolin-6-yl)oxy)-N'-(pyridin-2-ylmethylene)acetohydrazide (**4p**)*

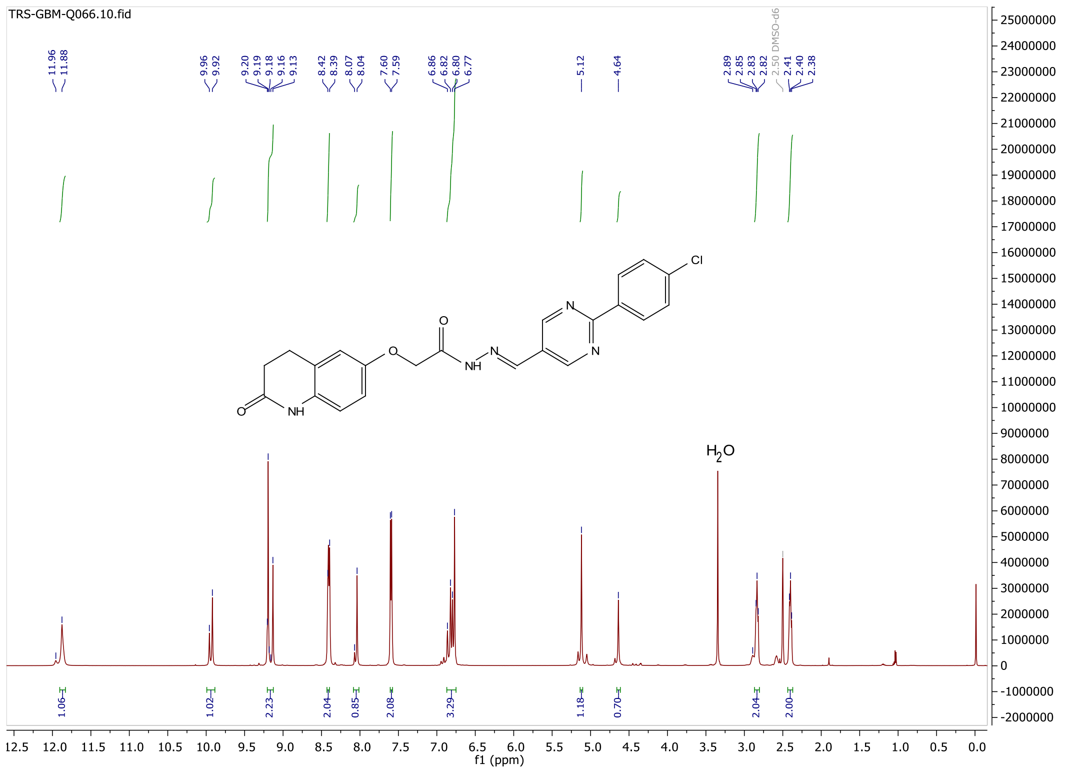

*Figure 47. ^{1}H-NMR of (E)-N'-((2-(4-chlorophenyl)pyrimidin-5-yl)methylene)-2-((2-oxo-1,2,3,4-tetrahydroquinolin-6-yl)oxy)acetohydrazide (**4q**)*

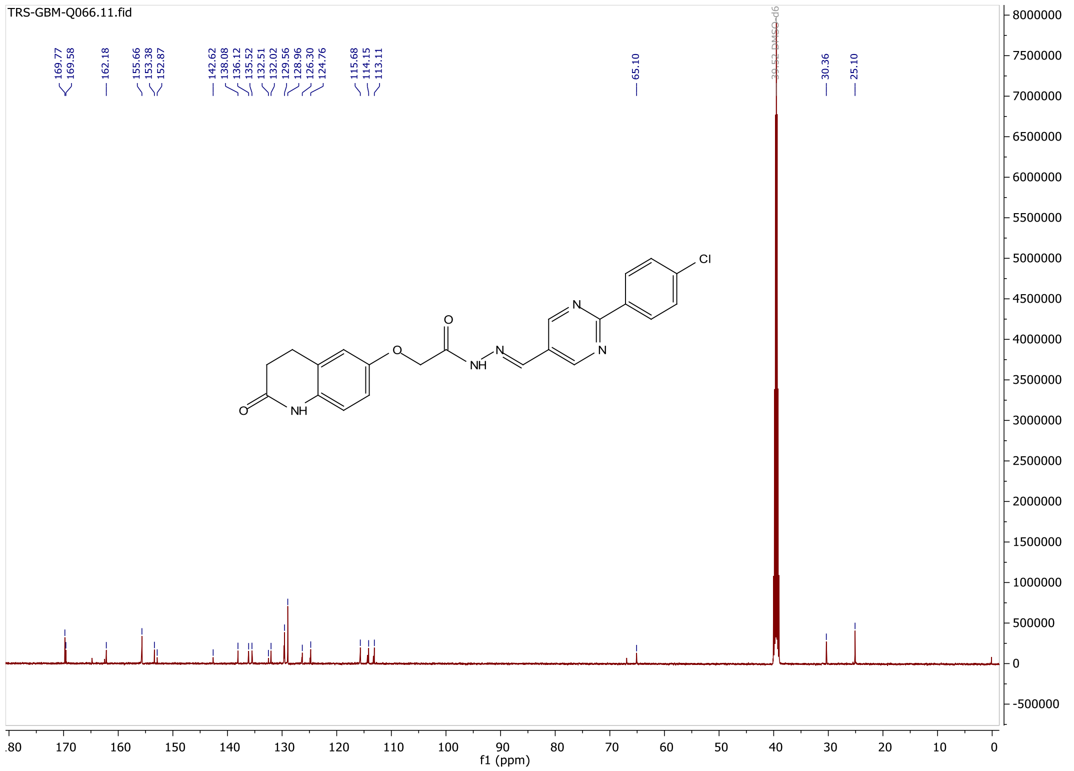

*Figure 48. ^{13}C-NMR of (E)-N'-((2-(4-chlorophenyl)pyrimidin-5-yl)methylene)-2-((2-oxo-1,2,3,4-tetrahydroquinolin-6-yl)oxy)acetohydrazide (**4q**)*

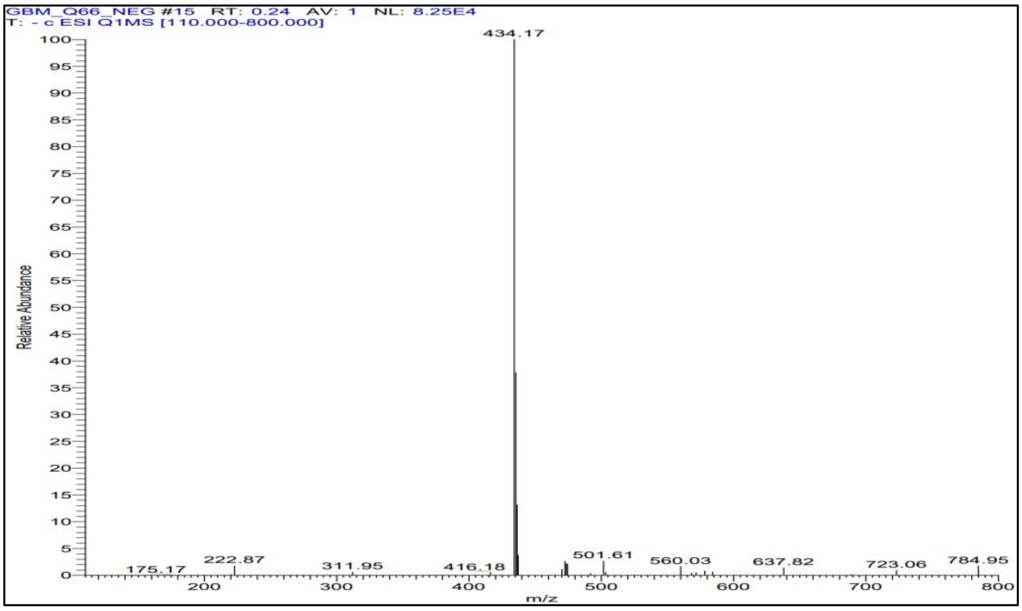

*Figure 49. Mass spectroscopy of (E)-N'-((2-(4-chlorophenyl)pyrimidin-5-yl)methylene)-2-((2-oxo-1,2,3,4-tetrahydroquinolin-6-yl)oxy)acetohydrazide (**4q**)*

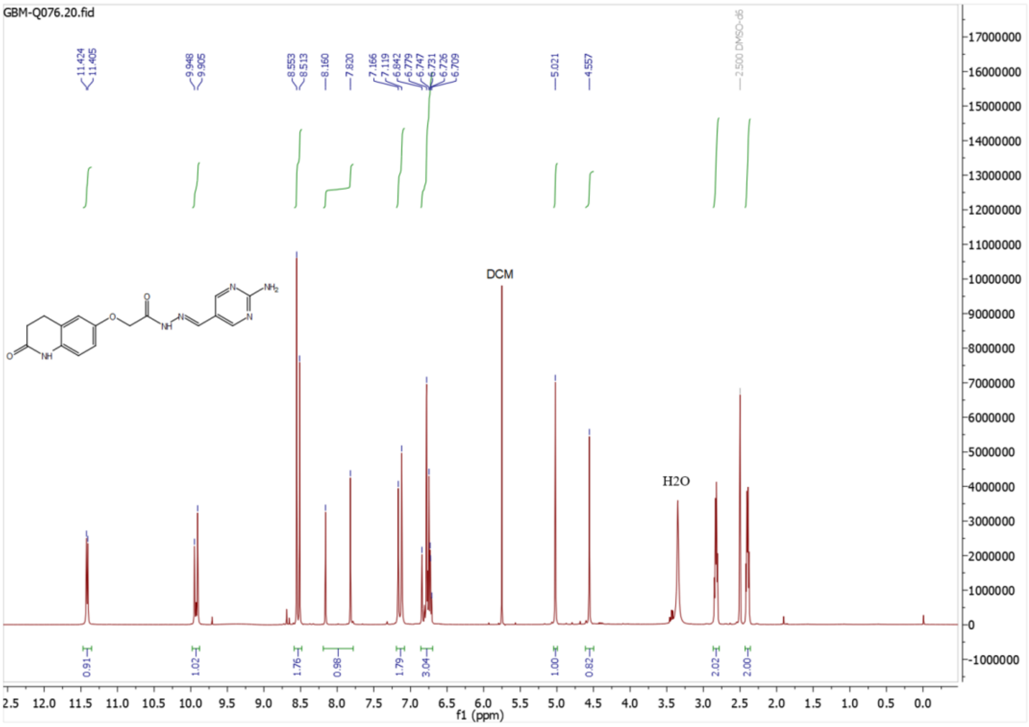

*Figure 50. ^{1}H-NMR of (E)-N'-((2-aminopyrimidin-5-yl)methylene)-2-((2-oxo-1,2,3,4-tetrahydroquinolin-6-yl)oxy)acetohydrazide (**4r**)*

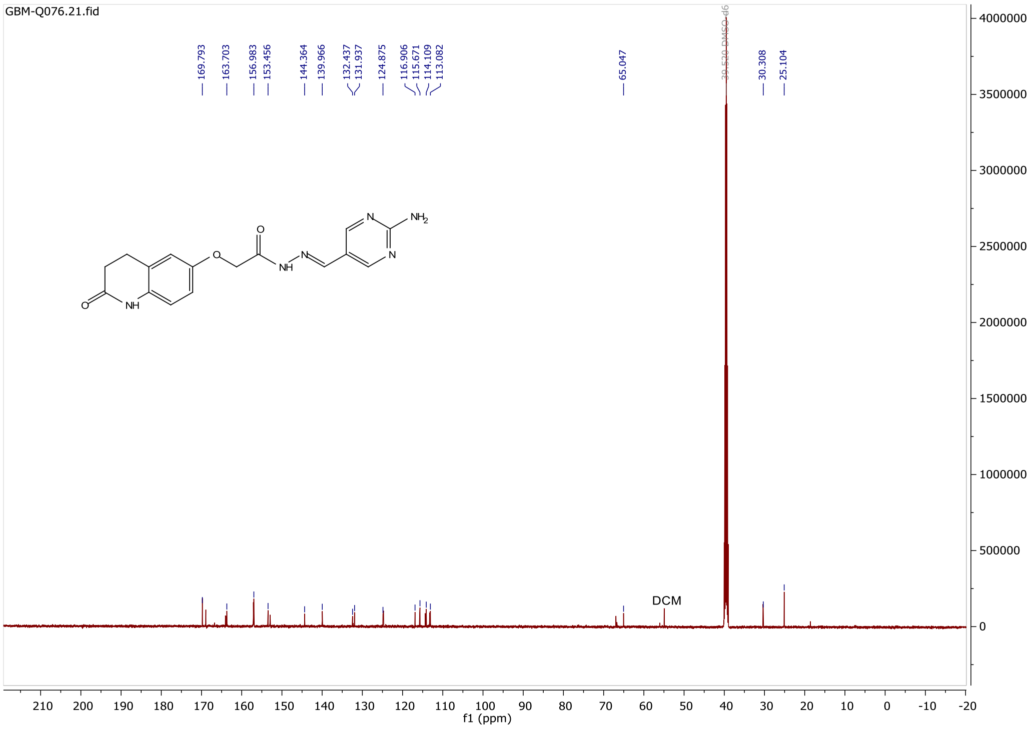

*Figure 51. ^{13}C-NMR of (E)-N'-((2-aminopyrimidin-5-yl)methylene)-2-((2-oxo-1,2,3,4-tetrahydroquinolin-6-yl)oxy)acetohydrazide (**4r**)*

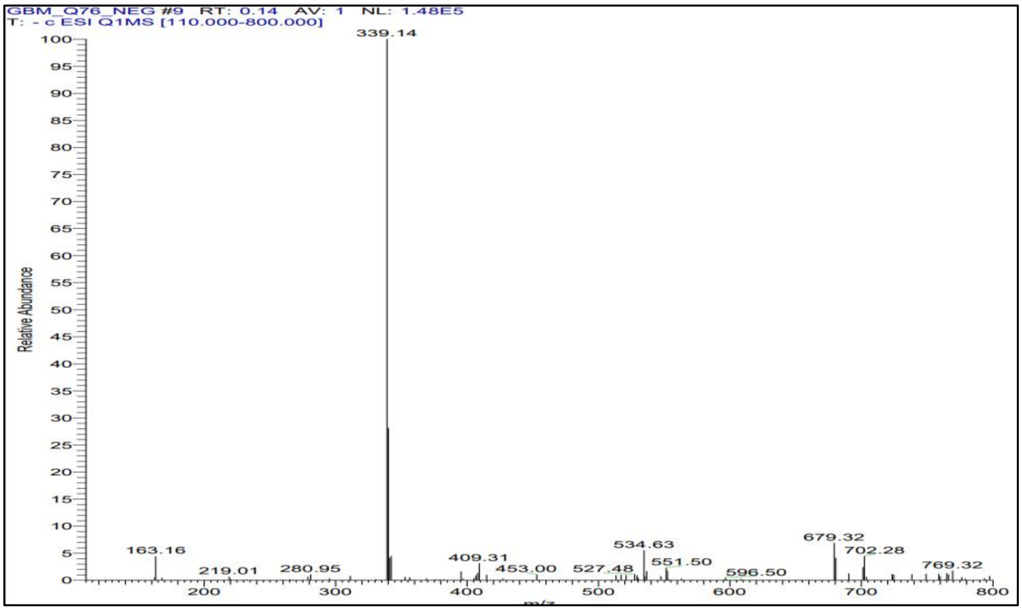

*Figure 52. Mass spectroscopy of (E)-N'-((2-aminopyrimidin-5-yl)methylene)-2-((2-oxo-1,2,3,4-tetrahydroquinolin-6-yl)oxy)acetohydrazide (**4r**)*

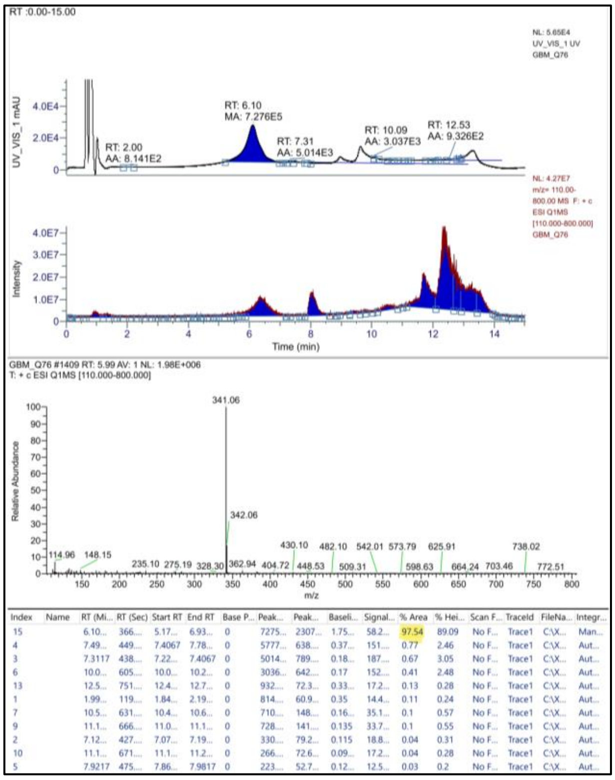

Index	Name	RT (Mi...	RT (Sec)	Start RT	End RT	Base P...	Peak...	Peak...	Baseli...	Signal...	% Area	% Hei...	Scan F...	TraceId	FileNa...	Integr...
15		6.10...	366....	5.17...	6.93...	0	7275...	2307...	1.75...	58.2...	97.54	89.09	No F...	Trace1	C:\X...	Man...
4		7.49...	449....	7.4067	7.78...	0	5777...	638....	0.37...	151....	0.77	2.46	No F...	Trace1	C:\X...	Aut...
3		7.3117	438....	7.22...	7.4067	0	5014...	789....	0.18...	187....	0.67	3.05	No F...	Trace1	C:\X...	Aut...
6		10.0...	605....	10.0...	10.2...	0	3036...	642....	0.17	152....	0.41	2.48	No F...	Trace1	C:\X...	Aut...
13		12.5...	751....	12.4...	12.7...	0	932....	72.3...	0.33...	17.2...	0.13	0.28	No F...	Trace1	C:\X...	Aut...
1		1.99...	119....	1.84...	2.19...	0	814....	60.9...	0.35	14.4...	0.11	0.24	No F...	Trace1	C:\X...	Aut...
7		10.5...	631....	10.4...	10.6...	0	710....	148....	0.16...	35.1...	0.1	0.57	No F...	Trace1	C:\X...	Aut...
9		11.1...	666....	11.0...	11.1...	0	728....	141....	0.135	33.7...	0.1	0.55	No F...	Trace1	C:\X...	Aut...
2		7.12...	427....	7.07...	7.19...	0	330....	79.2...	0.115	18.8...	0.04	0.31	No F...	Trace1	C:\X...	Aut...
10		11.1...	671....	11.1...	11.2...	0	266....	72.6...	0.09...	17.2...	0.04	0.28	No F...	Trace1	C:\X...	Aut...
5		7.9217	475....	7.86...	7.9817	0	223....	52.7...	0.12...	12.5...	0.03	0.2	No F...	Trace1	C:\X...	Aut...

*Figure 53. LCMS/MS of compound (E)-N'-((2-aminopyrimidin-5-yl)methylene)-2-((2-oxo-1,2,3,4-tetrahydroquinolin-6-yl)oxy)acetohydrazide (**4r**) showing 97.54% purity.*

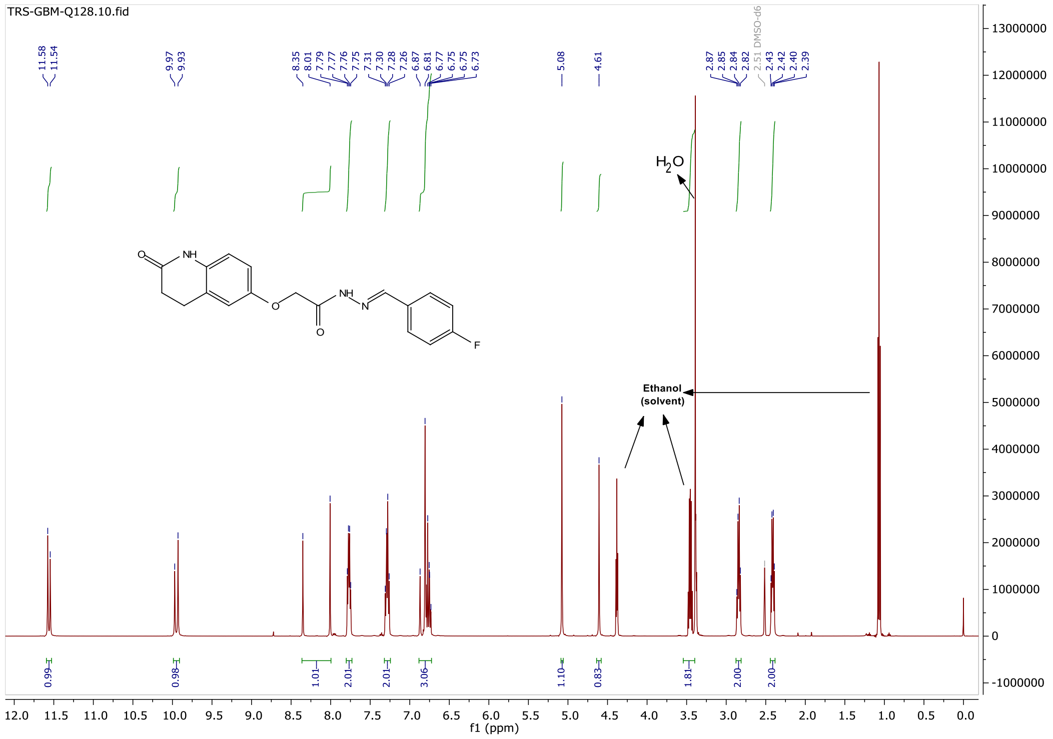

*Figure 54. ^{1}H-NMR of (E)-N'-(4-fluorobenzylidene)-2-((2-oxo-1,2,3,4-tetrahydroquinolin-6-yl)oxy)acetohydrazide (**4s**)*

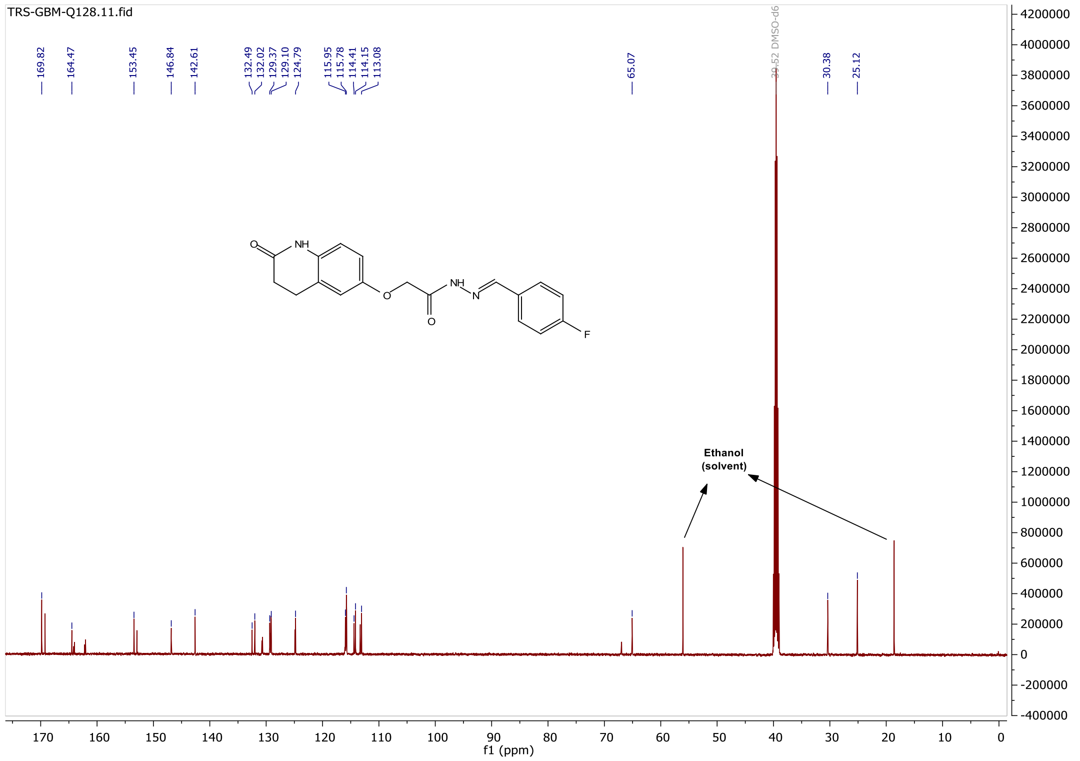

*Figure 55. ^{13}C-NMR of (E)-N'-(4-fluorobenzylidene)-2-((2-oxo-1,2,3,4-tetrahydroquinolin-6-yl)oxy)acetohydrazide (**4s**)*

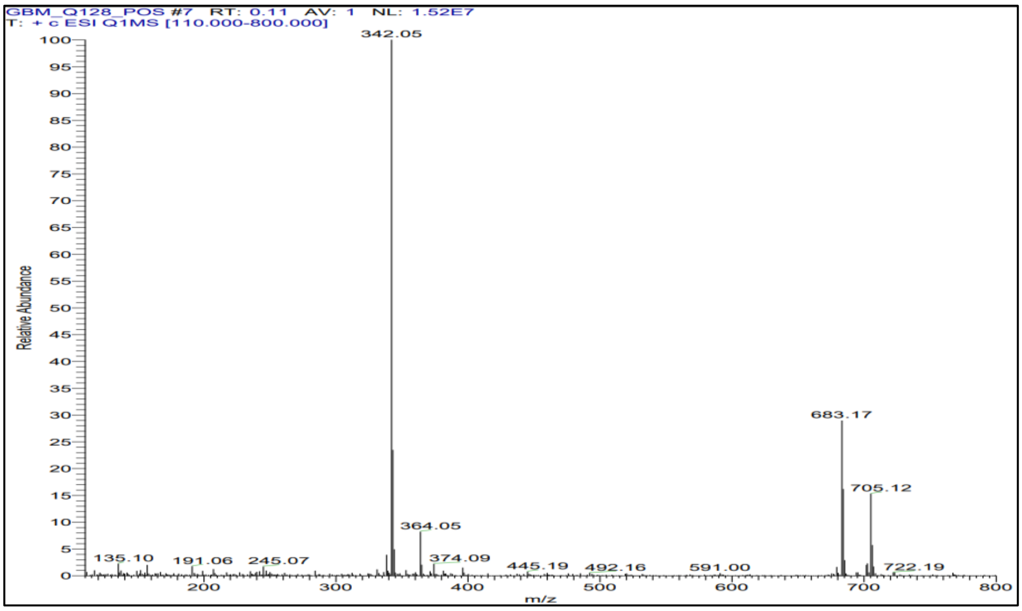

*Figure 56. Mass spectroscopy of (E)-N'-(4-fluorobenzylidene)-2-((2-oxo-1,2,3,4-tetrahydroquinolin-6-yl)oxy)acetohydrazide (**4s**)*

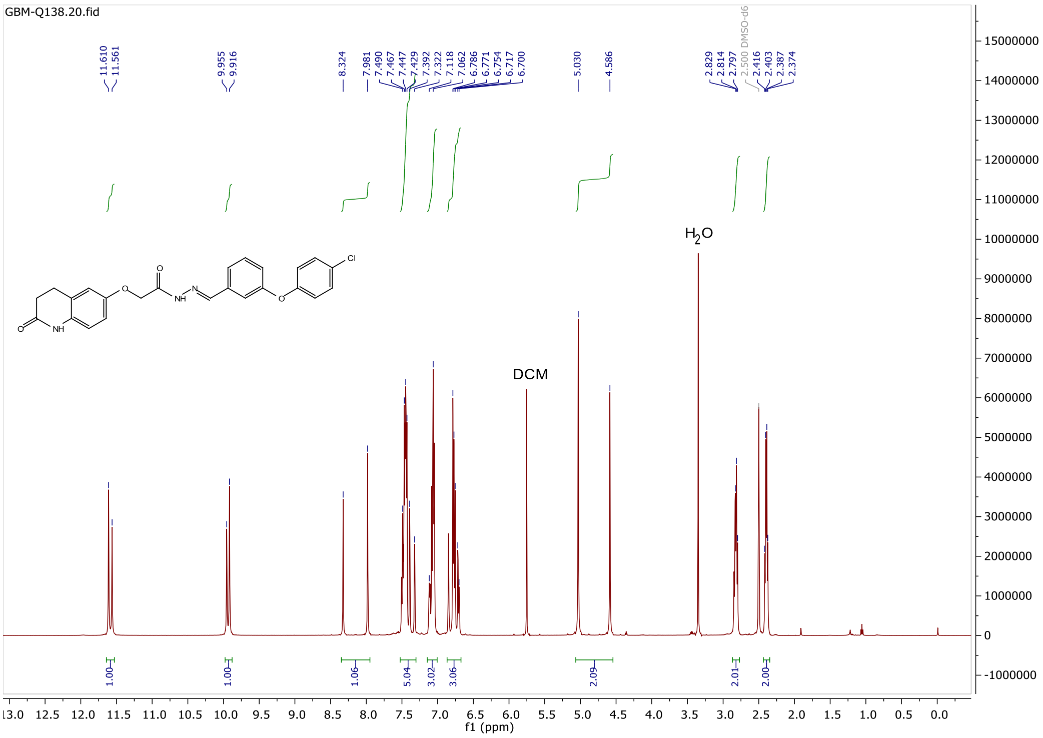

*Figure 57. ^{1}H-NMR of (E)-N'-(3-(4-chlorophenoxy)benzylidene)-2-((2-oxo-1,2,3,4-tetrahydroquinolin-6-yl)oxy)acetohydrazide (**4t**)*

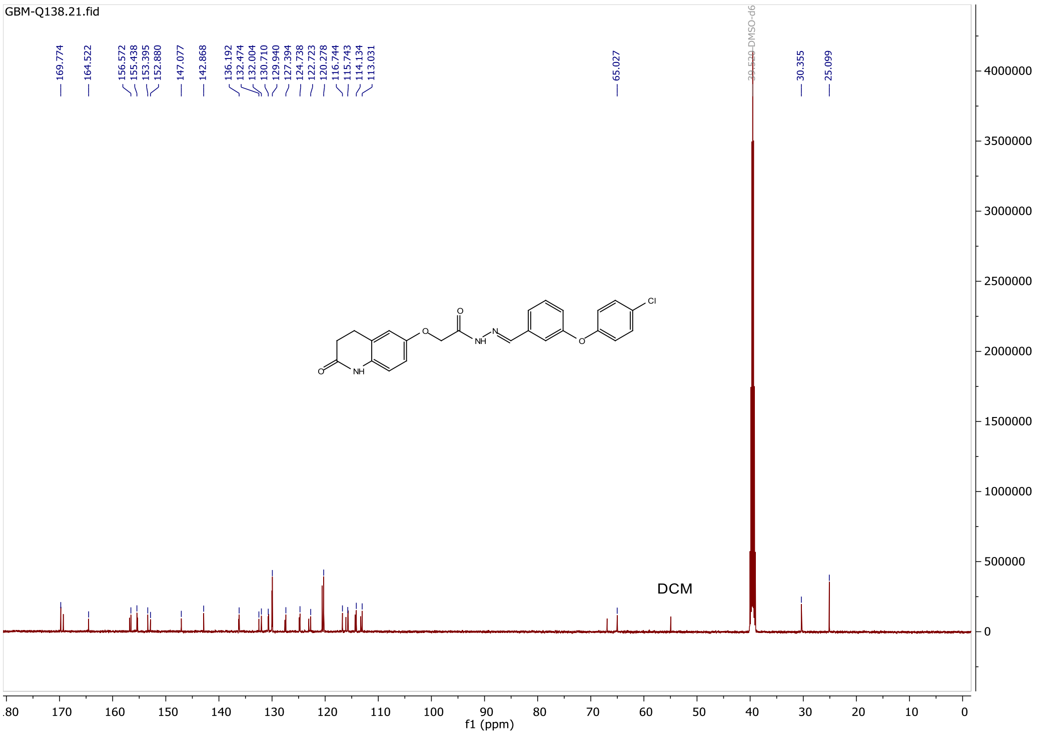

*Figure 58. ^{13}C-NMR of (E)-N'-(3-(4-chlorophenoxy)benzylidene)-2-((2-oxo-1,2,3,4-tetrahydroquinolin-6-yl)oxy)acetohydrazide (**4t**)*

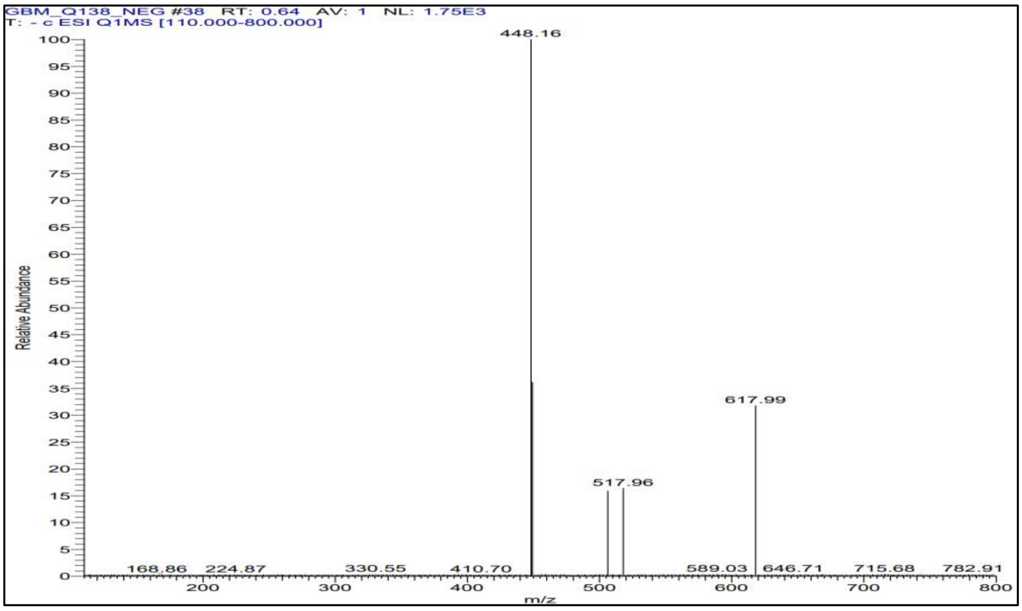

*Figure 59. Mass spectroscopy of (E)-N'-(3-(4-chlorophenoxy)benzylidene)-2-((2-oxo-1,2,3,4-tetrahydroquinolin-6-yl)oxy)acetohydrazide (**4t**)*

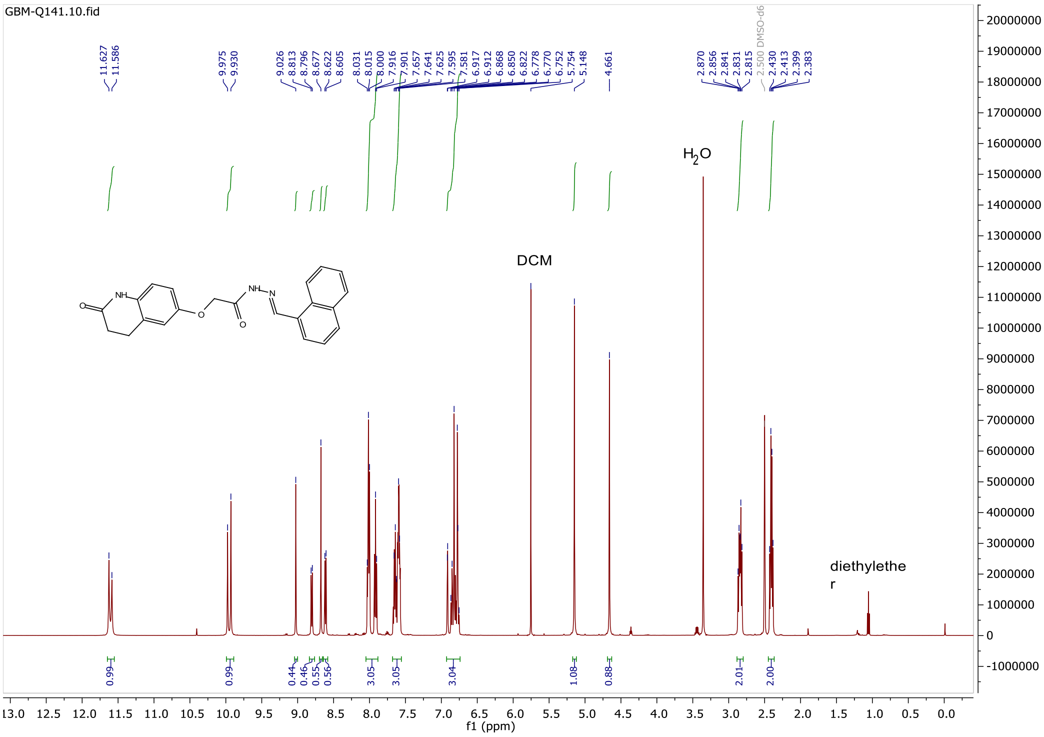

*Figure 60. ^{1}H-NMR of (E)-N'-(naphthalen-1-ylmethylene)-2-((2-oxo-1,2,3,4-tetrahydroquinolin-6-yl)oxy)acetohydrazide (**4u**)*

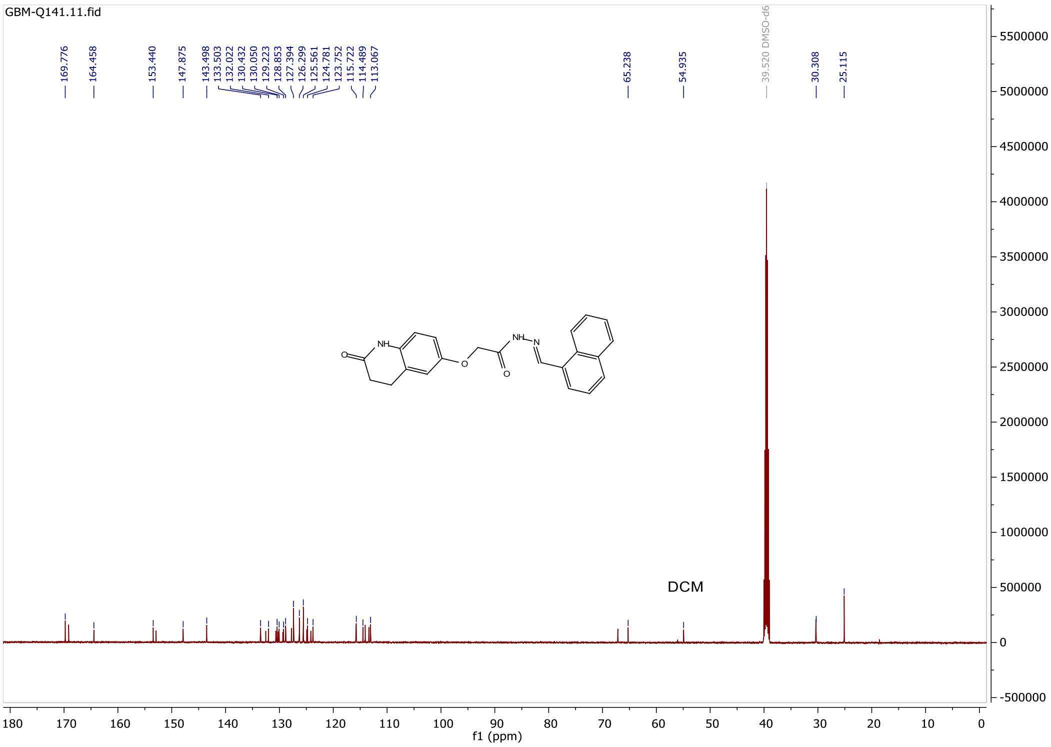

*Figure 61. ^{13}C-NMR of (E)-N'-(naphthalen-1-ylmethylene)-2-((2-oxo-1,2,3,4-tetrahydroquinolin-6-yl)oxy)acetohydrazide (**4u**)*

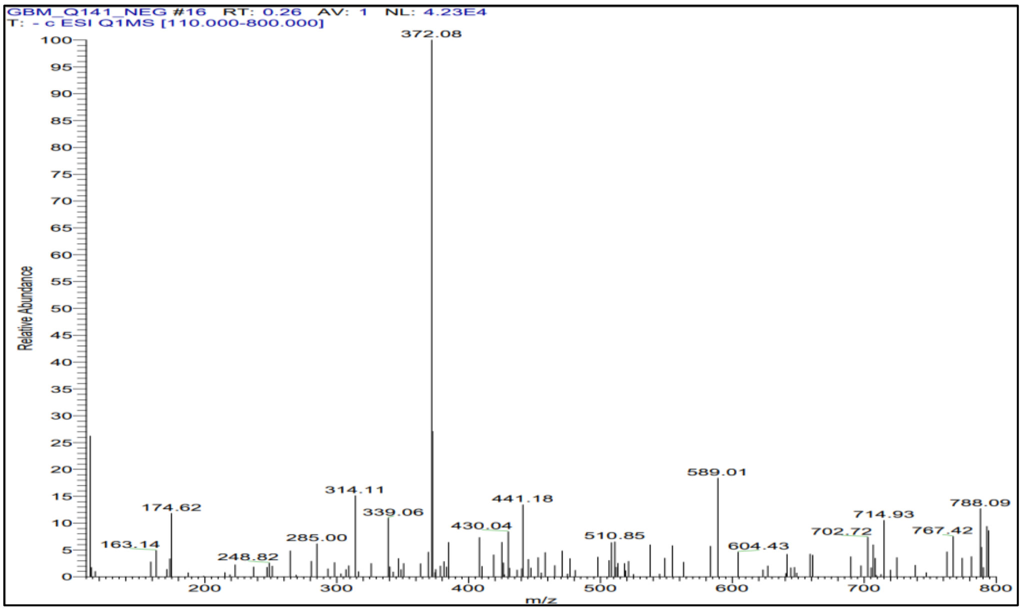

*Figure 62. Mass spectroscopy of (E)-N'-(naphthalen-1-ylmethylene)-2-((2-oxo-1,2,3,4-tetrahydroquinolin-6-yl)oxy)acetohydrazide (**4u**)*

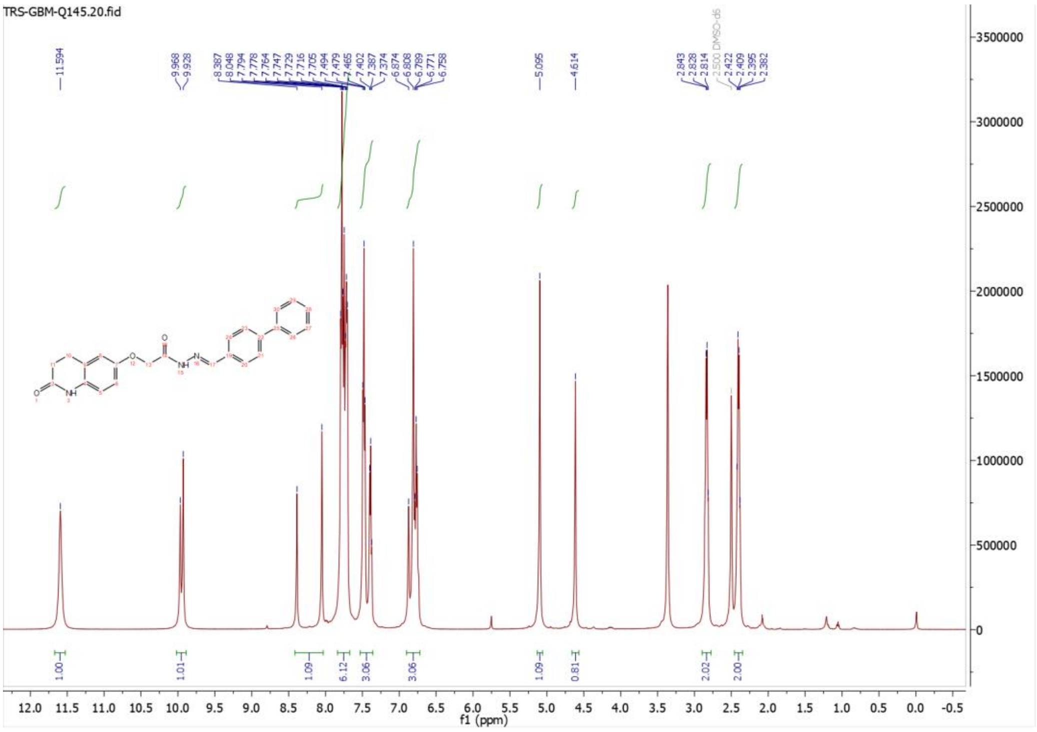

*Figure 63. ^{1}H-NMR of (E)-N'-([1,1'-biphenyl]-4-ylmethylene)-2-((2-oxo-1,2,3,4-tetrahydroquinolin-6-yl)oxy)acetohydrazide **(4v)***

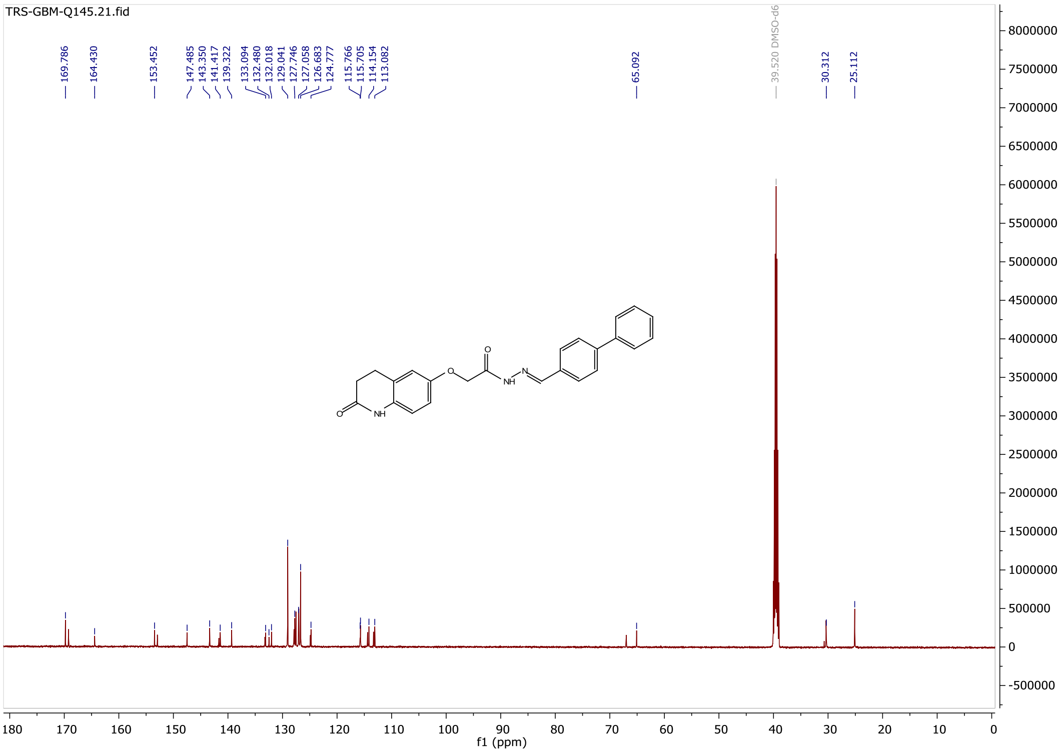

*Figure 64. ^{13}C-NMR of (E)-N'-([1,1'-biphenyl]-4-ylmethylene)-2-((2-oxo-1,2,3,4-tetrahydroquinolin-6-yl)oxy)acetohydrazide **(4v)***

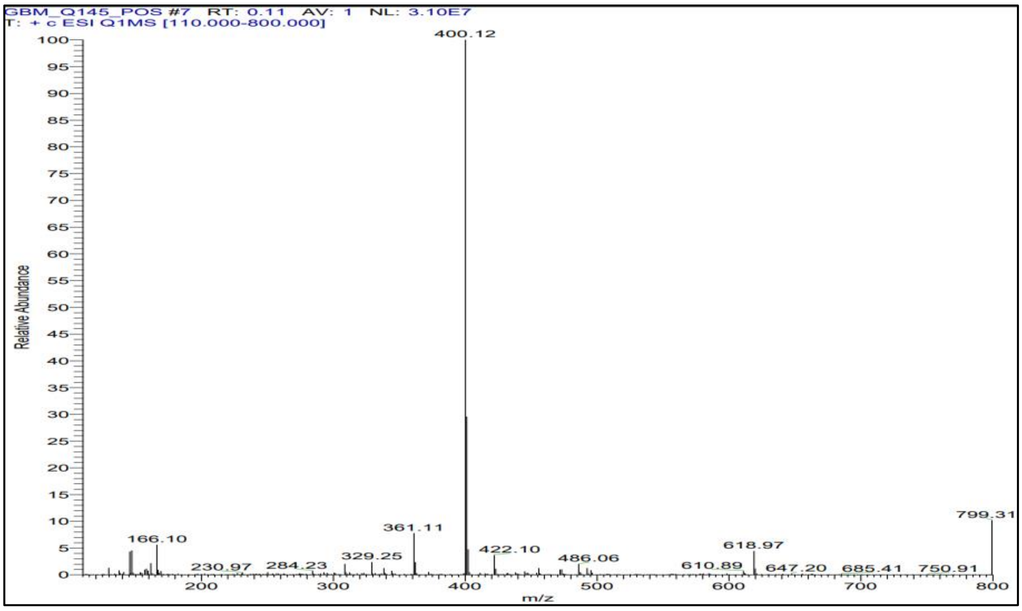

*Figure 65. Mass spectroscopy of (E)-N'-([1,1'-biphenyl]-4-ylmethylene)-2-((2-oxo-1,2,3,4-tetrahydroquinolin-6-yl)oxy)acetohydrazide (**4v**)*

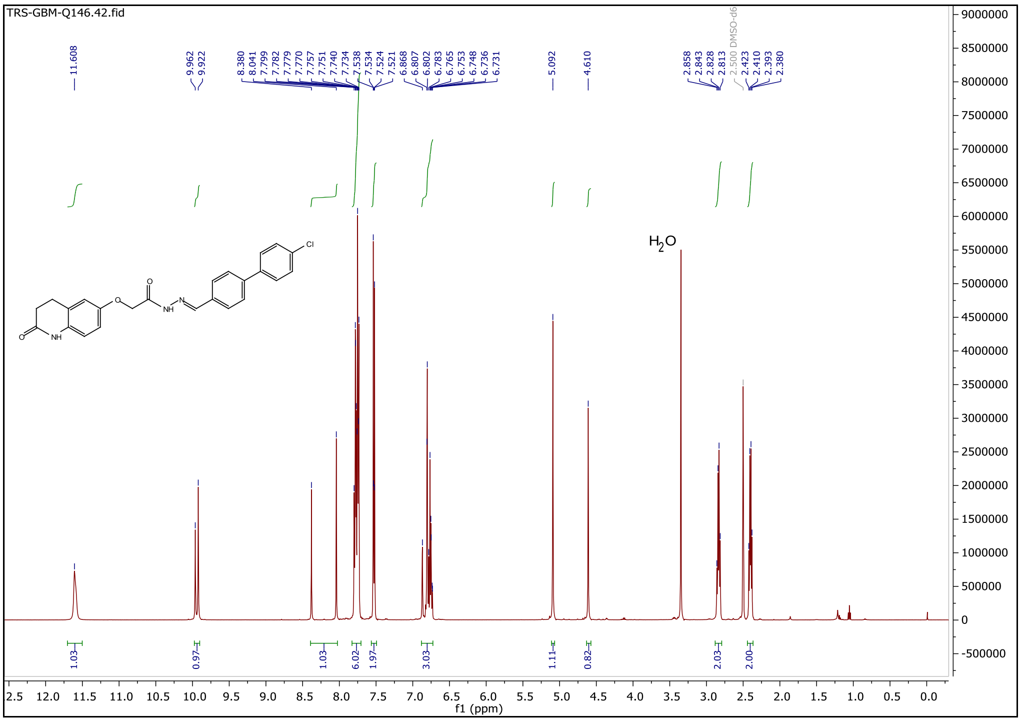

*Figure 66. ¹H-NMR of (E)-N'-((4'-chloro-[1,1'-biphenyl]-4-yl)methylene)-2-((2-oxo-1,2,3,4-tetrahydroquinolin-6-yl)oxy)acetohydrazide (**4w**)*

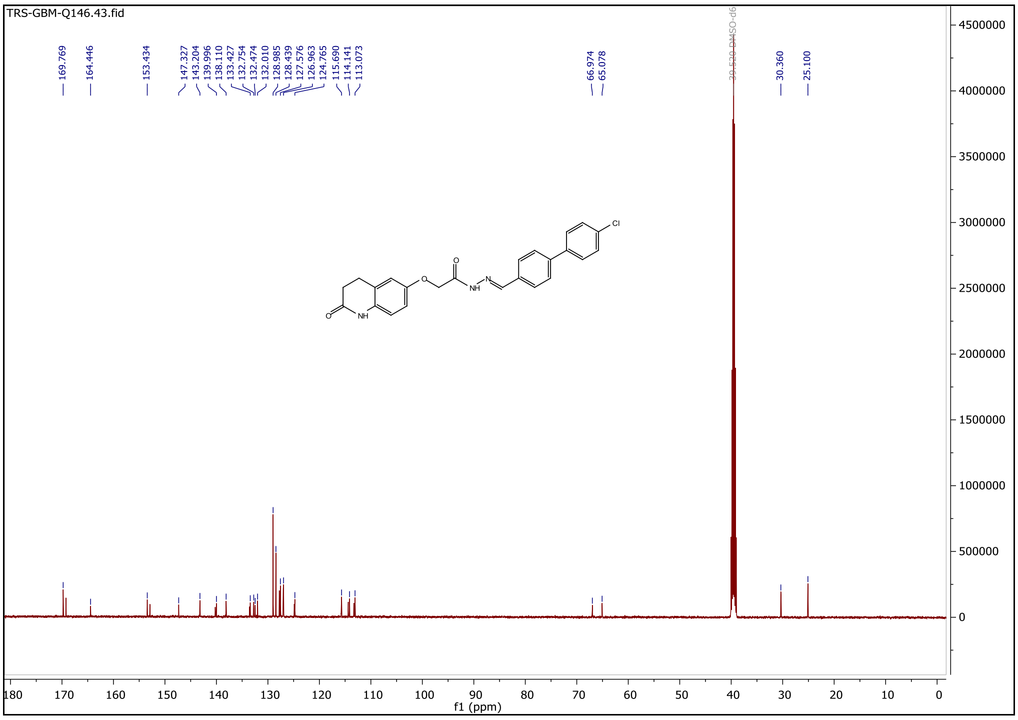

*Figure 67. ^{13}C-NMR of (E)-N'-((4'-chloro-[1,1'-biphenyl]-4-yl)methylene)-2-((2-oxo-1,2,3,4-tetrahydroquinolin-6-yl)oxy)acetohydrazide (**4w**)*

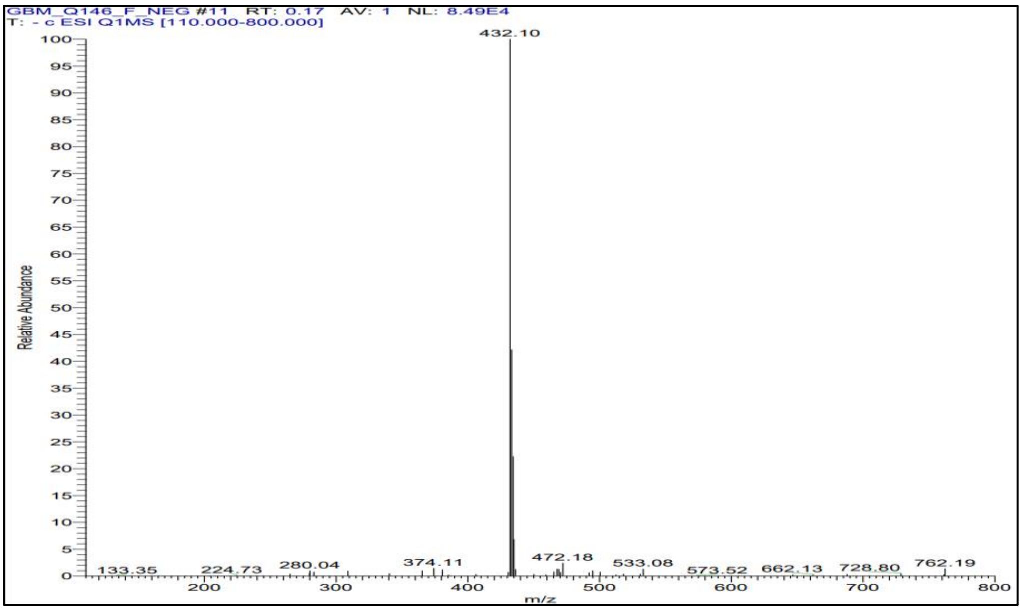

*Figure 68. Mass spectroscopy of (E)-N'-((4'-chloro-[1,1'-biphenyl]-4-yl)methylene)-2-((2-oxo-1,2,3,4-tetrahydroquinolin-6-yl)oxy)acetohydrazide (**4w**)*
